# Supplementary material for: Applying multimodal AI to physiological waveforms improves genetic prediction of cardiovascular traits
Source: Am J Hum Genet. 2025 Jun 20;112(7):1562–79. doi: 10.1016/j.ajhg.2025.05.015 (PMC12256885; doi:10.1016/j.ajhg.2025.05.015)
Supplement: Document S1. Figures S1–S38; Tables S1, S2, S5–S7, S9–S15, S17, S22–S28, S31, S32, S34, S35, S38, S39, and S41–S52; and supplemental note [file mmc1.pdf]

The American Journal of Human Genetics, Volume *112*

Supplemental information

Applying multimodal AI to physiological waveforms improves genetic prediction of cardiovascular traits

Yuchen Zhou, Justin Khasentino, Taedong Yun, Mahantesh I. Biradar, Jacqueline Shreibati, Dongbing Lai, Tae-Hwi Schwantes-An, Robert Luben, Zachary R. McCaw, Jorgen Engmann, Rui Providencia, Amand Floriaan Schmidt, Patricia B. Munroe, Howard Yang, Andrew Carroll, Anthony P. Khawaja, Cory Y. McLean, Babak Behsaz, and Farhad Hormozdiari

Supplemental Notes

Dataset acknowledgment

We are grateful to all the participants who have been part of the project and to the many members of the study teams at the University of Cambridge who have enabled this research. Indiana Biobank was made possible, in part, with support from the Indiana Clinical and Translational Sciences Institute funded, in part by Award Number UL1TR002529 from the National Institutes of Health, National Center for Advancing Translational Sciences, Clinical and Translational Sciences Award, and the National Center for Research Resources, Construction grant number RR020128 and the Lilly Endowment. The content is solely the responsibility of the authors and does not necessarily represent the official views of the National Institutes of Health. The authors acknowledge the Indiana University Pervasive Technology Institute for providing [HPC (Big Red II, Karst, Carbonate), visualization, database, storage, or consulting] resources that have contributed to the research results reported within this paper. BWHHS is supported by funding from the British Heart Foundation and the Department of Health Policy Research Programme (England).

M-REGLE model architecture (ECG + PPG)

Encoder

Layer (type)	Output Shape	Param #
vae_encoder_input (InputLayer)	[(None, 700, 1)]	0
vae_encoder_conv1 (Conv1D)	(None, 700, 16)	128
leaky_re_lu (LeakyReLU)	(None, 700, 16)	0
vae_encoder_pooling1 (MaxPooling1D)	(None, 350, 16)	0
vae_encoder_conv2 (Conv1D)	(None, 350, 16)	1808
leaky_re_lu_1 (LeakyReLU)	(None, 350, 16)	0
vae_encoder_pooling2 (MaxPooling1D)	(None, 175, 16)	0
vae_encoder_conv3 (Conv1D)	(None, 175, 16)	1808
leaky_re_lu_2 (LeakyReLU)	(None, 175, 16)	0
vae_encoder_pooling3 (MaxPooling1D)	(None, 88, 16)	0
vae_encoder_conv4 (Conv1D)	(None, 88, 16)	1808
leaky_re_lu_3 (LeakyReLU)	(None, 88, 16)	0
vae_encoder_pooling4 (MaxPooling1D)	(None, 44, 16)	0
vae_encoder_conv5 (Conv1D)	(None, 44, 16)	1808
leaky_re_lu_4 (LeakyReLU)	(None, 44, 16)	0
vae_encoder_pooling5 (MaxPooling1D)	(None, 22, 16)	0
vae_encoder_flatten (Flatten)	(None, 352)	0
vae_encoder_dense1 (Dense)	(None, 64)	22592
vae_encoder_dense2 (Dense)	(None, 32)	2080
z_mean (Dense)	(None, 12)	396
z_log_var (Dense)	(None, 12)	396
gaussian_sampling (GaussianSampling)	(None, 12)	0

Total params: 32824

Trainable params: 32824

Decoder

Layer (type)	Output Shape	Param #
decoder_input (InputLayer)	[(None, 12)]	0
decoder_dense1 (Dense)	(None, 32)	416
decoder_dense2 (Dense)	(None, 64)	2112
decoder_dense3 (Dense)	(None, 352)	22880
reshape (Reshape)	(None, 22, 16)	0
decoder_upsample1 (UpSampling1D)	(None, 44, 16)	0
decoder_trans_conv1 (Conv1DTranspose)	(None, 44, 16)	1808
leaky_re_lu_5 (LeakyReLU)	(None, 44, 16)	0
decoder_upsample2 (UpSampling1D)	(None, 88, 16)	0
decoder_trans_conv2 (Conv1DTranspose)	(None, 88, 16)	1808
leaky_re_lu_6 (LeakyReLU)	(None, 88, 16)	0
decoder_upsample3 (UpSampling1D)	(None, 176, 16)	0
decoder_trans_conv3 (Conv1DTranspose)	(None, 176, 16)	1808
leaky_re_lu_7 (LeakyReLU)	(None, 176, 16)	0
decoder_upsample4 (UpSampling1D)	(None, 352, 16)	0
decoder_trans_conv4 (Conv1DTranspose)	(None, 352, 16)	1808
leaky_re_lu_8 (LeakyReLU)	(None, 352, 16)	0
decoder_upsample5 (UpSampling1D)	(None, 704, 16)	0
decoder_trans_conv5 (Conv1DTranspose)	(None, 704, 1)	113
leaky_re_lu_9 (LeakyReLU)	(None, 704, 1)	0
cropping1d (Cropping1D)	(None, 700, 1)	0

Total params: 32753

Trainable params: 32753

M-REGLE model architecture (12 Lead ECG)

Encoder

Layer (type)	Output Shape	Param #
vae_encoder_input (InputLayer)	[(None, 600, 12)]	0
vae_encoder_conv1 (Conv1D)	(None, 600, 32)	1184
leaky_re_lu (LeakyReLU)	(None, 600, 32)	0
vae_encoder_pooling1 (MaxPooling1D)	(None, 300, 32)	0
vae_encoder_conv2 (Conv1D)	(None, 300, 32)	3104
leaky_re_lu_1 (LeakyReLU)	(None, 300, 32)	0
vae_encoder_pooling2 (MaxPooling1D)	(None, 150, 32)	0
vae_encoder_conv3 (Conv1D)	(None, 150, 32)	3104
leaky_re_lu_2 (LeakyReLU)	(None, 150, 32)	0
vae_encoder_pooling3 (MaxPooling1D)	(None, 75, 32)	0
vae_encoder_flatten (Flatten)	(None, 2400)	0
vae_encoder_dense1 (Dense)	(None, 512)	1229312
z_mean (Dense)	(None, 96)	49248
z_log_var (Dense)	(None, 96)	49248
gaussian_sampling (GaussianSampling)	(None, 96)	0

Total params: 1335200

Trainable params: 1335200

Decoder

Layer (type)	Output Shape	Param #
decoder_input (InputLayer)	[(None, 96)]	0
decoder_dense1 (Dense)	(None, 512)	49664
decoder_dense2 (Dense)	(None, 2400)	1231200
reshape (Reshape)	(None, 75, 32)	0
decoder_upsample1 (UpSampling1D)	(None, 150, 32)	0
decoder_trans_conv1 (Conv1DTranspose)	(None, 150, 32)	3104
leaky_re_lu_3 (LeakyReLU)	(None, 150, 32)	0
decoder_upsample2 (UpSampling1D)	(None, 300, 32)	0
decoder_trans_conv2 (Conv1DTranspose)	(None, 300, 32)	3104
leaky_re_lu_4 (LeakyReLU)	(None, 300, 32)	0
decoder_upsample3 (UpSampling1D)	(None, 600, 32)	0
decoder_trans_conv3 (Conv1DTranspose)	(None, 600, 12)	1164
leaky_re_lu_5 (LeakyReLU)	(None, 600, 12)	0
cropping1d (Cropping1D)	(None, 600, 12)	0

Total params: 1288236

Trainable params: 1288236

Supplemental Figures

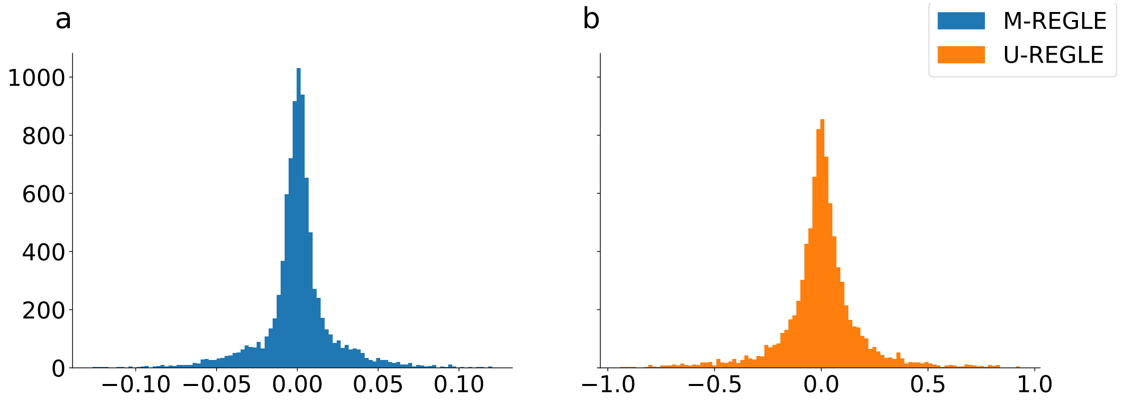

Figure S1: **Correlation coefficients (non-diagonal) histogram of M-REGLE and U-REGLE embeddings of 12-lead ECG.** a) Histogram of non-diagonal correlation coefficient matrices of M-REGLE 12-lead ECG embeddings. Most of the non-diagonal coefficients fall in [-0.1,0.1], which shows that the coordinates of M-REGLE embeddings are mostly orthogonalized. b) Histogram of non-diagonal correlation coefficient matrices of U-REGLE 12-lead ECG embeddings.

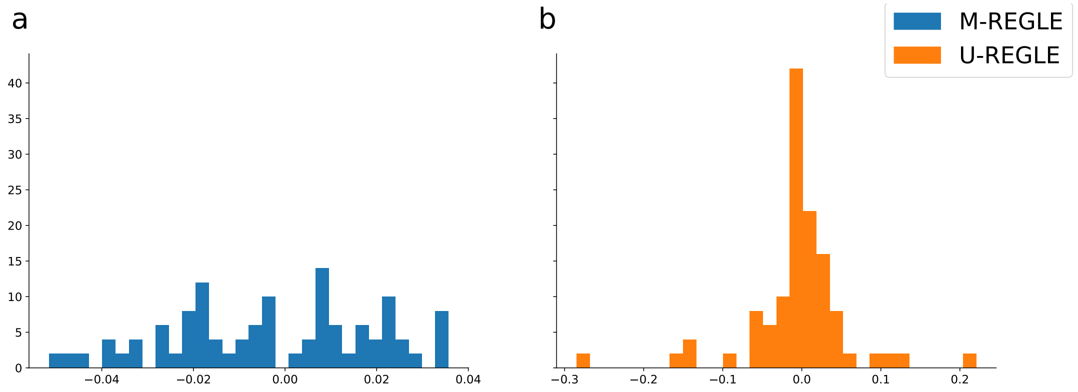

Figure S2: **Correlation coefficients (non-diagonal) histogram of M-REGLE and U-REGLE embeddings of ECG lead I + PPG.** a) Histogram of non-diagonal correlation coefficient matrices of M-REGLE ECG lead I + PPG embeddings. Most of the non-diagonal coefficients fall in [-0.05,0.05], which shows that the coordinates of M-REGLE embeddings are mostly orthogonalized. b) Histogram of non-diagonal correlation coefficient matrices of U-REGLE ECG lead I + PPG embeddings.

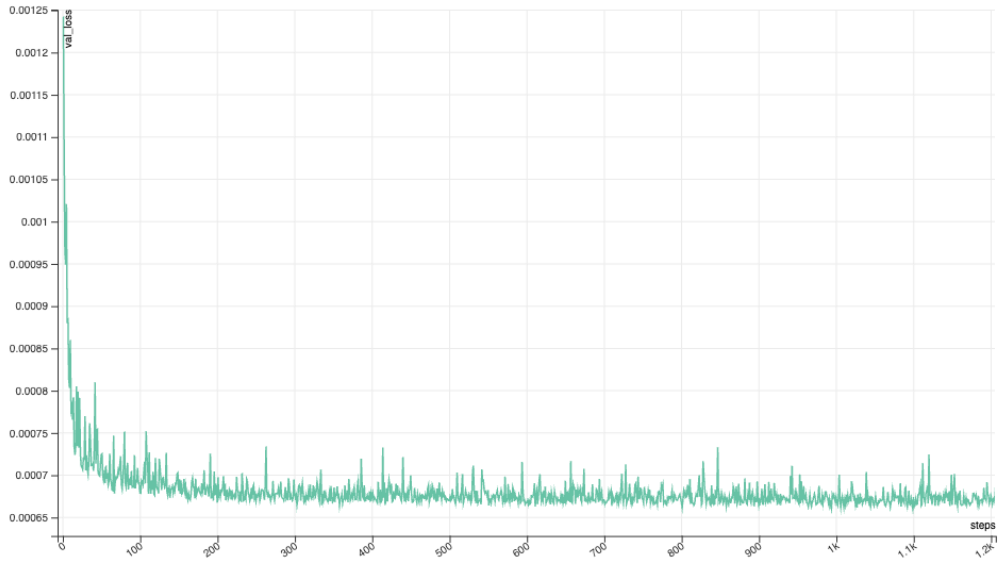

Figure S3: **Validation loss curve of training unimodal PPG model for up to 1200 epochs.** We trained the U-REGLE models for the same number of epochs with M-REGLE models and plotted the validation loss curve. The curve plateaued before 300 epochs which is the maximum number of epochs we used for U-REGLE models in this paper.

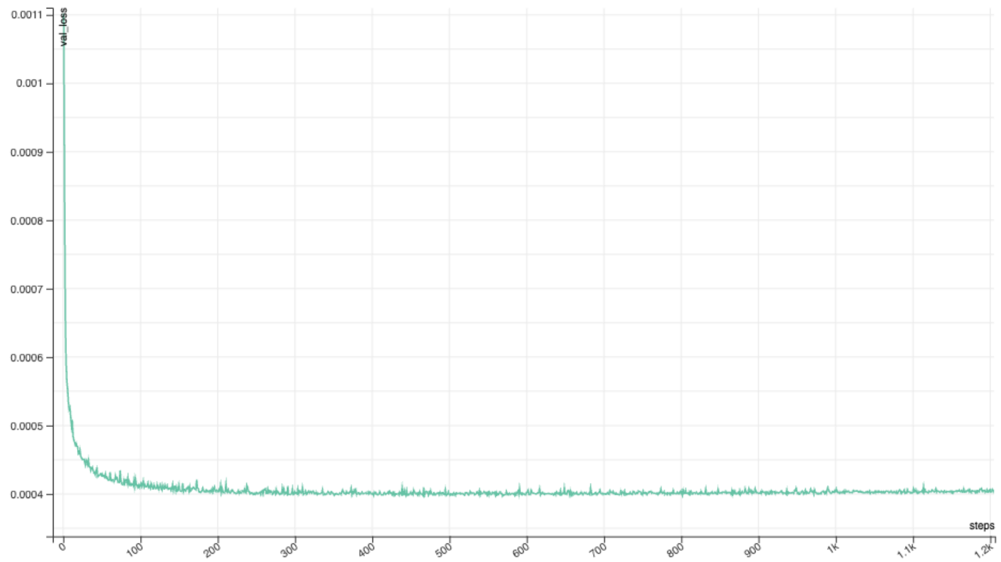

Figure S4: **Validation loss curve of training unimodal ECG lead I model for up to 1200 epochs.** See details in Figure S3.

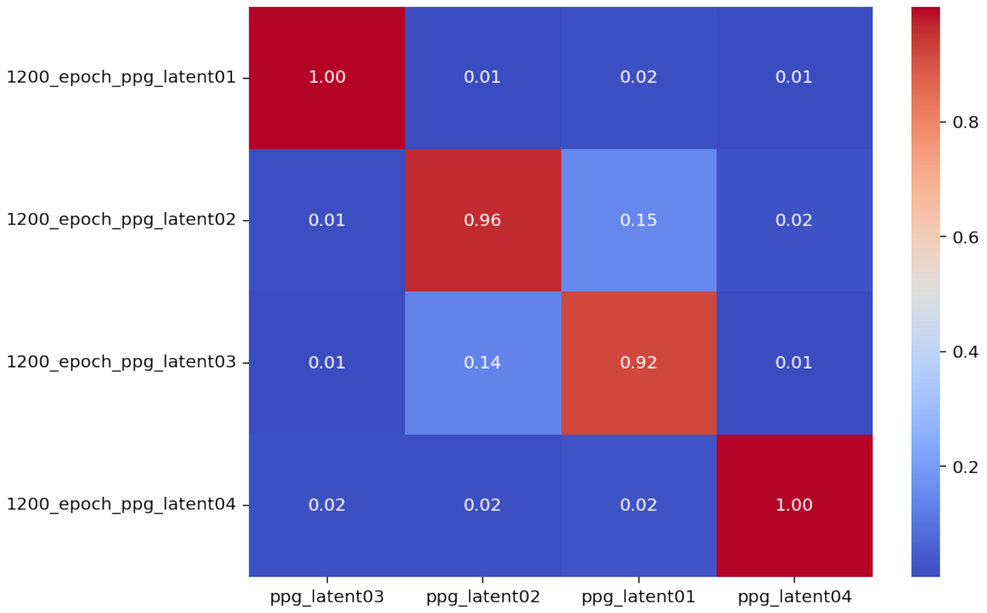

Figure S5: **Correlation coefficient matrix of the best unimodal PPG embeddings within 300 training epochs and the best unimodal PPG embeddings within 1200 training epochs.** To further show that the U-REGLE embeddings and downstream analyses remain stable when increasing the training epochs, we extracted the embeddings from the best model we got by training the model for up to 1200 epochs (see Figure S3), and compared them with the embeddings from the best model within 300 epochs (the ones we used in the paper). We got a correlation coefficient matrix close to an identity matrix, by reordering the coordinates of the embeddings and running Spearman correlation on the 1200-epoch embeddings and the 300-epoch embeddings.

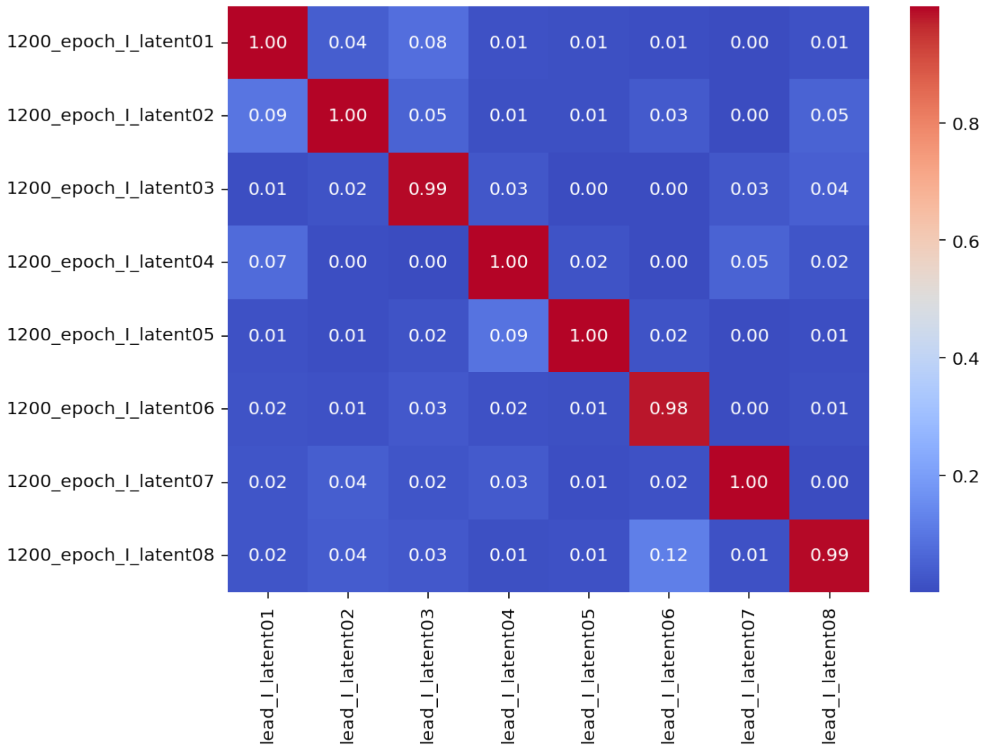

Figure S6: **Correlation coefficient matrix of the best unimodal ECG lead I embeddings within 300 training epochs and the best unimodal ECG lead I embeddings within 1200 training epochs.** See details in Figure S5)

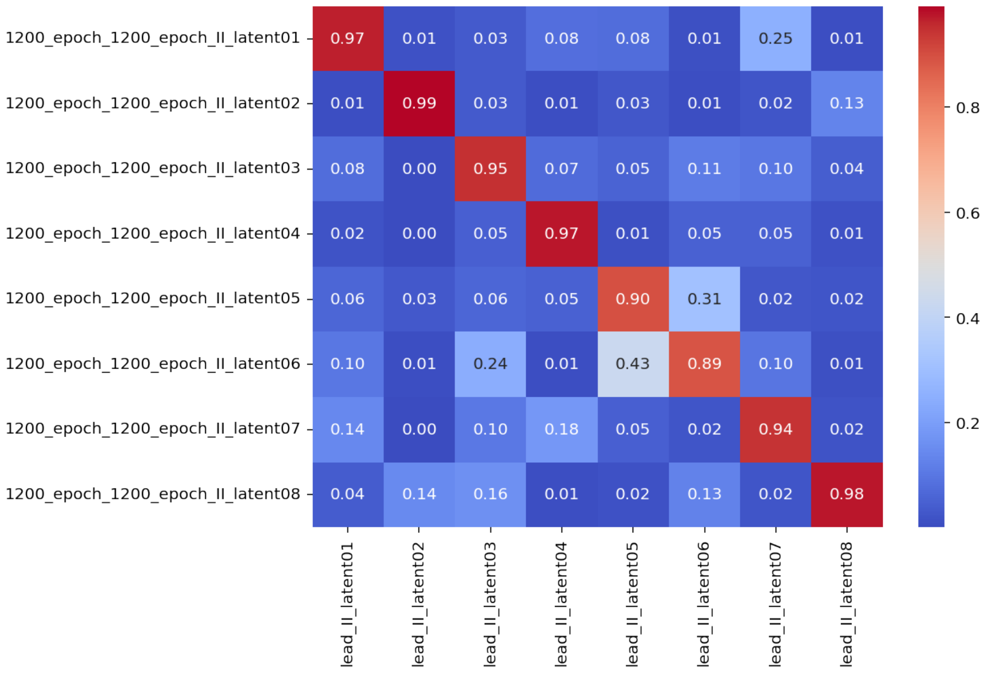

Figure S7: **Correlation coefficient matrix of the best unimodal ECG lead II embeddings within 300 training epochs and the best unimodal ECG lead II embeddings within 1200 training epochs.** See details in Figure S5)

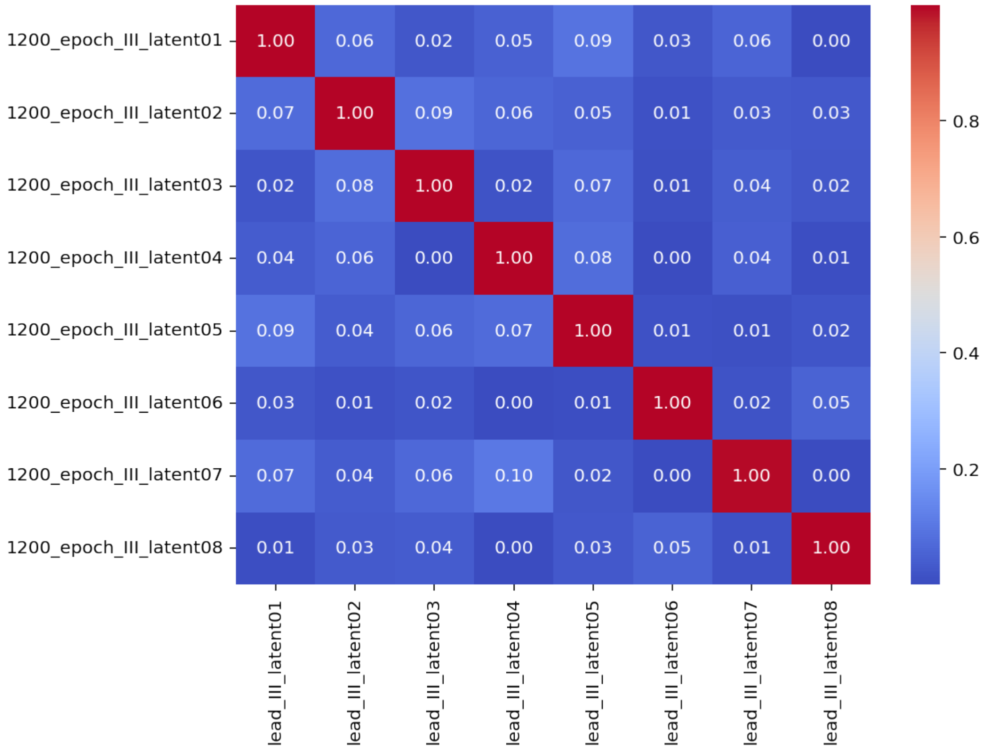

Figure S8: **Correlation coefficient matrix of the best unimodal ECG lead III embeddings within 300 training epochs and the best unimodal ECG lead III embeddings within 1200 training epochs.** See details in Figure S5)

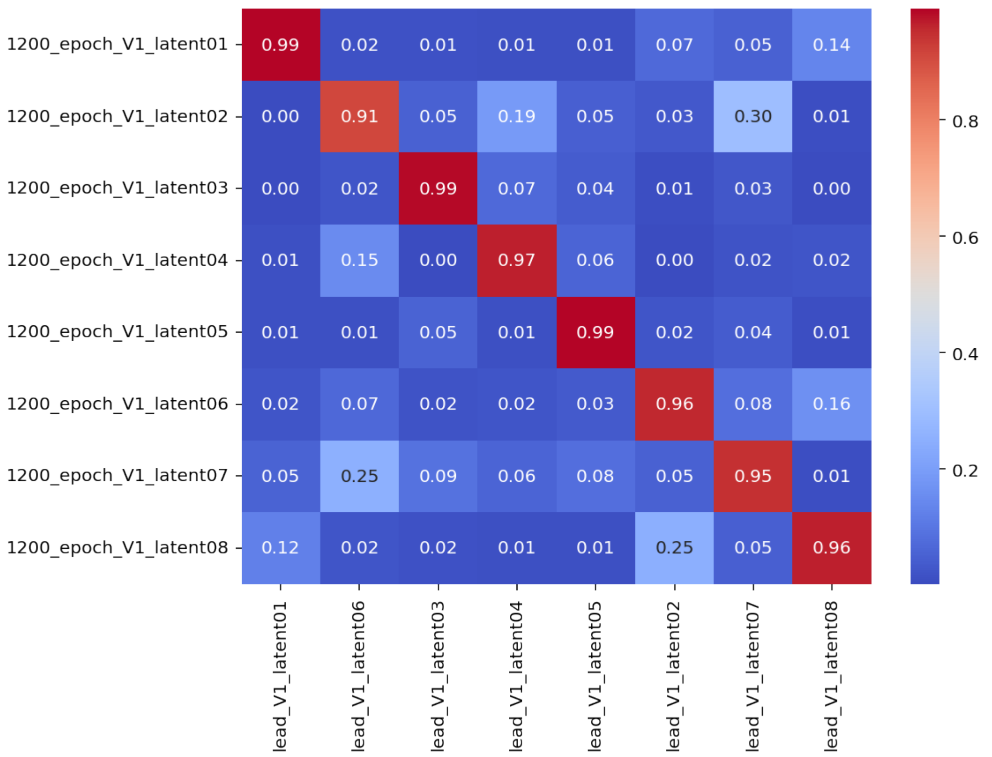

Figure S9: **Correlation coefficient matrix of the best unimodal ECG lead V1 embeddings within 300 training epochs and the best unimodal ECG lead V1 embeddings within 1200 training epochs.** See details in Figure S5)

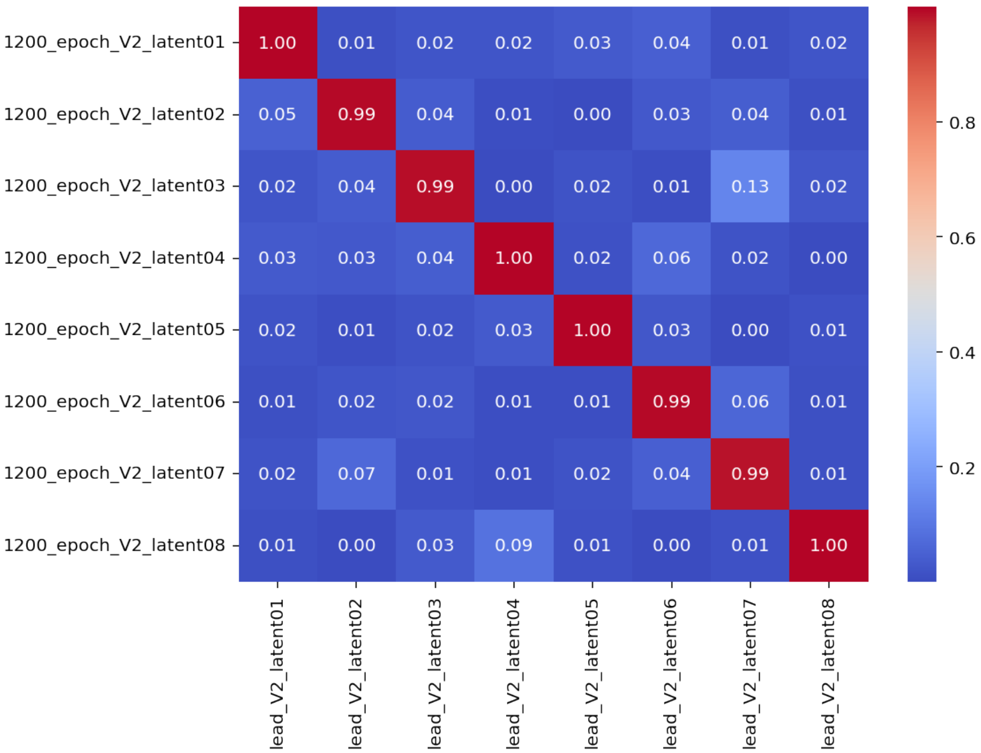

Figure S10: **Correlation coefficient matrix of the best unimodal ECG lead V2 embeddings within 300 training epochs and the best unimodal ECG lead V2 embeddings within 1200 training epochs.** See details in Figure S5)

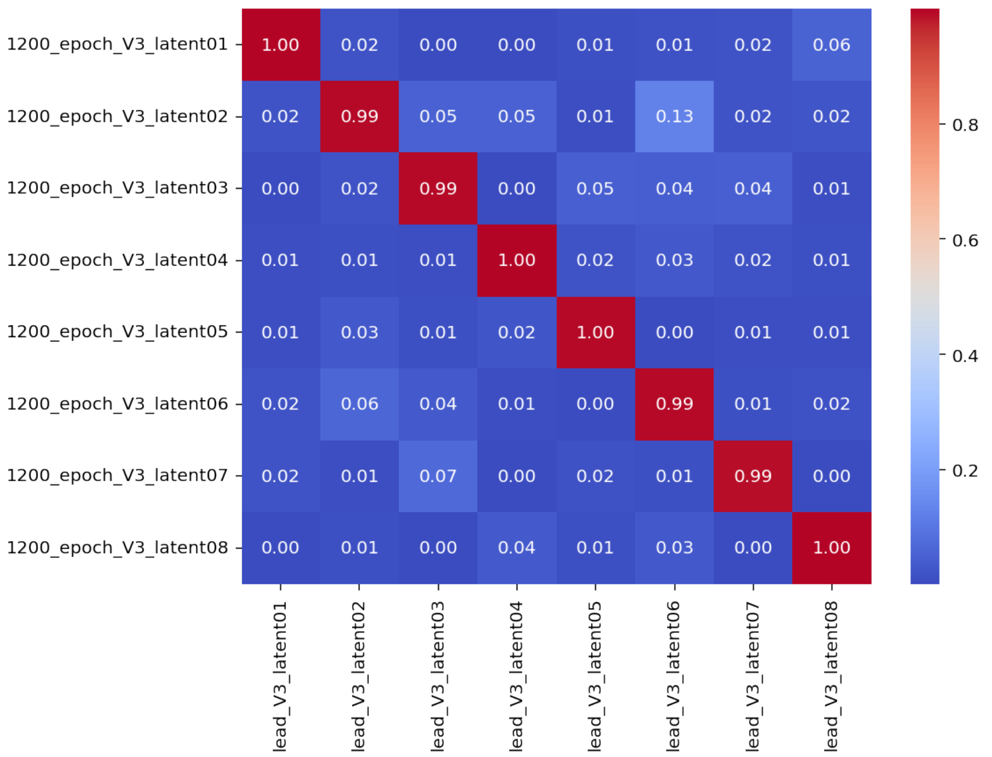

Figure S11: **Correlation coefficient matrix of the best unimodal ECG lead V3 embeddings within 300 training epochs and the best unimodal ECG lead V3 embeddings within 1200 training epochs.** See details in Figure S5)

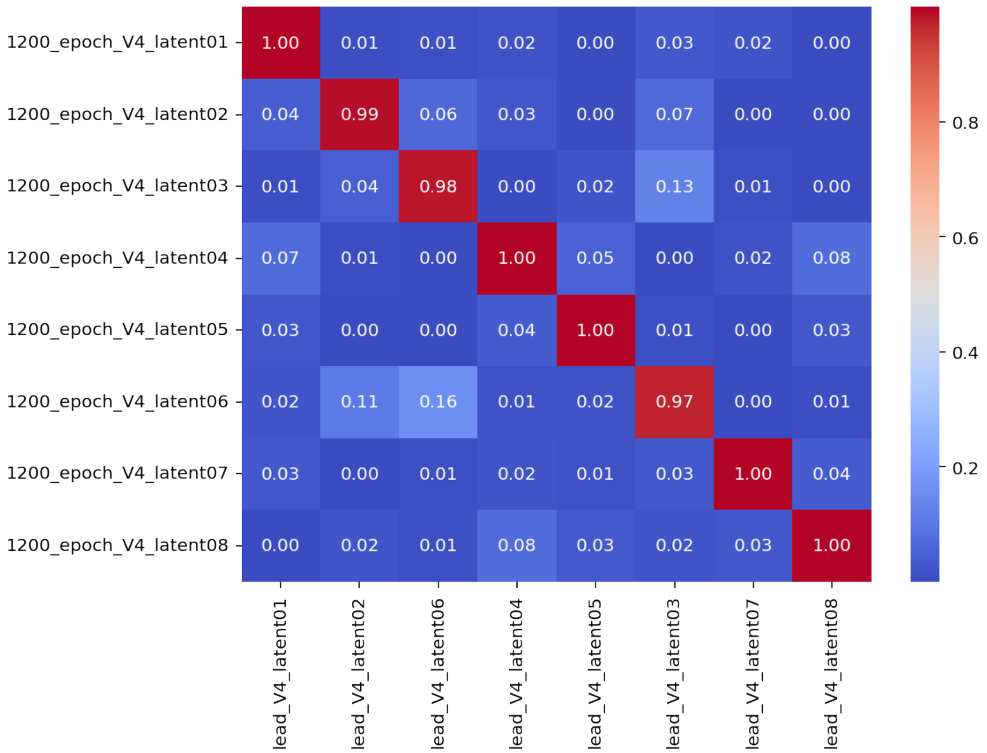

Figure S12: **Correlation coefficient matrix of the best unimodal ECG lead V4 embeddings within 300 training epochs and the best unimodal ECG lead V4 embeddings within 1200 training epochs.** See details in Figure S5)

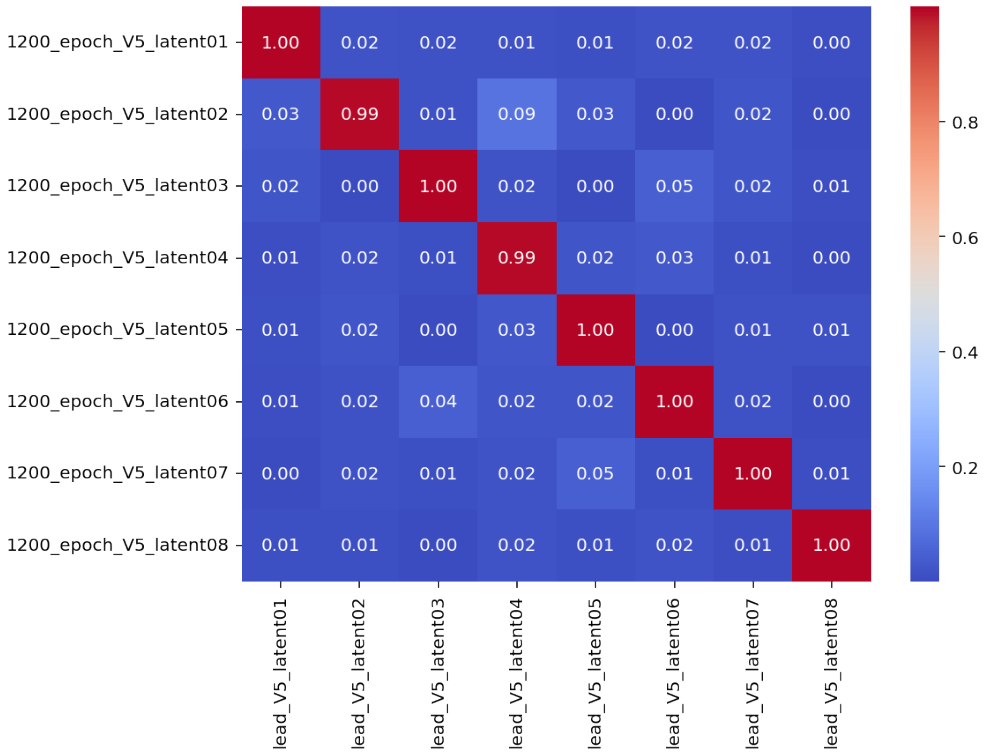

Figure S13: **Correlation coefficient matrix of the best unimodal ECG lead V5 embeddings within 300 training epochs and the best unimodal ECG lead V5 embeddings within 1200 training epochs.** See details in Figure S5)

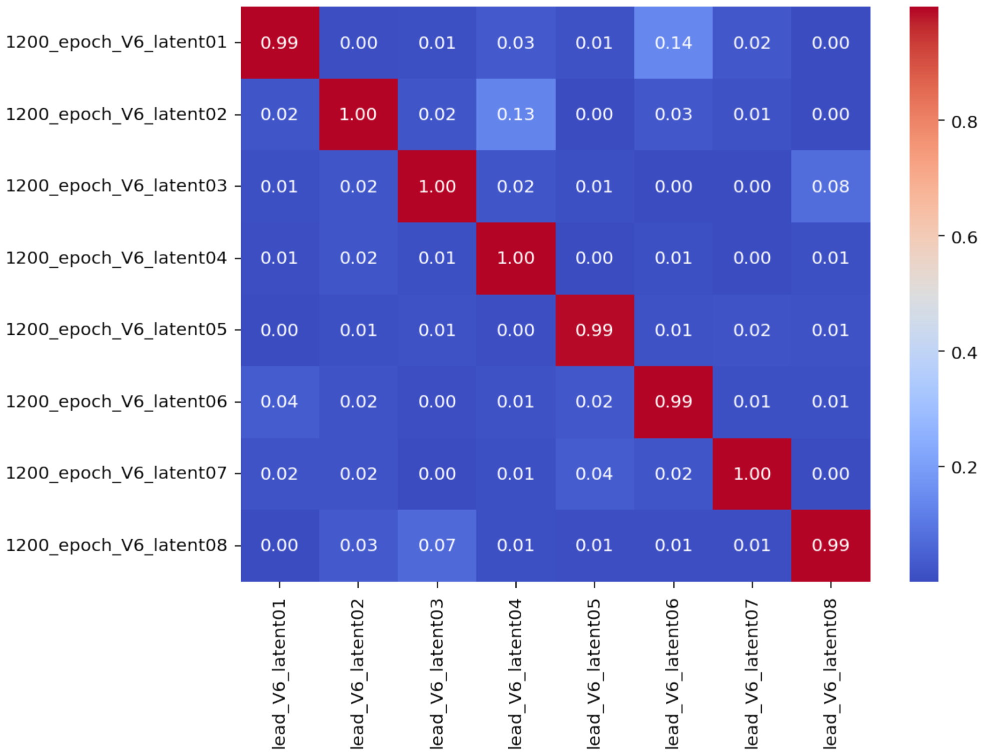

Figure S14: **Correlation coefficient matrix of the best unimodal ECG lead V6 embeddings within 300 training epochs and the best unimodal ECG lead V6 embeddings within 1200 training epochs.** See details in Figure S5)

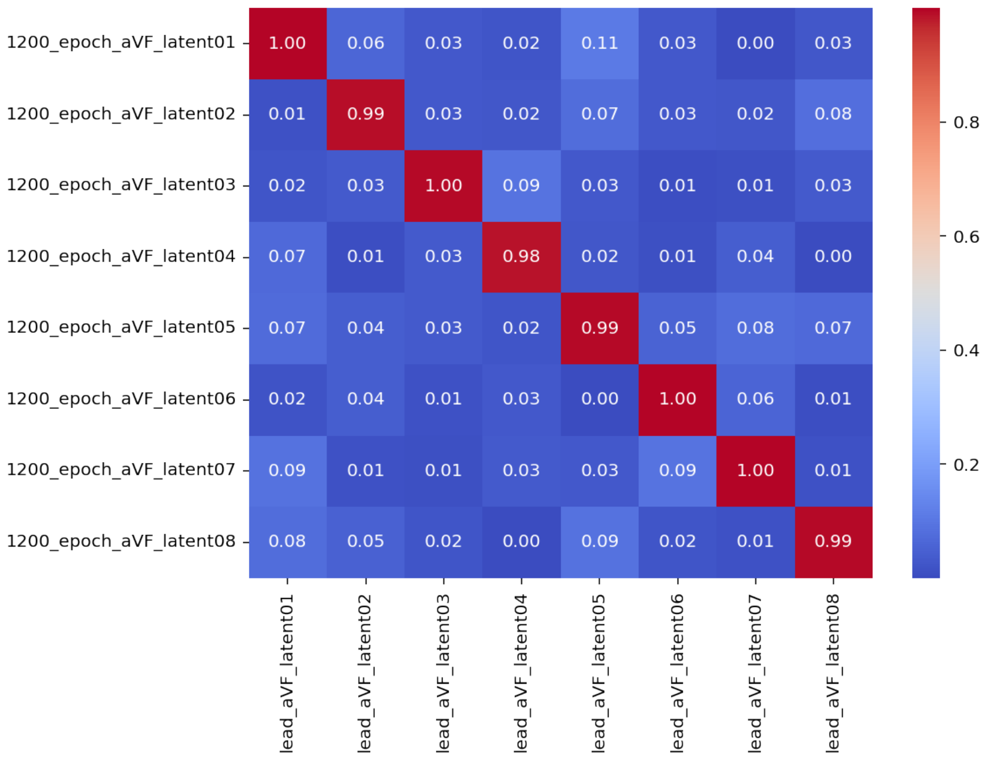

Figure S15: **Correlation coefficient matrix of the best unimodal ECG lead aVF embeddings within 300 training epochs and the best unimodal ECG lead aVF embeddings within 1200 training epochs.** See details in Figure S5)

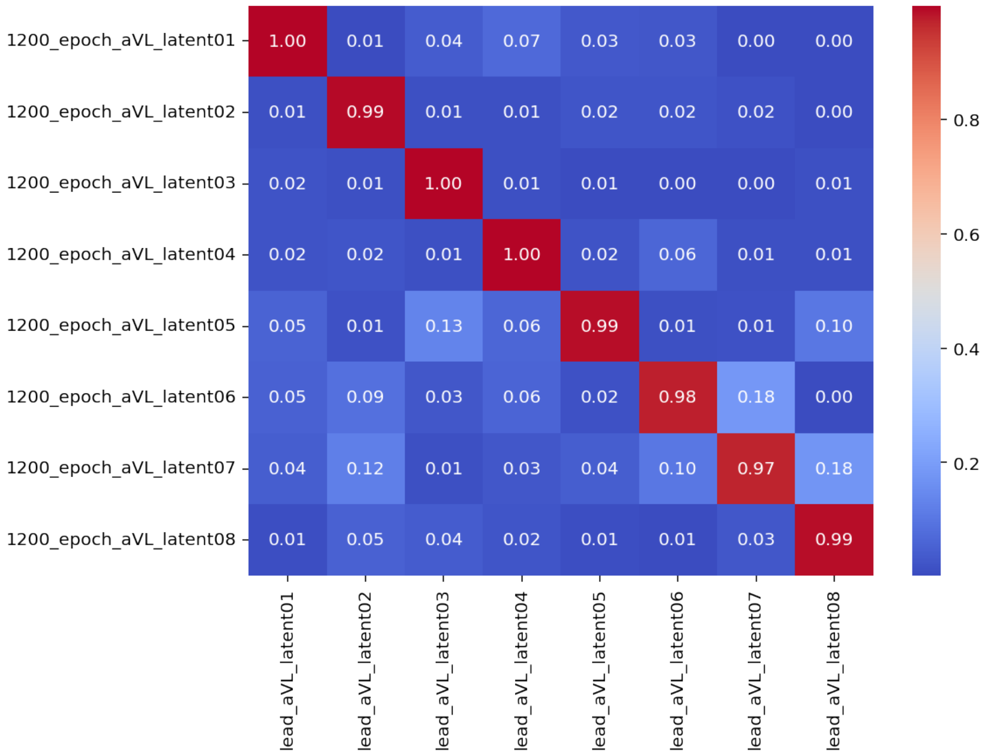

Figure S16: **Correlation coefficient matrix of the best unimodal ECG lead aVL embeddings within 300 training epochs and the best unimodal ECG lead aVL embeddings within 1200 training epochs.** See details in Figure S5)

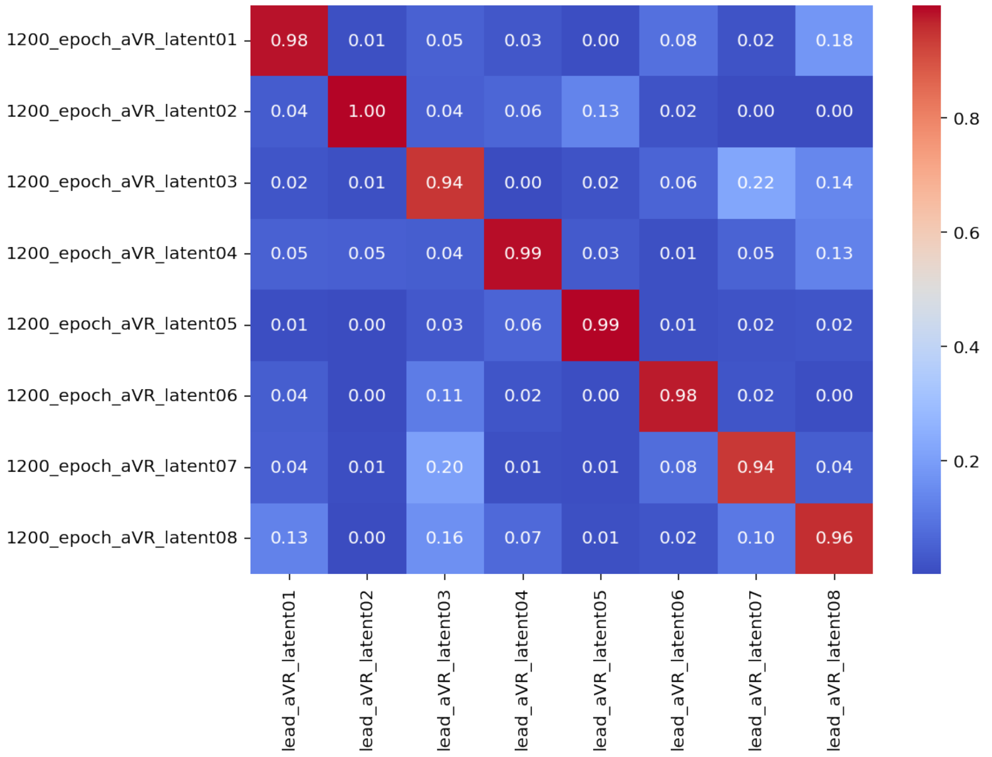

Figure S17: **Correlation coefficient matrix of the best unimodal ECG lead aVR embeddings within 300 training epochs and the best unimodal ECG lead aVR embeddings within 1200 training epochs.** See details in Figure S5)

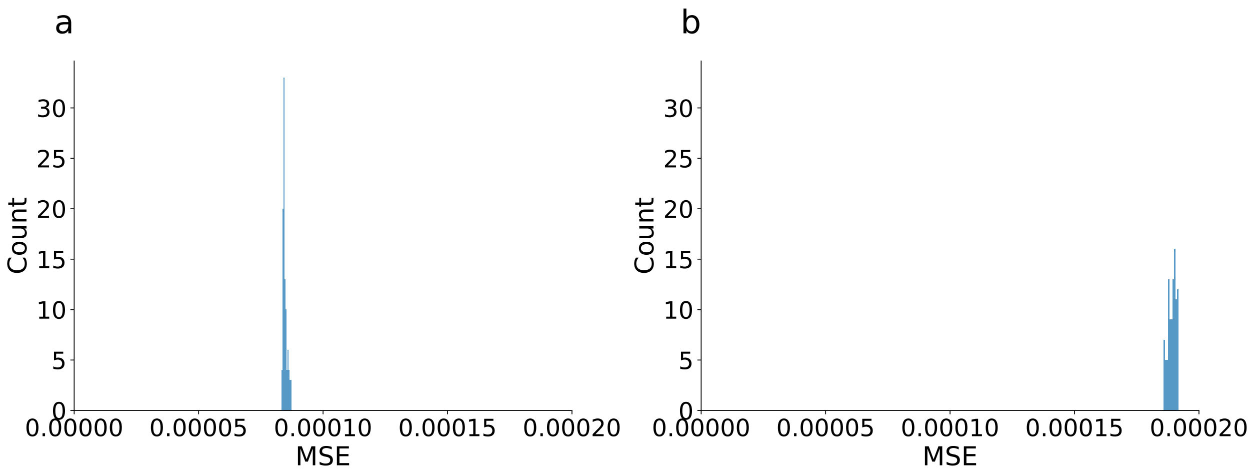

Figure S18: **M-REGLE's performance is robust against random seeds.** a) Histogram of MSEs of 12-lead ECG models (96 latent dimensions) trained on 100 different random seeds. b) Histogram of MSEs of ECGPPG models (12 latent dimensions) trained on 100 different random seeds.

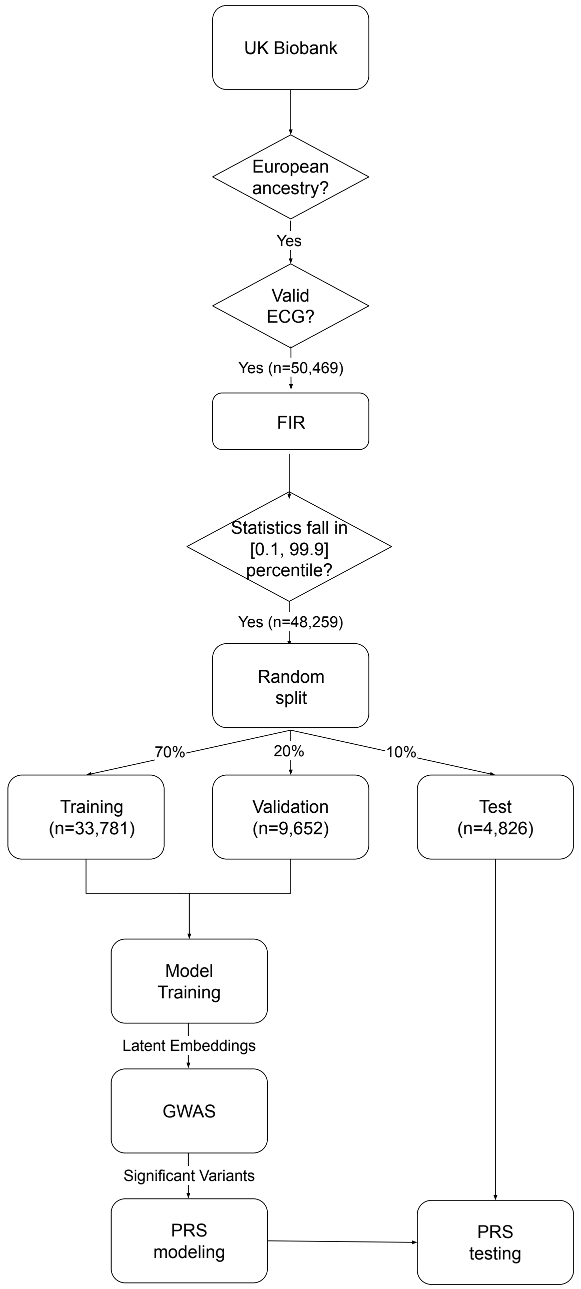

Figure S19: **An overview of UK Biobank 12-lead ECG used in this study** Our initial dataset consists of all European-ancestry in UK Biobank (n=435,766). We considered all individuals with valid 12-lead ECG waveform at instance 2 (n=50,469). After the preprocessing and quality control steps, we split the dataset to training (70%) validation (20%) and test (10%) sets. We use all individuals in training and validation sets for GWAS analysis, and test set for reporting the PRS results.

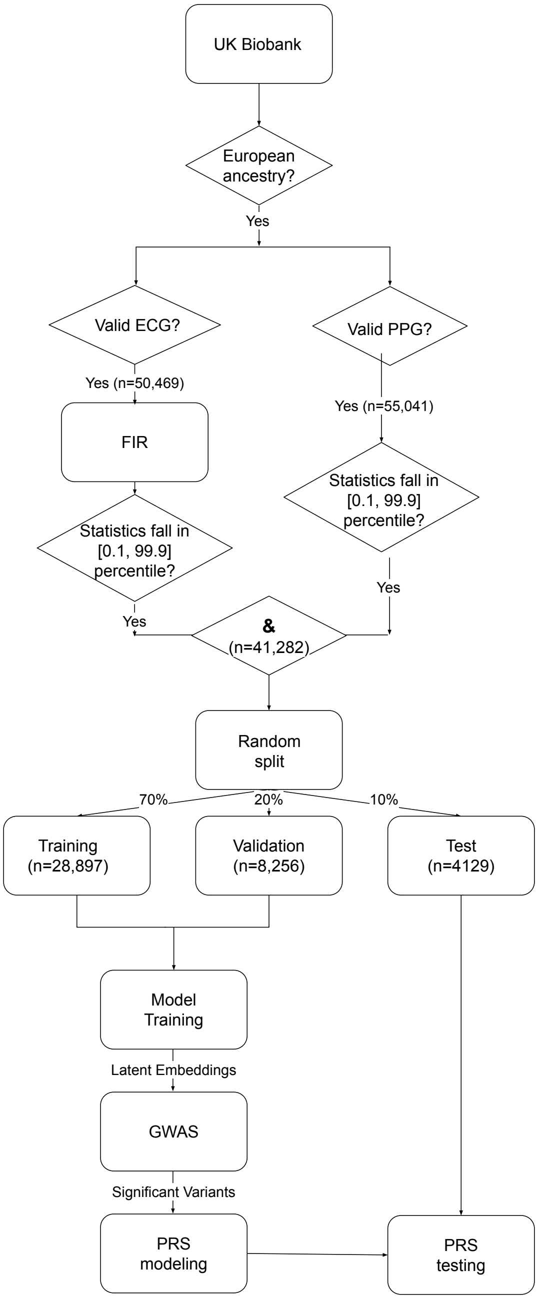

Figure S20: **An overview of UK Biobank ECG lead I and PPG used in this study** Our initial dataset consists of all European-ancestry in UK Biobank (n=435,766). We considered all individuals with valid ECG lead I (n=50,469) and PPG (n=55,041) waveform at instance 2. After the preprocessing and quality control steps, we get all the individuals with both ECG and PPG qualified. We split the dataset to training (70%) validation (20%) and test (10%) sets. We use all individuals in training and validation sets for GWAS analysis, and test set for reporting the PRS results.

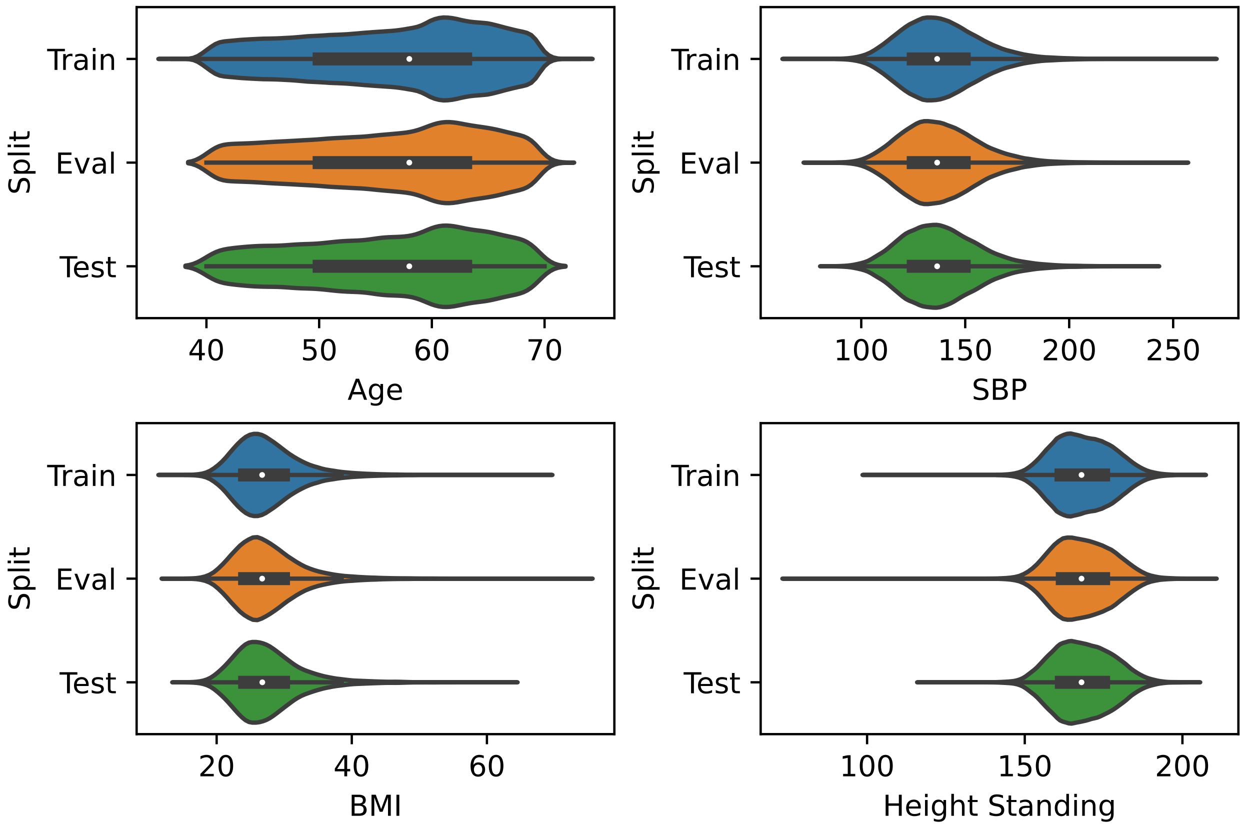

Figure S21: **UK Biobank phenotype distribution for different data splits** We plot the age, SBP (systolic blood pressure), BMI (body mass index), and Height standing for train, eval, and test datasets in UKB. We observed that in most cases all datasets tend to have similar phenotypic distribution.

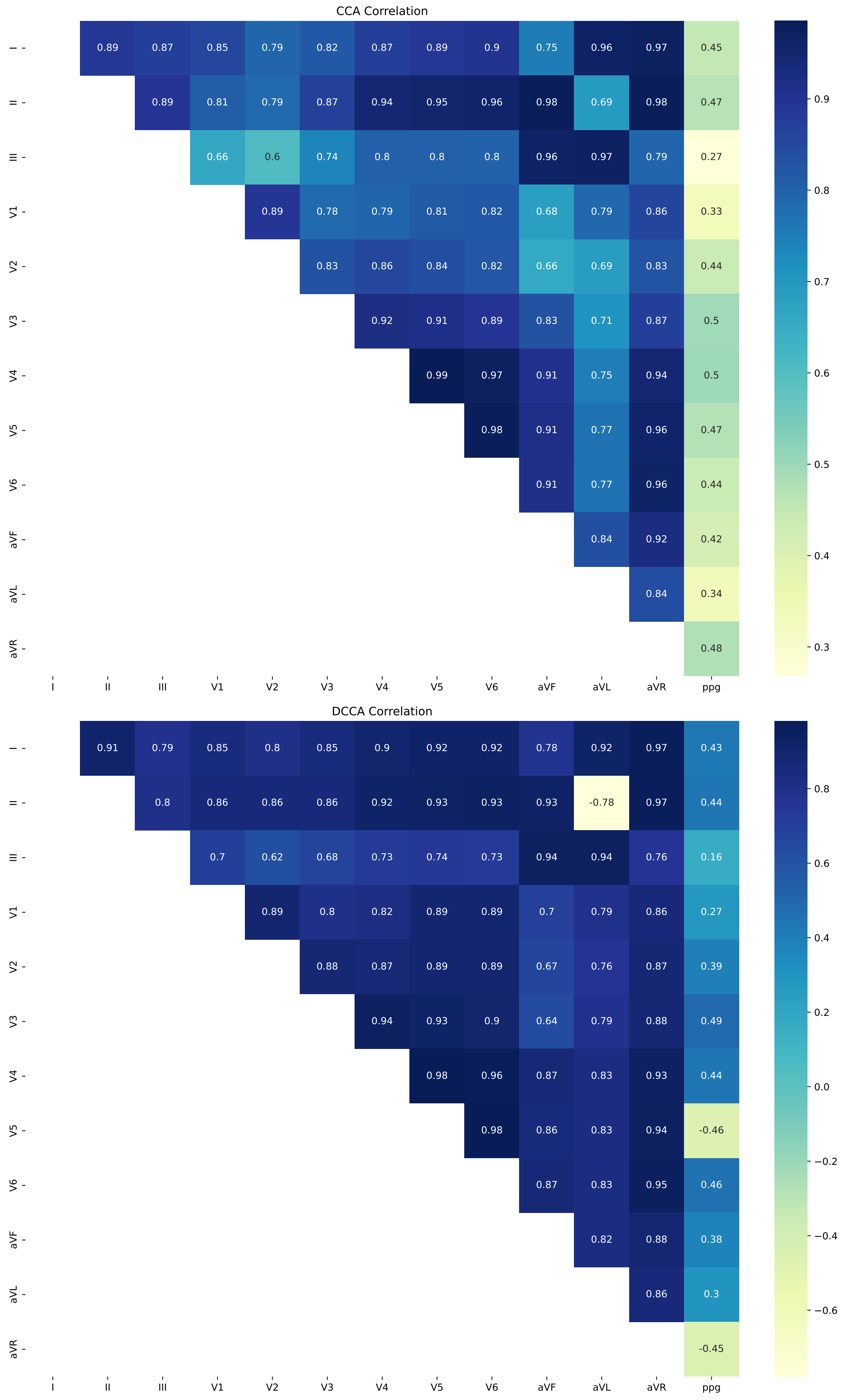

Figure S22: **CCA and DCCA between 12 ECG leads and PPG in UK Biobank.** We plotted the mean correlation obtained from CCA and DCCA. The standard error of correlation and numerical values see Table S8.

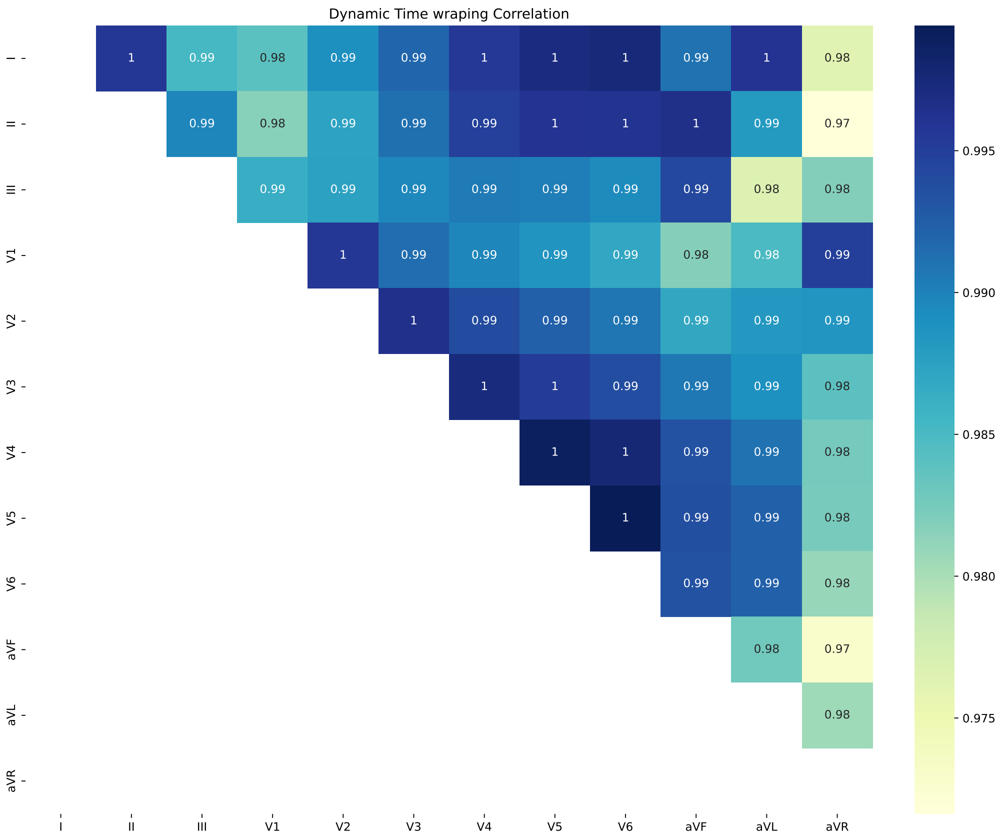

Figure S23: **Dynamic time warping between 12 ECG leads in UK Biobank.** We plotted the mean correlation obtained from dynamic time warping. We use the following formula: $r = 1 - D/2n$ where D is the computed distance, and n is the number of time points which we set 600 in the case of ECG 12 leads.

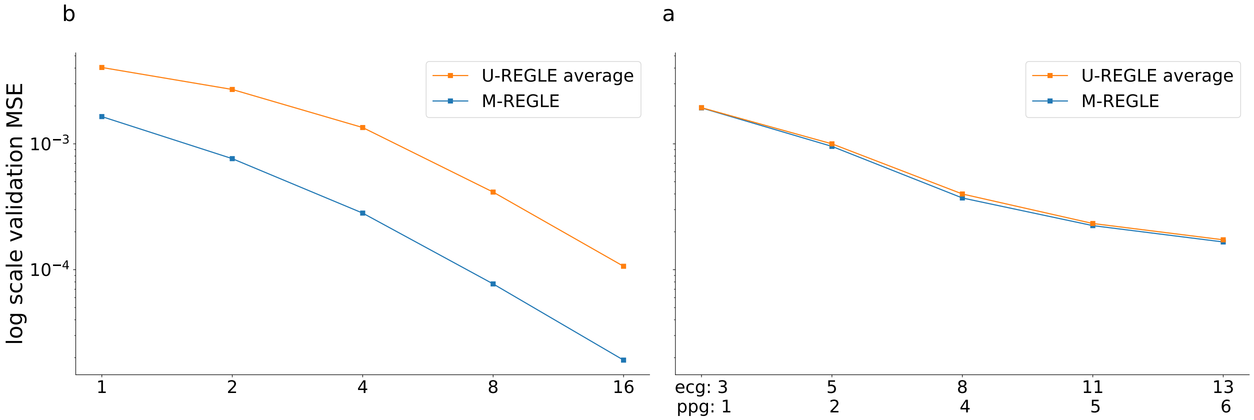

Figure S24: **Reconstruction errors of unimodal modal PCA and multimodal PCA across different PC numbers.** a) Plot of log scale validation reconstruction loss of unimodal modal PCA and multimodal PCA across different PC numbers on Lead I ECG + PPG data setting.b) Plot of log scale validation reconstruction loss of unimodal modal PCA and multimodal PCA across different PC numbers on 12-lead ECG data setting

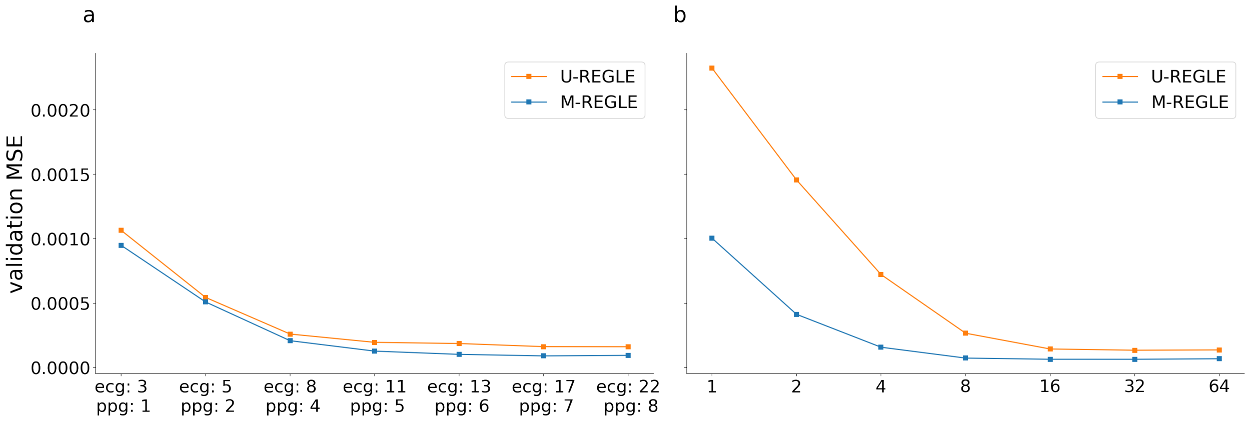

Figure S25: **Validation reconstruction losses of M-REGLE and U-REGLE across numbers of latent dimensions on Lead I ECG + PPG and 12-lead ECG datasets.** The differences are all significant. See Tables S9 and S13 for the values of reconstruction losses and standard errors.

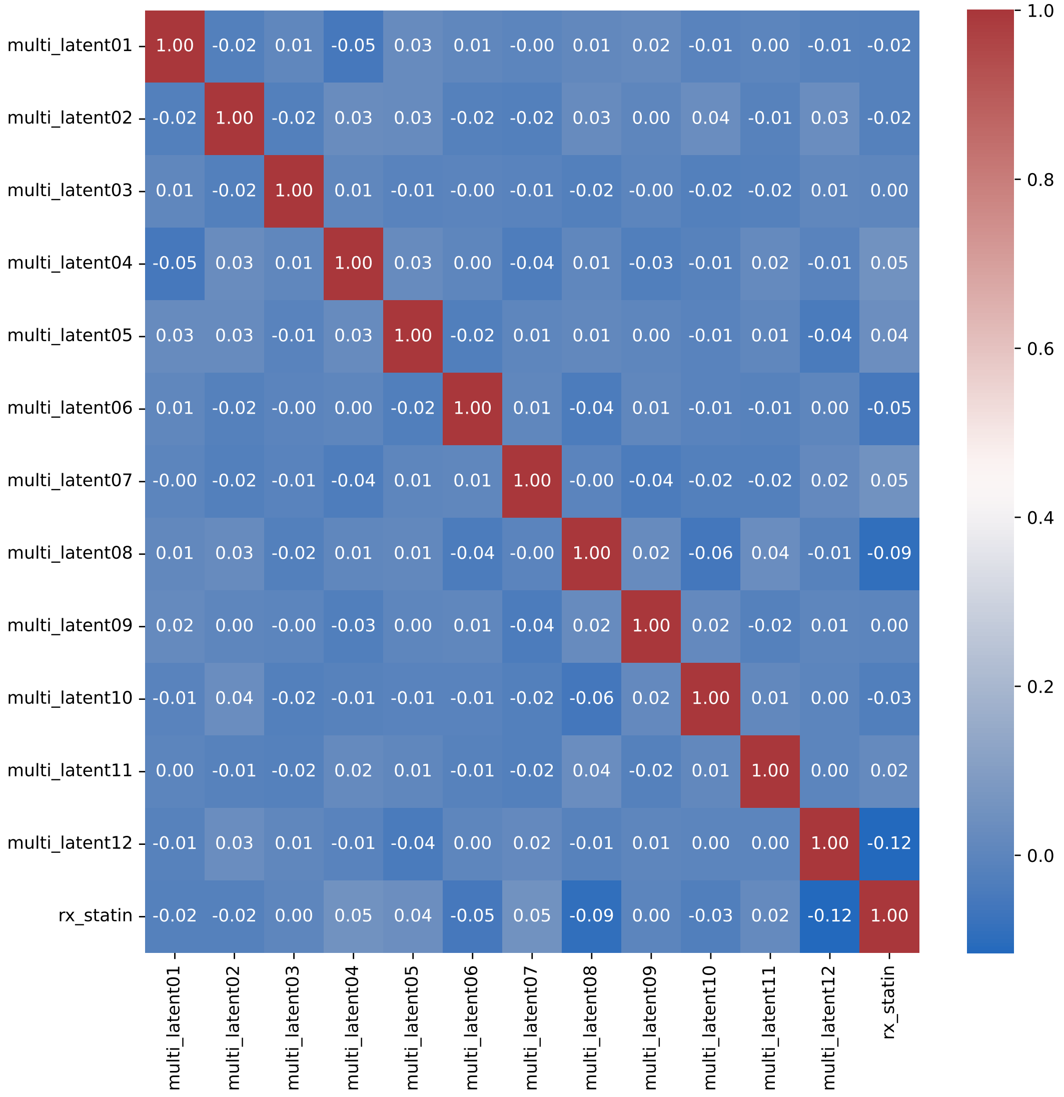

Figure S26: **The pairwise correlation between lead I ECG + PPG M-REGLE embeddings and Statin taken status in UK Biobank.** We consider any individual taken any of ’fluvastatin’, ’lovastatin’, ’rosuvastatin’, ’pitavastatin’, ’simvastatin’, ’atorvastatin’, ’pravastatin’ as taken of Statin.

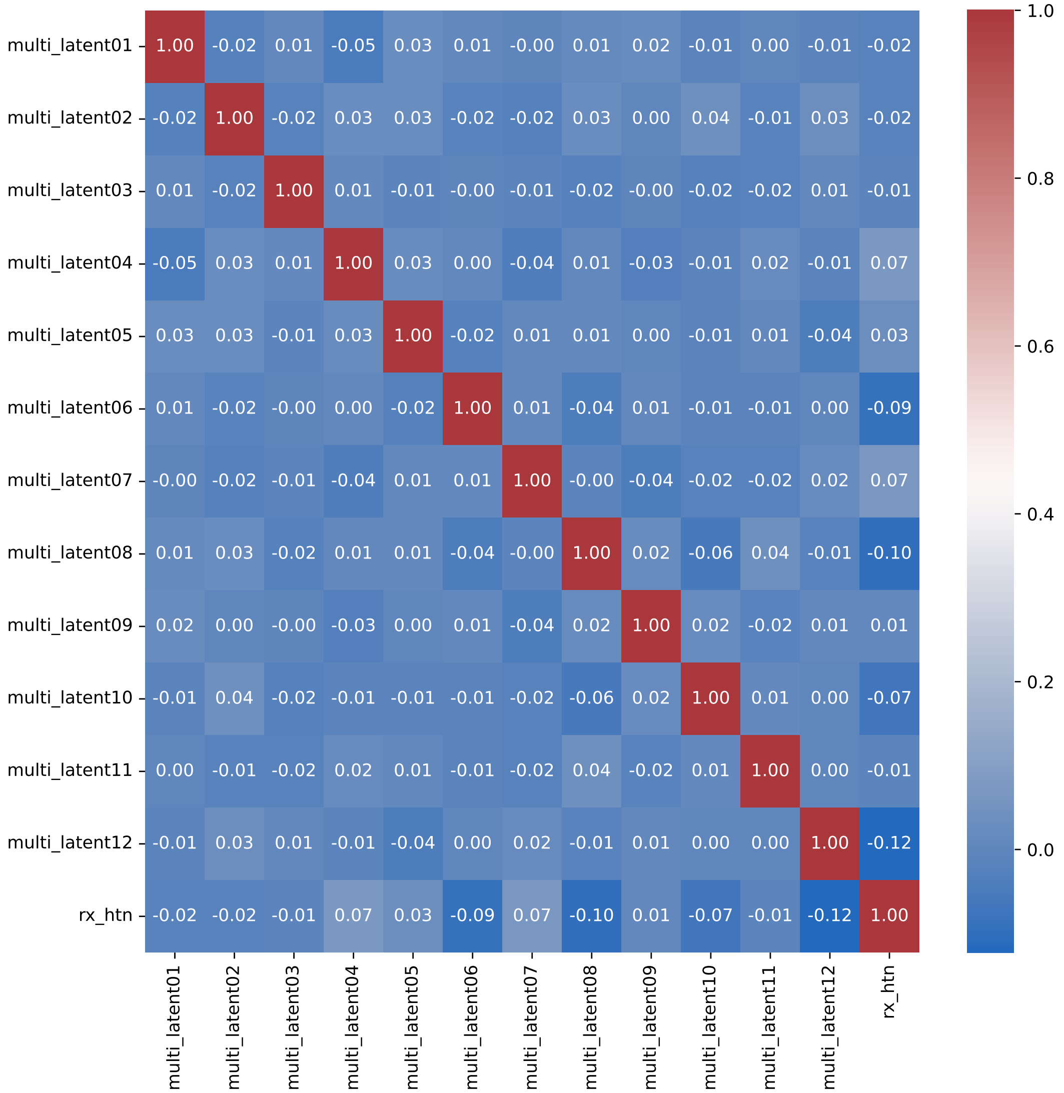

Figure S27: **The pairwise correlation between lead I ECG + PPG M-REGLE embeddings and hypertension (HTN) taken status in UK Biobank.**

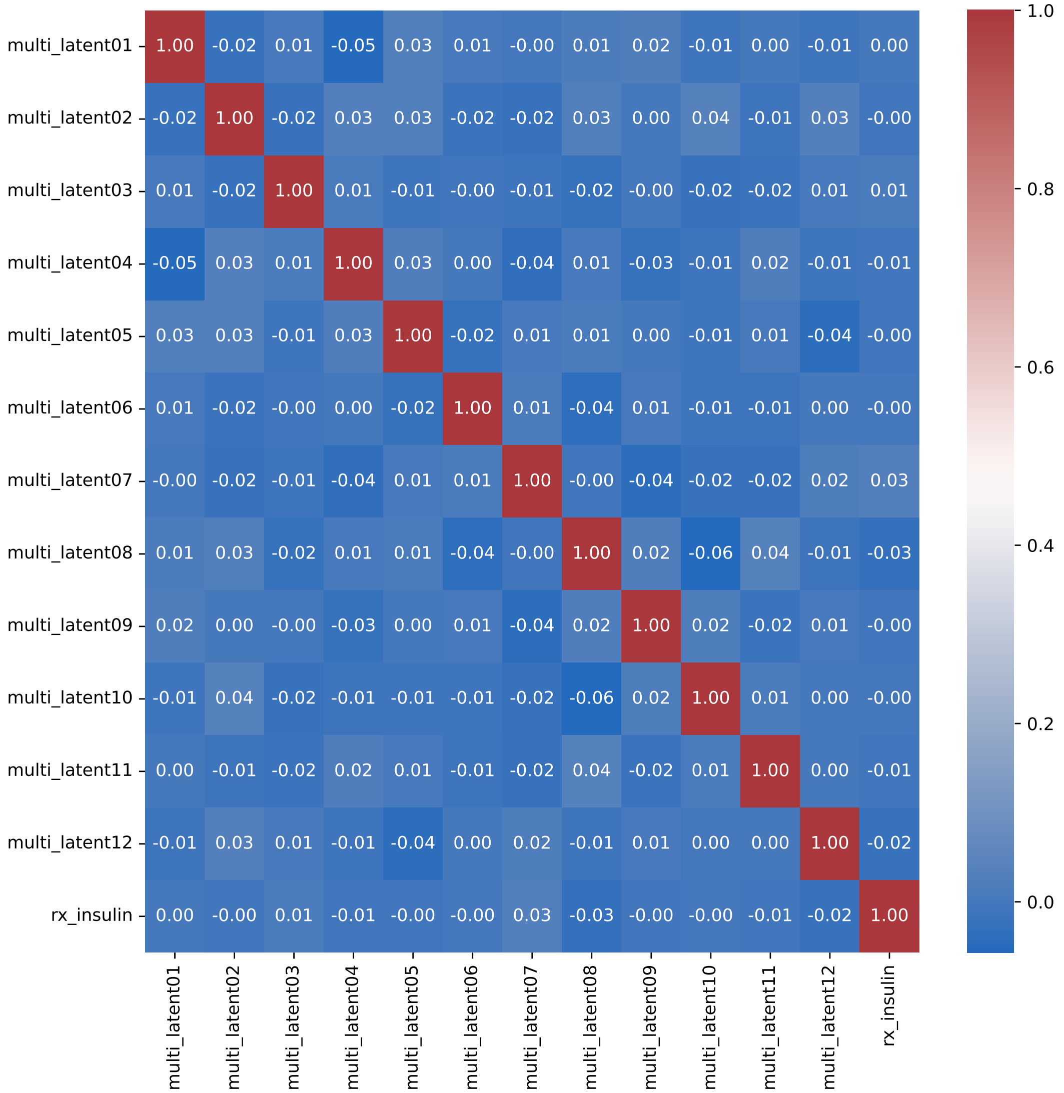

Figure S28: **The pairwise correlation between lead I ECG + PPG M-REGLE embeddings and Insulin taken status in UK Biobank.**

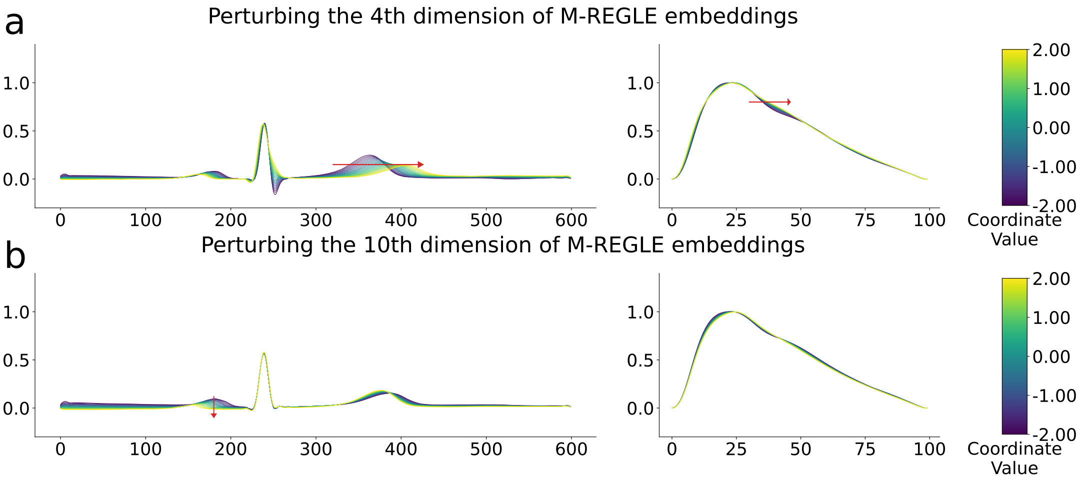

Figure S29: **The effect of perturbing one coordinate (4th or 10th) of M-REGLE embeddings from [-2, 2], while the values of the rest coordinates are 0s.** a) The impact of the 4th dimension. As the embedding transitions from -2 to 2, the decoded ECG (left) exhibits a prolonged QT interval, while the PPG (right) loses its notch. b) The impact of the 10th dimension. A shift from -2 to 2 along this dimension results in a progressive decrease in ECG amplitude.

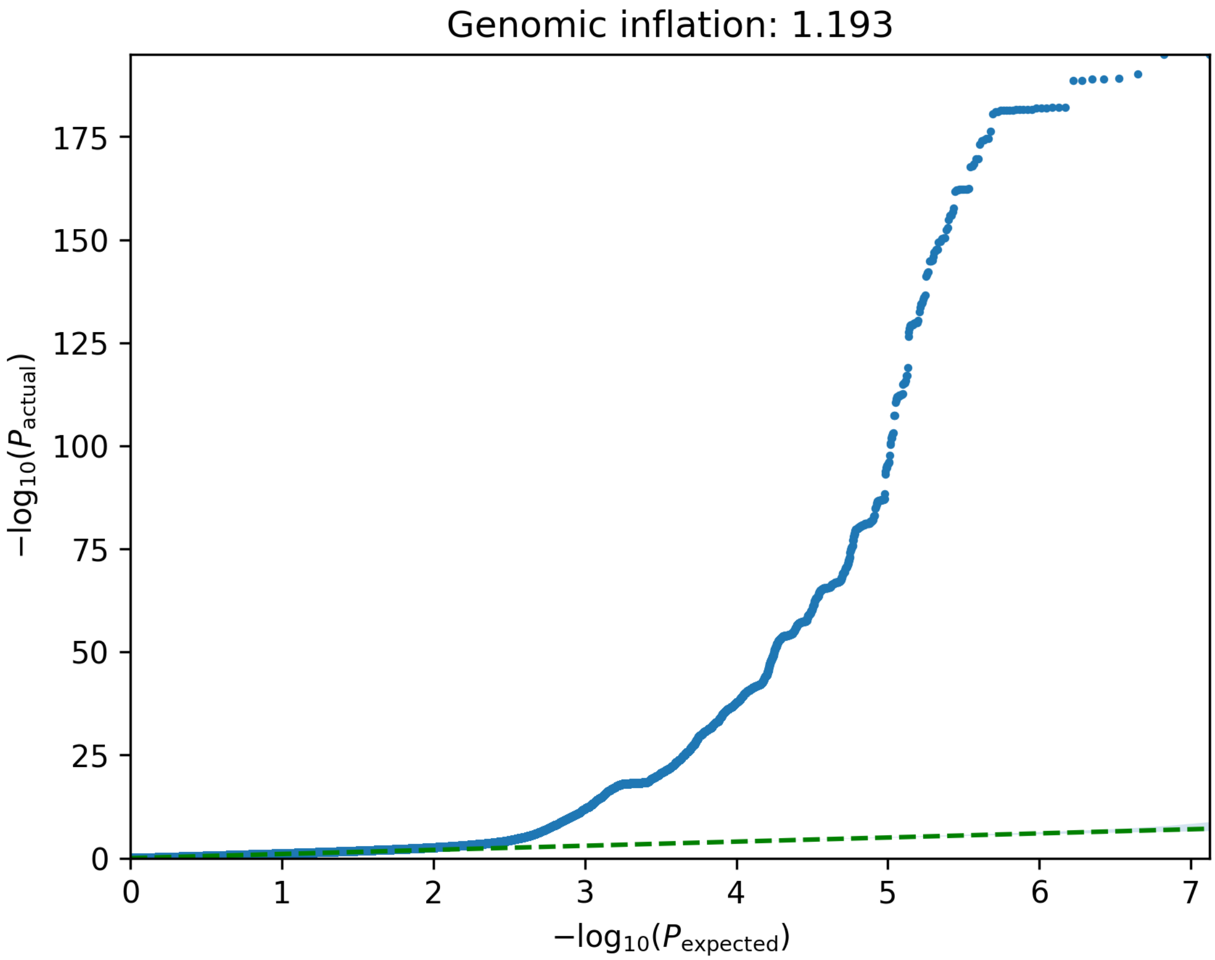

Figure S30: **QQ-plot M-REGLE combined on all 96 embeddings obtained from 12 lead ECG.**

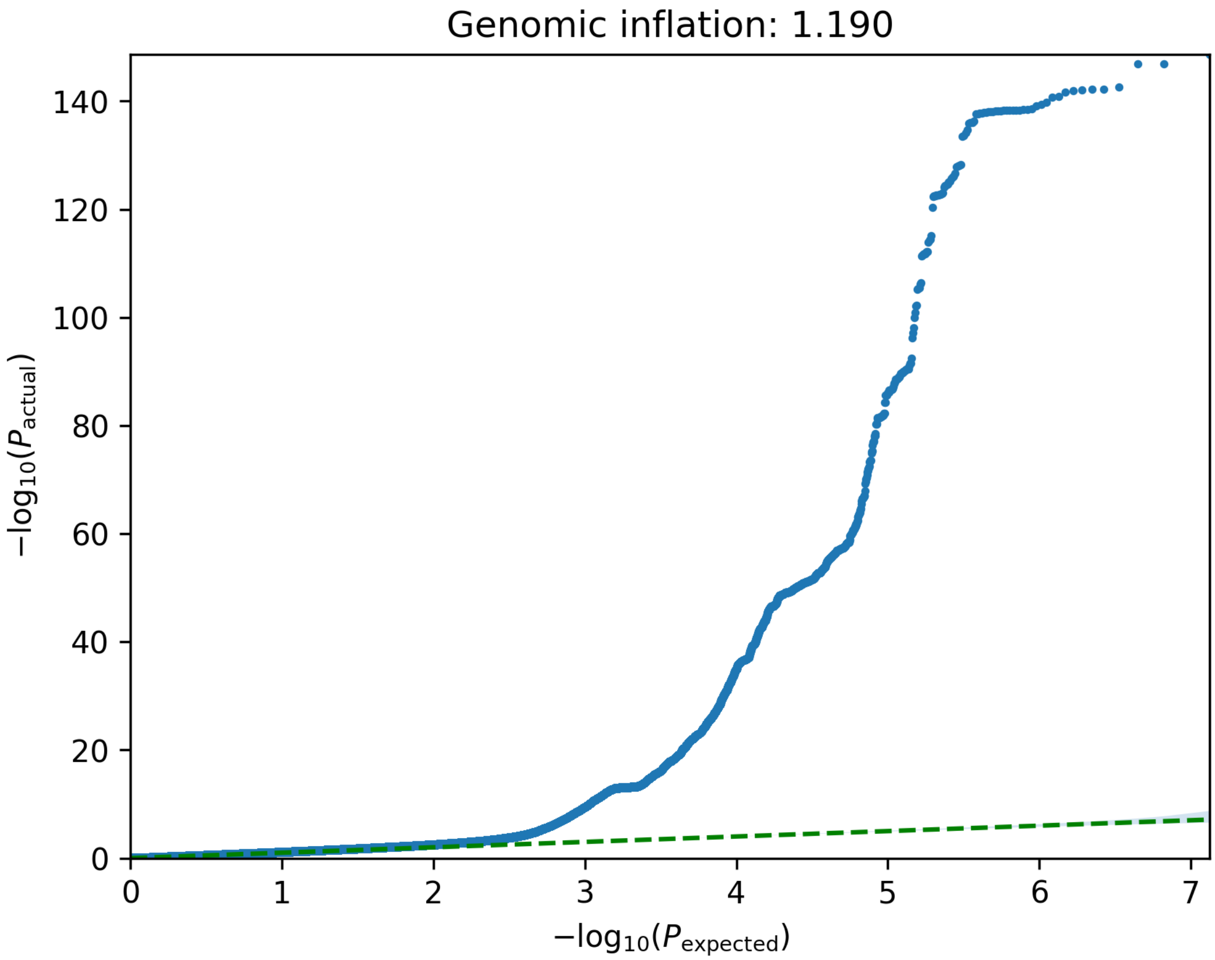

Figure S31: **QQ-plot U-REGLE combined on all 96 embeddings obtained from 12 lead ECG.**

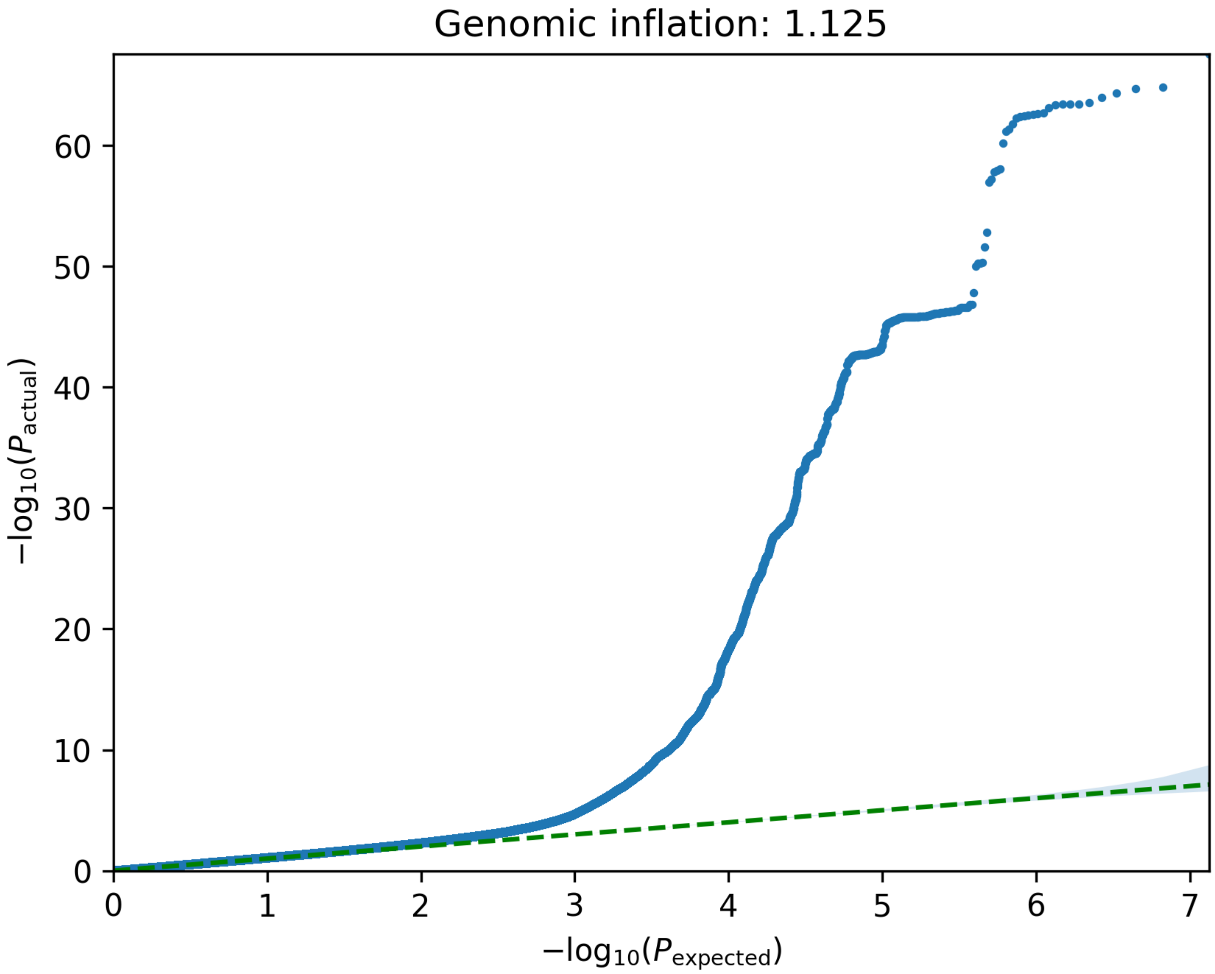

Figure S32: **QQ-plot M-REGLE combined on all 12 embedding obtained from lead I ECG and PPG.**

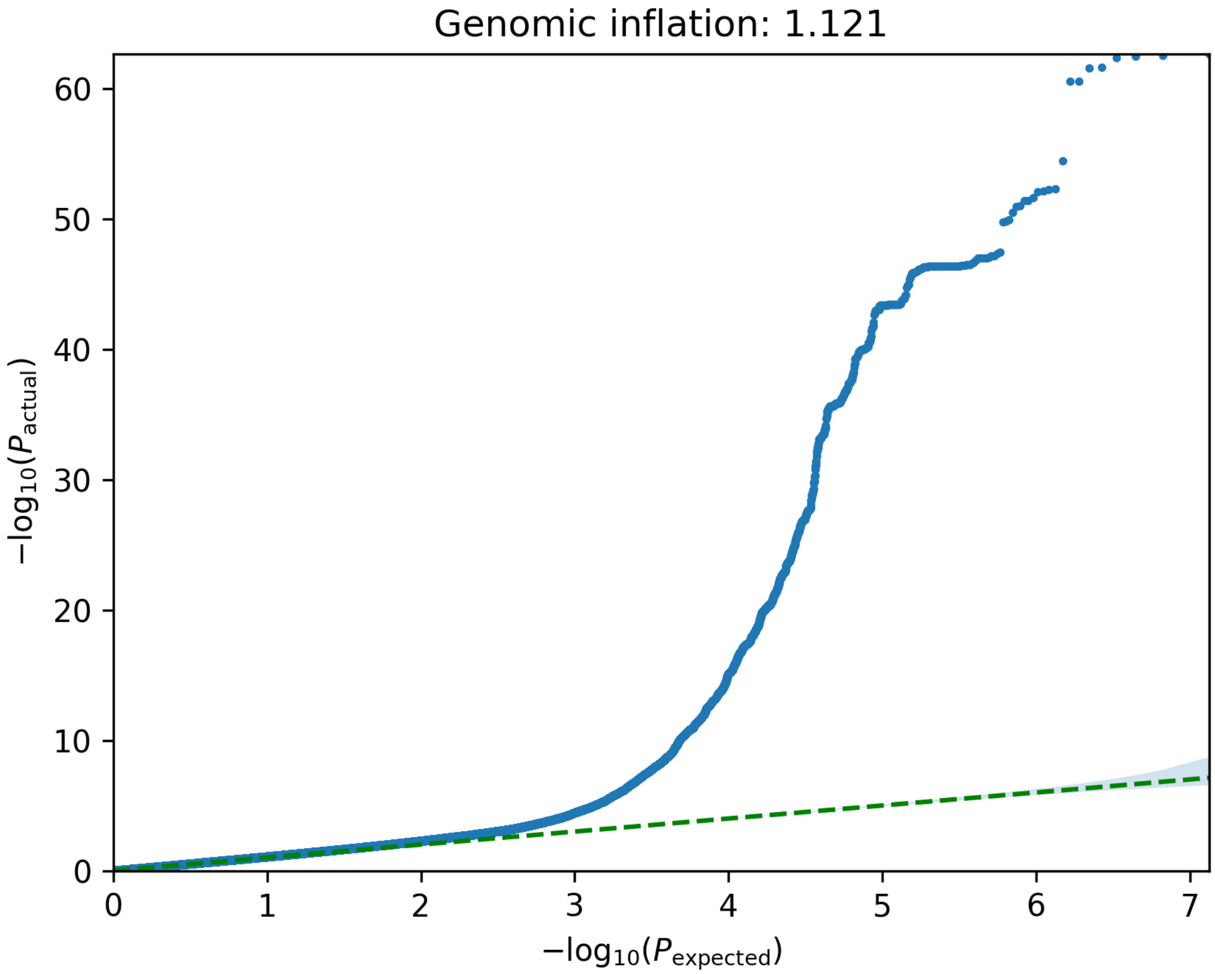

Figure S33: **QQ-plot U-REGLE combined on all 12 embedding obtained from lead I ECG and PPG.**

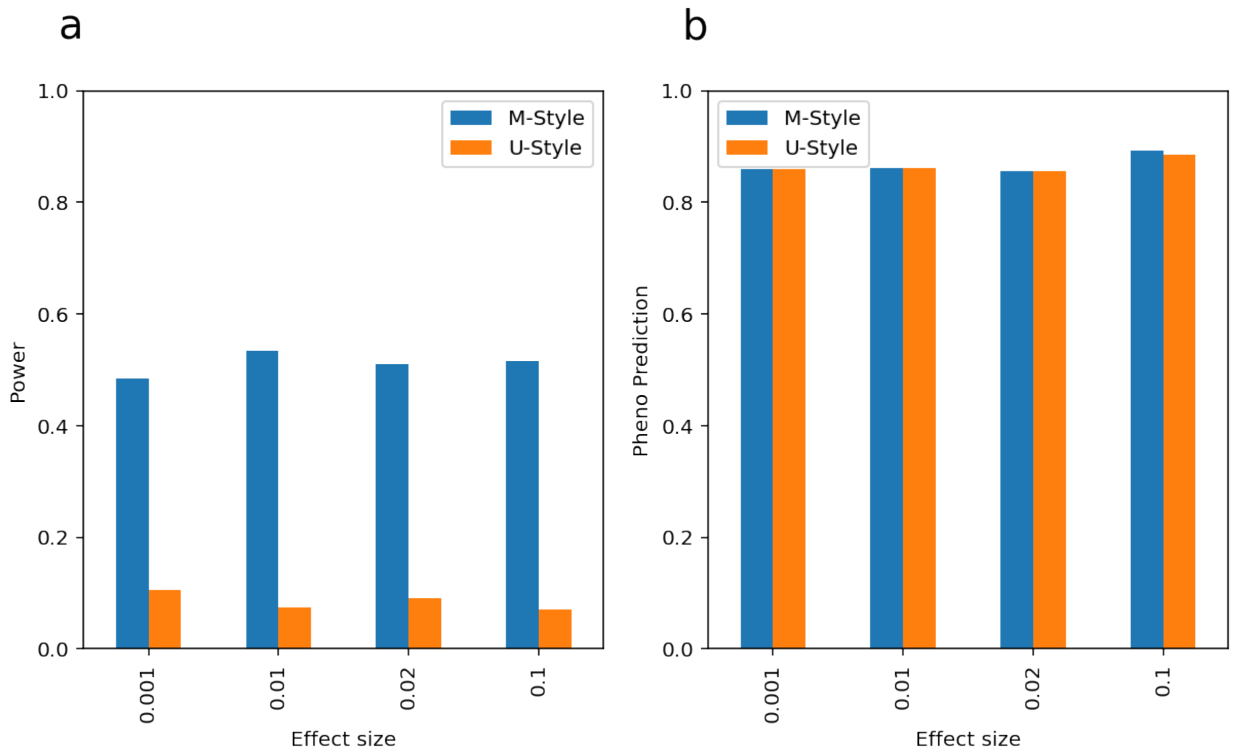

Figure S34: **Models with similar phenotype prediction can have different genetic analysis** We simulated two data modalities where in both modalities both genetic and non-genetic factor contribute to the data. However, the effect of non-genetic on data is larger than genetic effect. Panel (a) compute the power to detect the genetic factor and Panel (b) indicates the phenotype prediction from the learned embeddings. Effect size is the strength of genetic factor on the simulated phenotype of interest.

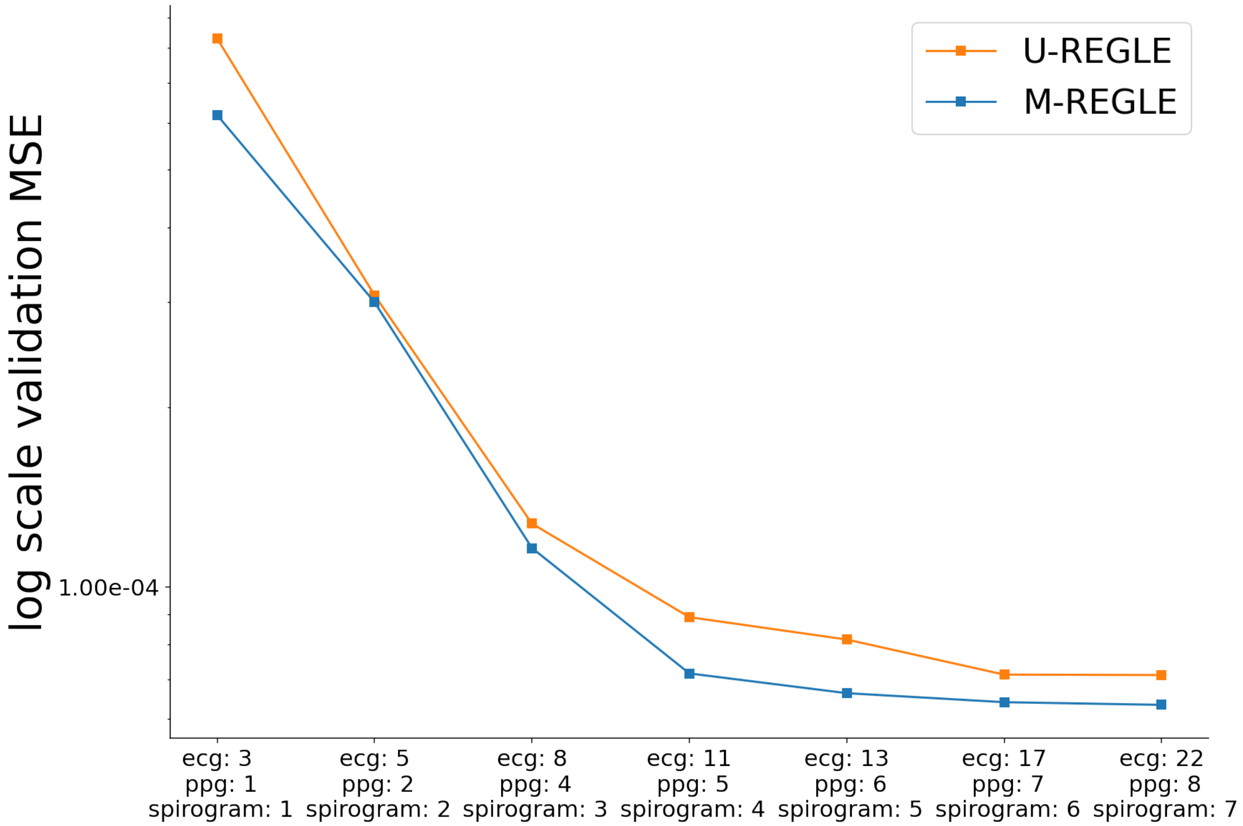

Figure S35: **Validation reconstruction losses in log-scale of M-REGLE and U-REGLE across numbers of latent dimensions on Lead I ECG, PPG and Spirogram dataset.** The differences are all significant. See Table S50 for the values of reconstruction losses and standard errors.

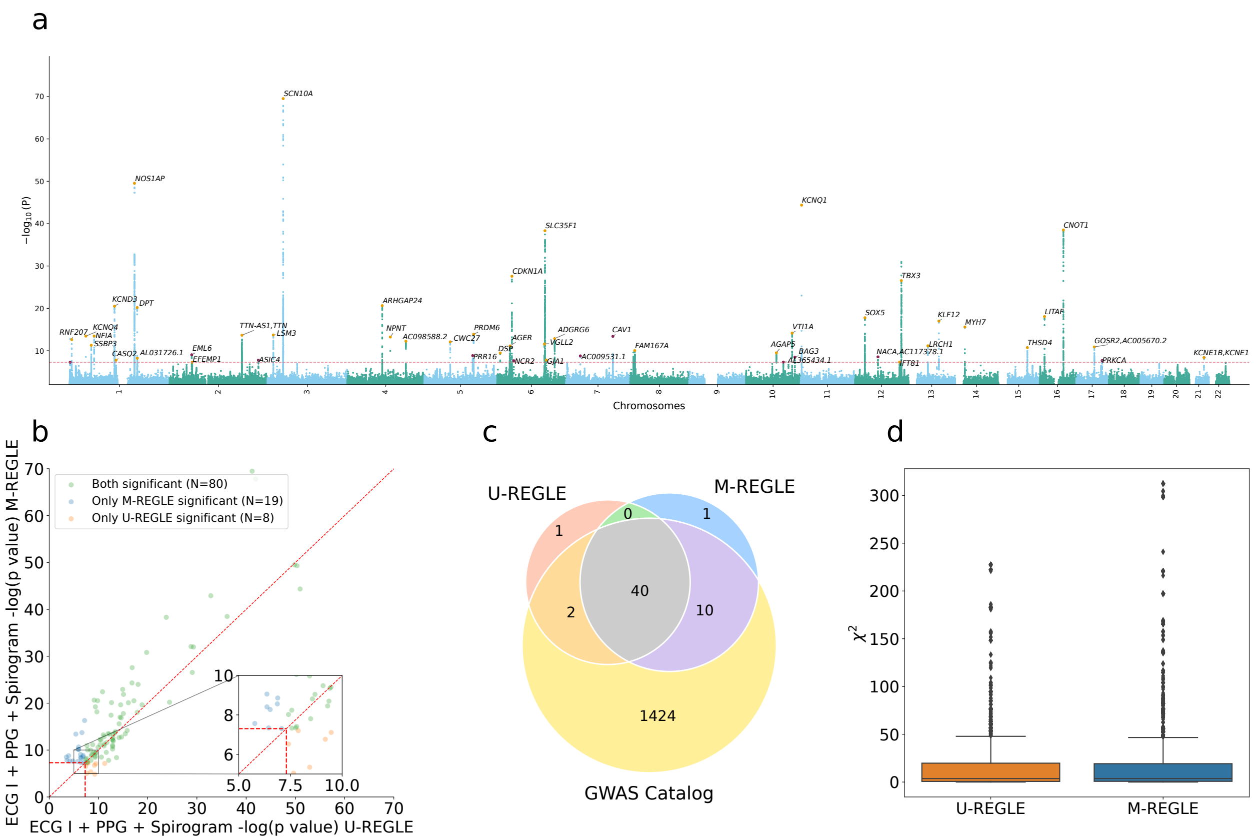

Figure S36: **M-REGLE on ECG lead I, PPG, and Spirograms increases genomic discovery**. a) Manhattan plot depicting M-REGLE GWAS p-values. Black gene names indicate the closest gene for each locus with $-\log_{10} p > 20$. Purple dots denote the GWS loci detected uniquely by M-REGLE. Orange dots indicate loci also identified in U-REGLE. b) Comparison of M-REGLE GWS variants-in-hits with U-REGLE. The X-axis is the $-\log$ p-value of Baseline. The Y-axis is the $-\log$ p-value of the M-REGLE. All p-values (a) and (b) are computed by summing the chi-square statistics for all 12 embeddings to perform a single joint chi-square test. The vertical and horizontal red lines indicate the GWS level. The diagonal red line indicates $y = x$. The orange dots indicate variants-in-hits that are significant for U-REGLE but not significant for our M-REGLE and green dots indicate variants-in-hits that are significant for our M-REGLE but not significant for Baseline. c) A 3 way Venn diagram of the GWAS catalog loci, loci discovered by M-REGLE and loci discovered by U-REGLE. d) Comparison of the chi-square statistics for all known significant variants in GWAS catalog for both U-REGLE and M-REGLE. The difference is statistically significant.

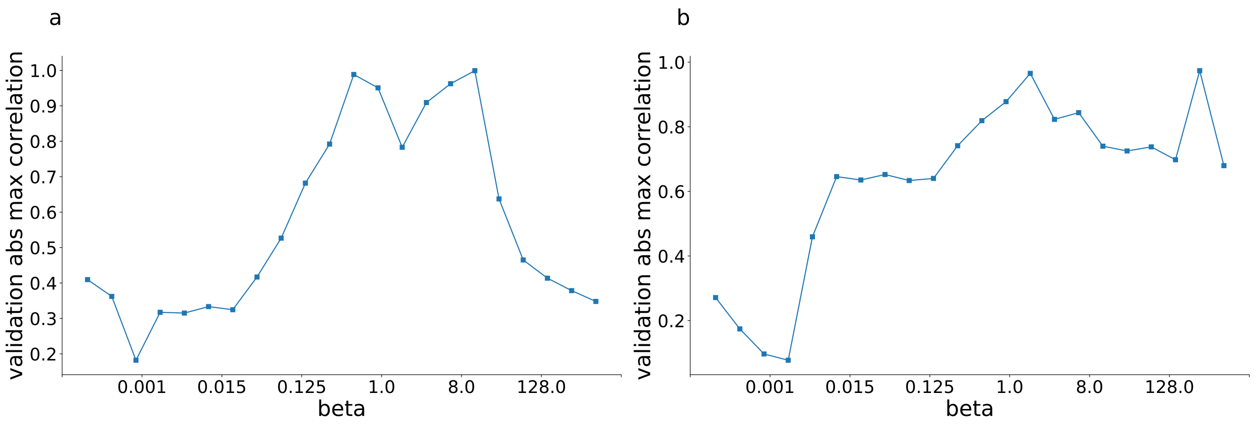

Figure S37: **Plot of maximum absolute non-diagonal correlation coefficients of M-REGLE lower embeddings when training with different beta values.** a) Maximum absolute non-diagonal correlation coefficients VS beta plot of 12-lead ECG, b) Maximum absolute non-diagonal correlation coefficients VS beta plot of ECG+PPG.

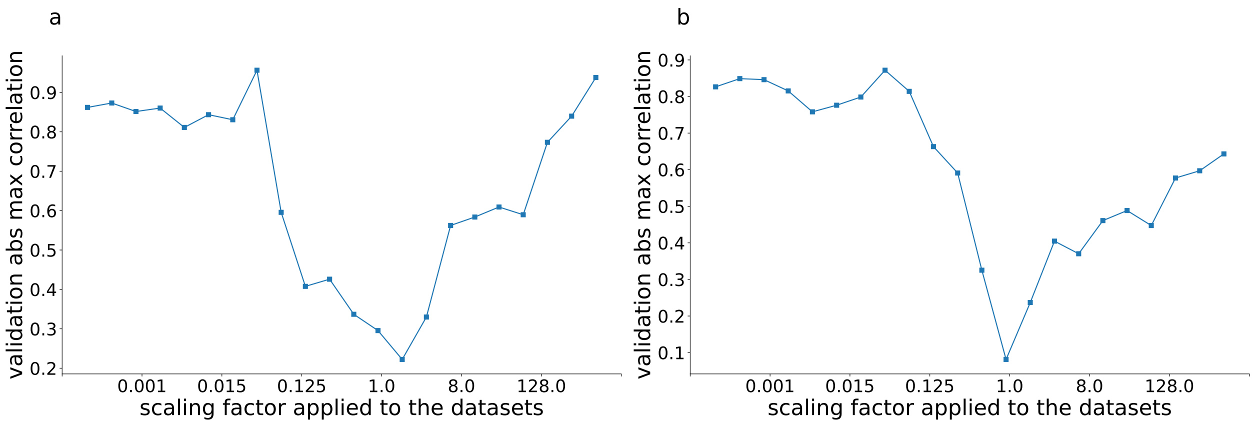

Figure S38: **Plot of maximum absolute non-diagonal correlation coefficients of M-REGLE lower embeddings when different scaling factors applied to the datasets.** a) Maximum absolute non-diagonal correlation coefficients VS scaling factor plot of 12-lead ECG, b) Maximum absolute non-diagonal correlation coefficients VS scaling factor plot of ECG+PPG.

Supplemental Tables

ρ	N	Naive	PCA	MANOVA
0	5000	1.05 (0.14)	1.06 (0.14)	1.06 (0.14)
	10000	1.02 (0.20)	1.02 (0.20)	1.02 (0.20)
	50000	1.07 (0.15)	1.07 (0.15)	1.07 (0.15)
	100000	1.07 (0.15)	1.07 (0.14)	1.07 (0.14)
	200000	1.08 (0.14)	1.08 (0.14)	1.08 (0.14)
0.2	5000	1.09 (0.17)	1.12 (0.16)	1.12 (0.16)
	10000	1.15 (0.22)	1.02 (0.15)	1.02 (0.15)
	50000	1.09 (0.17)	0.98 (0.13)	0.98 (0.13)
	100000	1.11 (0.16)	0.97 (0.12)	0.97 (0.12)
	200000	0.83 (0.15)	0.81 (0.11)	0.81 (0.11)
0.3	5000	1.24 (0.18)	0.90 (0.12)	0.90 (0.12)
	10000	1.51 (0.39)	0.94 (0.15)	0.94 (0.15)
	50000	1.11 (0.19)	0.96 (0.11)	0.96 (0.11)
	100000	1.43 (0.25)	0.84 (0.10)	0.84 (0.10)
	200000	1.26 (0.32)	0.90 (0.12)	0.90 (0.12)

Table S1: **Type I error comparison.** We compared three models: (1) a naive model where we tested each factor independently and then summed the chi-square statistics; (2) a PCA model where we performed PCA on the factors and then summed the chi-square statistics; and (3) multivariate analysis of variance (MANOVA).

ρ	N	Naive	PCA	MANOVA
0	5000	1.15 (0.13)	1.14 (0.13)	1.14 (0.13)
	10000	1.43 (0.16)	1.43 (0.16)	1.43 (0.16)
	50000	2.96 (0.28)	2.95 (0.28)	2.95 (0.28)
	100000	4.76 (0.43)	4.76 (0.43)	4.76 (0.43)
	200000	12.73 (0.70)	12.73 (0.70)	12.73 (0.70)
0.2	5000	1.20 (0.19)	1.14 (0.16)	1.14 (0.16)
	10000	1.92 (0.23)	1.73 (0.18)	1.73 (0.18)
	50000	2.41 (0.25)	2.74 (0.26)	2.74 (0.26)
	100000	5.17 (0.49)	6.16 (0.52)	6.16 (0.52)
	200000	10.54 (0.62)	13.02 (0.70)	13.02 (0.70)
0.3	5000	1.32 (0.231)	1.24 (0.15)	1.24 (0.151)
	10000	1.57 (0.231)	1.59 (0.17)	1.59 (0.171)
	50000	3.21 (0.401)	4.29 (0.42)	4.29 (0.421)
	100000	5.10 (0.441)	6.66 (0.45)	6.66 (0.451)
	200000	11.58 (0.731)	16.34 (0.91)	16.35 (0.911)

Table S2: **Power comparison.** We compared three models: (1) a naive model where we tested each factor independently and then summed the chi-square statistics; (2) a PCA model where we performed PCA on the factors and then summed the chi-square statistics; and (3) multivariate analysis of variance (MANOVA).

See the attached Excel table.

Table S3: **Overview of hyperparameter sweep ranges for M-REGLE and U-REGLE VAE models.**

See the attached Excel table.

Table S4: **Overview of final hyperparameters used in M-REGLE and U-REGLE VAE models.**

Latent dimensions	Combine modalities into 1 channel	Combine modalities into 2 channels
4	9.49e-04 ± 1.98e-06	1.174-03 ± 1.2e-05
7	5.09e-04 ± 8.86e-07	6.07e-04 ± 7e-06
12	2.08e-04 ± 3.82e-07	2.56e-04 ± 3e-06
16	1.27e-04 ± 2.60e-07	1.55e-04 ± 2e-06
19	1.02e-04 ± 1.90e-07	1.14e-04 ± 2e-07

Table S5: **Comparison of the MSE of different VAE model architectures across different latent dimension numbers on lead I ECG + PPG dataset.** For learning M-REGLE representations on Lead I ECG + PPG modalities, besides the model architecture we used in the paper, where we combined the ECG waveform and PPG waveform by concatenating them into one channel, we also tried the model architecture of using ECG and PPG as 2 channels. We first upsampled PPGs, to match the length of ECGs, and they can be fed into the input of 2 channels. We trained the VAE model to reconstruct ECG and upsampled PPG. As the table shows, this 2-channel model does not perform as well as the model we used in the paper. This result is expected. ECG and PPG of the same individual are not collected synchronously, but putting them into 2 input channels may indicate same time stamps at each pair of aligned data points, which is not true. Therefore, this can hurt the model's performance.

12 lead ECG	lead I ECG + PPG	lead I ECG + PPG + Spirograms
Electrocardio	Electrocardio	Electrocardio
ECG	ECG	ECG
EKG	ECK	ECK
PR interval	PR interval	PR interval
Ventricular rate	Ventricular rate	Ventricular rate
PP interval	PP interval	PP interval
PQ interval	PQ interval	PQ interval
QRS duration	QRS duration	QRS duration
QT interval	QT interval	QT interval
RR interval	RR interval	RR interval
P axis	P axis	P axis
R axis	R axis	R axis
T axis	T axis	T axis
	Arterial stiffness	Arterial stiffness
	Pulse wave	Pulse wave
	Pulse waveform	Pulse waveform
	Pulse-wave	Pulse-wave
	Notch position	Notch position
		COPD
		Asthma
		FEV1
		FVC
		Lung function
		PEF
		FEV/FVC ratio

Table S6: **Regular expression key words used for search in GWAS catalog.**

	Train	Eval	Test
Myocardial Infarction	0.0593	0.0599	0.0585
Afib	0.0722	0.0729	0.0709
T2D	0.0836	0.0825	0.0828
Cardiovascular Disease	0.4149	0.4116	0.4150
Hypertension	0.3941	0.3909	0.3942
Ever smoked	0.6031	0.6013	0.6008

Table S7: **UK Biobank binary phenotypes prevalence for different data splits.**

See the attached Excel table.

Table S8: **CCA and DCCA between 12 ECG leads and PPG in UK Biobank.**

Latent Dim. per channel	Multimodal	Unimodal
1	1.00e-03 ± 6.77e-07	2.32e-03 ± 1.43e-06
2	4.13e-04 ± 2.55e-07	1.46e-03 ± 8.46e-07
4	1.58e-04 ± 1.68e-07	7.22e-04 ± 3.26e-07
8	7.32e-05 ± 8.40e-08	2.67e-04 ± 1.62e-07
16	6.41e-05 ± 7.18e-08	1.46e-04 ± 9.01e-08
32	6.37e-05 ± 1.21e-06	1.36e-04 ± 1.00e-06
64	6.84e-05 ± 1.20e-06	1.40e-04 ± 1.06e-06

Table S9: **Comparison of multimodal VAE MSE and an average of all unimodal modal VAE MSEs across different latent dimension numbers on 12 lead ECG dataset.** Latent Dim stands for latent dimension.

Latent Dim. per channel	Multimodal	Unimodal
1	1.60e-03 ± 1.26e-06	3.88e-03 ± 2.60e-06
2	7.63e-04 ± 6.12e-07	2.62e-03 ± 1.80e-06
4	2.88e-04 ± 2.74e-07	1.33e-03 ± 8.02e-07
8	8.50e-05 ± 1.15e-07	4.38e-04 ± 3.44e-07
16	2.46e-05 ± 4.42e-08	1.17e-04 ± 1.49e-07

Table S10: **Comparison of multimodal PCA MSE and an average of all unimodal modal PCA MSEs across different PC numbers on 12 lead ECG dataset.**

Latent Dim. per channel	Multimodal	Unimodal
1	1.03e-03	2.69e-03
2	4.32e-04	1.71e-03
4	1.64e-04	8.08e-04
8	7.71e-05	2.45e-04
16	4.70e-05	7.97e-05

Table S11: **Comparison of multimodal CAE MSE and an average of all unimodal modal CAE MSEs across different latent dimension numbers on 12 lead ECG dataset.** Latent Dim stands for latent dimension.

# ECG Latent Dim.	ECG variance explained	# PPG Latent Dim.	PPG variance explained
3	0.6807	1	0.6728
5	0.8353	2	0.8370
8	0.9311	4	0.9496
11	0.9616	5	0.9660
13	0.9716	6	0.9763
17	0.9834	7	0.9848
22	0.9902	8	0.9912

Table S12: **Latent dimension number and variance explained through PCA for Lead I ECG and PPG.**

Variance explained	Multimodal	Unimodal
70%	9.49e-04 ± 1.98e-06	1.07e-03 ± 2.26e-06
83%	5.09e-04 ± 8.86e-07	5.44e-04 ± 9.42e-07
93%-94%	2.08e-04 ± 3.82e-07	2.60e-04 ± 4.46e-07
96%	1.27e-04 ± 2.60e-07	1.95e-04 ± 3.36e-07
97%	1.02e-04 ± 1.90e-07	1.86e-04 ± 3.01e-07
98%	9.05e-05 ± 1.15e-06	1.63e-04 ± 1.51e-06
99%	9.39e-05 ± 1.23e-06	1.64e-04 ± 1.50e-06

Table S13: **Comparison of multimodal VAE MSE and an average of all unimodal modal VAE MSEs across different latent dimension numbers on lead I ECG + PPG dataset.**

Variance explained	Multimodal	Unimodal
70%	1.93e-03 ± 4.64e-06	1.94e-03 ± 4.65e-06
83%	9.54e-04 ± 2.78e-06	1.00e-03 ± 2.37e-06
93%-94%	3.71e-04 ± 1.13e-06	3.99e-04 ± 1.29e-06
96%	2.24e-04 ± 8.68e-07	2.33e-04 ± 8.66e-07
97%	1.66e-04 ± 7.55e-07	1.73e-04 ± 7.84e-07

Table S14: **Comparison of multimodal PCA MSE and an average of all unimodal modal PCA MSEs across different latent dimension numbers on lead I ECG + PPG dataset.**

Variance explained	Multimodal	Unimodal
70%	9.72e-04	9.43e-04
83%	5.48e-04	4.99e-04
93%-94%	1.90e-04	1.93e-04
96%	1.16e-04	1.14e-04
97%	8.61e-05	8.47e-05

Table S15: **Comparison of multimodal CAE MSE and an average of all unimodal modal CAE MSEs across different latent dimension numbers on lead I ECG + PPG dataset.**

See the attached Excel table.

Table S16: **M-REGLE phenotypic correlation with UKB phenotypes.**

Pheno	PRS Sample		ECG+PPG		12 lead ECG	
	Control	Cases	Control	Cases	Control	Cases
Afib	430076	33513	33536	1415	38993	1651
All cause mortality	444096	42840	35505	592	41536	669
Angina	452379	34772	34381	1716	40246	1959
Cardiovascular disease	285273	201790	23511	12585	27811	14393
Coronary artery disease	439209	47942	33974	2123	39749	2456
Diabetes type 2	424934	38655	33247	1704	38705	1939
Hypertension	281821	182847	23590	11389	27712	12985
Myocardial_infarction	458149	28914	34774	1322	40672	1532
Stroke	424003	15903	30977	679	36118	781

Table S17: **Sample size of UK Biobank phenotypes.**

See the attached Excel table.

Table S18: **Phenotype prediction metrics by 12-lead ECG M-REGLE & U-REGLE embeddings.**

See the attached Excel table.

Table S19: **Paired test of phenotype prediction metrics by 12-lead ECG M-REGLE & U-REGLE embeddings.**

See the attached Excel table.

Table S20: **Phenotype prediction metrics by ECG lead I + PPG M-REGLE & U-REGLE embeddings.**

See the attached Excel table.

Table S21: **Paired test of phenotype prediction metrics by ECG lead I + PPG M-REGLE & U-REGLE embeddings.**

Feature	Num. Features	UKB Field Codes
Age	1	21003
Sex	1	31
BMI	1	21001
Smoking	1	20160
HDL	1	30760
LDL	1	30780
Obesity	1	130792 and ICD-10 E66.*
SBP	1	4080
DBP	1	4079
Metabolic panel (lipid)	251	Category 220
		`https://biobank.ndph.ox.ac.uk/showcase/label.cgi?id=220`
Activity	4	22040, 22033, 904, 22038
Socioeconomic factors	9	738, 26416, 26414, 26412, 26413, 26415, 26411, 26410, 26417
Socioeconomic factors	16	680, 728, 709, 816, 4674, 6142 (3 employment)
Self-reported		6143 (4 transportation), 6138 (4 education levels)

Table S22: **Predictor of known cardiovascular features in UKB.** We consider 3 employment: employed, volunteer, student. We considered 4 education level: college, freshman, high school, and professional qualifications e.g.,: nursing, teaching. We considered 4 mean of transportation: car, bike, walking, and public transport.

Pheno	AUROC	AUPRC	Top 1% prevalence	Top 10% prevalence
Hypertension	0.81 (0.80, 0.82)	0.65 (0.63, 0.67)	0.74 (0.71, 0.77)	0.87 (0.76, 0.96)
Diabetes type 2	0.83 (0.81, 0.85)	0.38 (0.33, 0.43)	0.25 (0.22, 0.28)	0.77 (0.67, 0.86)
Cardiovascular disease	0.80 (0.79, 0.81)	0.66 (0.64, 0.68)	0.77 (0.75, 0.80)	0.87 (0.80, 0.93)
Myocardial infarction	0.74 (0.71, 0.76)	0.13 (0.10, 0.16)	0.15 (0.12, 0.17)	0.22 (0.13, 0.31)
Afib	0.72 (0.70, 0.74)	0.11 (0.09, 0.13)	0.12 (0.10, 0.14)	0.16 (0.10, 0.24)
Coronary artery disease	0.78 (0.76, 0.79)	0.22 (0.18, 0.25)	0.23 (0.21, 0.26)	0.40 (0.28, 0.50)
Stroke	0.73 (0.70, 0.77)	0.06 (0.04, 0.09)	0.06 (0.04, 0.08)	0.10 (0.04, 0.18)
All cause mortality	0.75 (0.72, 0.79)	0.04 (0.03, 0.05)	0.05 (0.03, 0.06)	0.06 (0.02, 0.11)

Table S23: **Prediction of known cardiovascular features (Supplementary Table S22) in UKB.**

Pheno	AUROC	AUPRC	Top 1% prevalence	Top 10% prevalence
Hypertension	0.82 (0.81, 0.83)	0.66 (0.64, 0.68)	0.75 (0.73, 0.78)	0.89 (0.82, 0.96)
Diabetes type 2	0.84 (0.82, 0.86)	0.39 (0.35, 0.44)	0.25 (0.22, 0.28)	0.79 (0.70, 0.88)
Cardiovascular disease	0.81 (0.80, 0.81)	0.67 (0.66, 0.69)	0.78 (0.76, 0.80)	0.91 (0.85, 0.96)
Myocardial infarction	0.75 (0.71, 0.77)	0.15 (0.12, 0.19)	0.15 (0.13, 0.17)	0.30 (0.20, 0.40)
Afib	0.75 (0.73, 0.78)	0.24 (0.19, 0.29)	0.18 (0.15, 0.21)	0.53 (0.43, 0.63)
Coronary artery disease	0.79 (0.77, 0.81)	0.26 (0.22, 0.29)	0.25 (0.22, 0.29)	0.48 (0.37, 0.57)
Stroke	0.74 (0.71, 0.77)	0.07 (0.04, 0.09)	0.06 (0.04, 0.08)	0.09 (0.04, 0.15)
All cause mortality	0.76 (0.73, 0.80)	0.05 (0.03, 0.07)	0.04 (0.03, 0.05)	0.09 (0.04, 0.15)

Table S24: **Prediction of M-REGLE embeddings from 12-lead ECG and known cardiovascular features (Supplementary Table S22) in UKB.**

Pheno	AUROC	AUPRC	Top 1% prevalence	Top 10% prevalence
Hypertension	0.82 (0.81, 0.83)	0.66 (0.64, 0.68)	0.75 (0.73, 0.78)	0.91 (0.84, 0.97)
Diabetes type 2	0.84 (0.82, 0.86)	0.39 (0.35, 0.44)	0.26 (0.22, 0.29)	0.80 (0.70, 0.88)
Cardiovascular disease	0.81 (0.80, 0.81)	0.67 (0.66, 0.69)	0.78 (0.76, 0.81)	0.91 (0.85, 0.96)
Myocardial infarction	0.74 (0.71, 0.78)	0.15 (0.12, 0.19)	0.15 (0.13, 0.17)	0.32 (0.22, 0.38)
Afib	0.75 (0.72, 0.78)	0.24 (0.19, 0.29)	0.19 (0.16, 0.21)	0.56 (0.43, 0.67)
Coronary artery disease	0.79 (0.77, 0.81)	0.25 (0.21, 0.28)	0.24 (0.21, 0.27)	0.50 (0.38, 0.60)
Stroke	0.75 (0.71, 0.78)	0.06 (0.04, 0.09)	0.06 (0.04, 0.09)	0.08 (0.02, 0.17)
All cause mortality	0.76 (0.73, 0.79)	0.05 (0.03, 0.07)	0.05 (0.03, 0.06)	0.08 (0.03, 0.14)

Table S25: **Prediction of U-REGLE embeddings from 12-lead ECG and known cardiovascular features (Supplementary Table S22) in UKB.**

Pheno	AUROC	AUPRC	Top 1% prevalence	Top 10% prevalence
Hypertension	0.82 (0.81, 0.83)	0.67 (0.65, 0.68)	0.76 (0.72, 0.78)	0.90 (0.84, 0.96)
Diabetes type 2	0.84 (0.82, 0.86)	0.41 (0.36, 0.45)	0.26 (0.23, 0.29)	0.82 (0.73, 0.89)
Cardiovascular disease	0.81 (0.79, 0.82)	0.67 (0.65, 0.69)	0.77 (0.74, 0.80)	0.91 (0.84, 0.96)
Myocardial infarction	0.75 (0.72, 0.78)	0.14 (0.11, 0.17)	0.15 (0.12, 0.17)	0.27 (0.18, 0.36)
Afib	0.74 (0.71, 0.76)	0.13 (0.10, 0.16)	0.14 (0.12, 0.16)	0.21 (0.13, 0.32)
Coronary artery disease	0.78 (0.76, 0.81)	0.24 (0.21, 0.27)	0.24 (0.21, 0.26)	0.46 (0.34, 0.58)
Stroke	0.73 (0.69, 0.77)	0.06 (0.04, 0.08)	0.06 (0.04, 0.08)	0.07 (0.03, 0.12)
All cause mortality	0.76 (0.72, 0.79)	0.04 (0.03, 0.06)	0.05 (0.03, 0.06)	0.06 (0.01, 0.11)

Table S26: **Prediction of M-REGLE embeddings from ECG lead I+PPG and known cardiovascular features (Supplementary Table S22) in UKB.**

Pheno	AUROC	AUPRC	Top 1% prevalence	Top 10% prevalence
Hypertension	0.82 (0.81, 0.83)	0.66 (0.64, 0.68)	0.76 (0.72, 0.78)	0.89 (0.82, 0.94)
Diabetes type 2	0.84 (0.82, 0.86)	0.41 (0.36, 0.45)	0.26 (0.23, 0.29)	0.82 (0.73, 0.89)
Cardiovascular disease	0.80 (0.79, 0.82)	0.67 (0.65, 0.69)	0.77 (0.74, 0.80)	0.90 (0.83, 0.96)
Myocardial infarction	0.75 (0.71, 0.77)	0.14 (0.11, 0.17)	0.14 (0.12, 0.17)	0.26 (0.18, 0.35)
Afib	0.74 (0.71, 0.76)	0.13 (0.10, 0.16)	0.14 (0.12, 0.16)	0.22 (0.14, 0.31)
Coronary artery disease	0.78 (0.76, 0.81)	0.24 (0.20, 0.27)	0.24 (0.21, 0.27)	0.44 (0.32, 0.55)
Stroke	0.73 (0.70, 0.78)	0.06 (0.04, 0.08)	0.06 (0.05, 0.08)	0.08 (0.03, 0.15)
All cause mortality	0.76 (0.72, 0.79)	0.04 (0.03, 0.06)	0.05 (0.03, 0.06)	0.06 (0.01, 0.11)

Table S27: **Prediction of U-REGLE embeddings from ECG lead I+PPG and known cardiovascular features (Supplementary Table S22) in UKB.**

Embedding	Rx	Corr R	Odd Ratio
12 ECG	Statin	0.12	3.32
	HTN	0.13	4.51
	Insulin	0.02	20.00
ECG + PPG	Statin	0.12	1.14
	HTN	0.12	1.20
	Insulin	0.03	1.41

Table S28: **The maximum correlation and odd ratio between M-REGLE embeddings and medication status.**

See the attached Excel table.

Table S29: **LDSC heritability and intercept obtained for 96 embeddings obtained from 12-lead ECG.**

See the attached Excel table.

Table S30: **M-REGLE 12 lead ECG GWS loci.** CHR, chromosome; POS, base-pair variant position; EA, effect allele; NEA, non-effect allele; SRC, imputed or genotyped variant; INFO, imputation INFO score (set to 1 for genotyped variants); GWAS p-value. GENE_CONTEXT, genomic context of the variant. Notation for gene context:

- Overlapping gene(s):
  - `[A]`: variant overlaps gene `A`
  - `[A,B]`: variant overlaps genes `A` and `B`
- Downstream genes:
  - `[]A`: variant position is $0 < p \leq 10^3$ bp upstream of closest downstream gene `A`
  - `[]-A`: variant position is $10^3 < p \leq 10^4$ bp upstream of closest downstream gene `A`
  - `[]-A`: variant position is $10^4 < p \leq 10^5$ bp upstream of closest downstream gene `A`
  - `[]--A`: variant position is $10^5 < p \leq 10^6$ bp upstream of closest downstream gene `A`
  - `[]`: closest downstream gene is further than 10^6 bp
- Upstream genes: mirrors downstream gene notation, e.g., `B-[]` means variant position is $10^3 < p \leq 10^4$ bp downstream of closest gene `B`.

Method	Num Discovered Hits	Num Discovered Loci
PCA-unimodal	167 (148, 88.62%)	104 (94, 90.38%)
PCA-multimodal	215 (191, 88.84%)	122 (108, 88.52%)
CAE-unimodal	188 (166, 88.30%)	114 (103, 90.35%)
CAE-multimodal	227 (203, 89.43%)	131 (116, 88.55%)
VAE-unimodal (U-REGLE)	200 (182, 91.00%)	119 (107, 89.92%)
VAE-multimodal (M-REGLE)	262 (231, 88.17%)	142 (122, 85.92%)

Table S31: **Comparison of rediscovered hits and loci by different models and learning methods on 12-lead ECG.** The numbers in each parenthesis are the number and percentage of rediscovered hits/loci in GWAS catalog.

Method	Modality	$E[\chi^2]$	se($E[\chi^2]$)	CI
PCA	unimodal	71.54	1.38	(68.85, 74.3)
PCA	multimodal	90.98	1.68	(87.74, 94.3)
CAE	unimodal	88.22	1.77	(84.84, 91.77)
CAE	multimodal	99.24	1.93	(95.53, 103.05)
VAE	unimodal	92.22	1.82	(88.75, 95.84)
VAE	multimodal	112.48	2.06	(108.67, 116.76)

Table S32: **Comparison of expected Chi-Square statistics of GWAS Catalog variants by different models and learning methods on 12 lead ECG dataset.**

See the attached Excel table.

Table S33: **M-REGLE additional loci associated with cardiovascular features in Open Targets.** We detected 11 loci that were not discovered before for ECG feature GWAS. CHR, chromosome; POS, base-pair variant position; EA, effect allele; NEA, non-effect allele; Open Target, Open Target link to know association; Trait, the name of cardiovascular traits with the highest significance level; P-value; p-value of the highest significance level; Beta, effect size beta; Odd Ratio, odd ratio; and PMID; the previously reported study.

Modality	Merge	Model	Num loci	Num cardio enrichment
ECG	Single	PCA	104	47
ECG	Single	CAE	114	48
ECG	Single	VAE	119	68
ECG	Multi	PCA	122	48
ECG	Multi	CAE	131	63
ECG	Multi	VAE	142	61
ECG+PPG	Single	PCA	44	35
ECG+PPG	Single	CAE	54	57
ECG+PPG	Single	VAE	54	61
ECG+PPG	Multi	PCA	45	32
ECG+PPG	Multi	CAE	59	50
ECG+PPG	Multi	VAE	61	69

Table S34: **Comparison of GREAT enriched cardiovascular term counts by different models and learning methods.**

$Model_1$	$Model_2$	Stronger model	Nominal P-value
ECG Single PCA	ECG Single CAE	-	0.062
ECG Single PCA	ECG Single VAE	Single VAE	3.0e-41
ECG Single CAE	ECG Single VAE	Single VAE	2.3e-43
ECG Single PCA	ECG Multi PCA	Multi PCA	3.9e-13
ECG Single CAE	ECG Multi CAE	Multi CAE	1.1e-29
ECG Single VAE	ECG Multi VAE	Multi VAE	0.022
ECG Multi PCA	ECG Multi CAE	Multi CAE	1.5e-11
ECG Multi PCA	ECG Multi VAE	Multi VAE	2.0e-19
ECG Multi CAE	ECG Multi VAE	Multi VAE	8.2e-6
ECG+PPG Single PCA	ECG+PPG Single CAE	Single CAE	1.3e-13
ECG+PPG Single PCA	ECG+PPG Single VAE	Single VAE	1.4e-5
ECG+PPG Single CAE	ECG+PPG Single VAE	Single CAE	8.9e-5
ECG+PPG Single PCA	ECG+PPG Multi PCA	Single PCA	1.1e-11
ECG+PPG Single CAE	ECG+PPG Multi CAE	Multi CAE	1.4e-5
ECG+PPG Single VAE	ECG+PPG Multi VAE	Multi VAE	1.9e-14
ECG+PPG Multi PCA	ECG+PPG Multi CAE	Multi CAE	4.2e-20
ECG+PPG Multi PCA	ECG+PPG Multi VAE	Multi VAE	3.7e-32
ECG+PPG Multi CAE	ECG+PPG Multi VAE	Multi VAE	5.0e-14

Table S35: **Comparison of significance of significantly enriched cardiovascular term p-values by different models and learning methods.** P-values were computed by two-sided paired T-test on $-\log_{10}$-transformed ontology term nominal p-values.

See the attached Excel table.

Table S36: **LDSC heritability and intercept obtained for 12 embeddings obtained from ECG lead I and PPG.**

See the attached Excel table.

Table S37: **M-REGLE lead I ECG and PPG loci.** CHR, chromosome; POS, base-pair variant position; EA, effect allele; NEA, non-effect allele; SRC, imputed or genotyped variant; INFO, imputation INFO score (set to 1 for genotyped variants); GWAS p-value. GENE_CONTEXT, genomic context of the variant. Notation for gene context:

- Overlapping gene(s):
  - `[A]`: variant overlaps gene `A`
  - `[A,B]`: variant overlaps genes `A` and `B`
- Downstream genes:
  - `[]A`: variant position is $0 < p \leq 10^3$ bp upstream of closest downstream gene `A`
  - `[]-A`: variant position is $10^3 < p \leq 10^4$ bp upstream of closest downstream gene `A`
  - `[]-A`: variant position is $10^4 < p \leq 10^5$ bp upstream of closest downstream gene `A`
  - `[]--A`: variant position is $10^5 < p \leq 10^6$ bp upstream of closest downstream gene `A`
  - `[]`: closest downstream gene is further than 10^6 bp
- Upstream genes: mirrors downstream gene notation, e.g., `B-[]` means variant position is $10^3 < p \leq 10^4$ bp downstream of closest gene `B`.

Method	Num Discovered Hits	Num Discovered Loci
PCA-unimodal	69 (67, 97.10%)	44 (43, 97.73%)
PCA-multimodal	69 (68, 98.55%)	45 (45, 100.00%)
CAE-unimodal	77 (74, 96.10%)	54 (52, 96.30%)
CAE-multimodal	93 (89, 95.70%)	59 (57, 96.61%)
VAE-unimodal (U-REGLE)	89 (85, 95.51%)	54 (52, 96.30%)
VAE-multimodal (M-REGLE)	103 (99, 96.12%)	61 (59, 96.72%)

Table S38: **Comparison of rediscovered hits and loci by different models and learning methods on Lead I ECG + PPG dataset.** The numbers in each parenthesis are the number and percentage of rediscovered hits/loci in GWAS catalog.

Method	Modality	$E[\chi^2]$	se($E[\chi^2]$)	CI
PCA	unimodal	33.12	0.68	(31.82, 34.48)
PCA	multimodal	33.23	0.69	(31.95, 34.66)
CAE	unimodal	37.93	0.73	(36.51, 39.41)
CAE	multimodal	46.05	0.89	(44.34, 47.85)
VAE	unimodal	44.37	0.88	(42.71, 46.16)
VAE	multimodal	51.66	0.97	(49.81, 53.62)

Table S39: **Comparison of expected Chi-Square statistics of GWAS Catalog variants by different models and learning methods on lead I ECG + PPG dataset.** CI: confidence interval.

See the attached Excel table.

Table S40: **M-REGLE loci improves phenotypic prediction compared to unimodal loci.**

Pheno	U-REGLE		M-REGLE	
	Standard	Refined	Standard	Refined
Afib	0.57 (0.56, 0.58)	0.57 (0.56, 0.58)	0.59 (0.58, 0.60)	0.59 (0.58, 0.60)
MI	0.52 (0.51, 0.53)	0.53 (0.52, 0.54)	0.53 (0.52, 0.54)	0.53 (0.53, 0.55)
HTN	0.52 (0.51, 0.52)	0.52 (0.51, 0.52)	0.52 (0.52, 0.53)	0.52 (0.52, 0.53)
Stroke	0.50 (0.49, 0.52)	0.51 (0.50, 0.52)	0.50 (0.49, 0.52)	0.52 (0.51, 0.53)

Table S41: **AUROC comparison of M-REGLE and U-REGLE for standard and refined disease for 12-lead ECG.** We defined a refined version of the reference group for all diseases (e.g., Afib, myocardial infarction, hypertension, stroke), where the cases are again individuals who have the disease, but the controls are individuals who do not have any cardiovascular disease.

Pheno	U-REGLE		M-REGLE	
	Standard	Refined	Standard	Refined
Aifb	0.09 (0.08, 0.09)	0.14 (0.13, 0.14)	0.10 (0.09, 0.10)	0.15 (0.14, 0.16)
MI	0.06 (0.06, 0.07)	0.10 (0.09, 0.10)	0.06 (0.06, 0.07)	0.10 (0.10, 0.11)
HTN	0.41 (0.40, 0.42)	0.43 (0.42, 0.44)	0.41 (0.41, 0.42)	0.43 (0.42, 0.44)
Stroke	0.04 (0.03, 0.04)	0.06 (0.06, 0.07)	0.04 (0.03, 0.04)	0.07 (0.06, 0.07)

Table S42: **AUPRC comparison of M-REGLE and U-REGLE for standard and refined disease 12-lead ECG.** We defined a refined version of the reference group for all diseases (e.g., Afib, myocardial infarction, hypertension, stroke), where the cases are again individuals who have the disease, but the controls are individuals who do not have any cardiovascular disease.

Pheno	U-REGLE		M-REGLE	
	Standard	Refined	Standard	Refined
Aifb	0.56 (0.55, 0.57)	0.56 (0.55, 0.57)	0.56 (0.55, 0.57)	0.56 (0.55, 0.57)
MI	0.51 (0.50, 0.52)	0.51 (0.50, 0.52)	0.51 (0.49, 0.52)	0.51 (0.50, 0.53)
HTN	0.51 (0.51, 0.52)	0.52 (0.51, 0.52)	0.51 (0.51, 0.52)	0.51 (0.51, 0.52)
Stroke	0.51 (0.50, 0.52)	0.51 (0.49, 0.52)	0.51 (0.49, 0.52)	0.51 (0.49, 0.52)

Table S43: **AUROC comparison of M-REGLE and U-REGLE for standard and refined disease for ECG lead I + PPG.** We defined a refined version of the reference group for all diseases (e.g., Afib, myocardial infarction, hypertension, stroke), where the cases are again individuals who have the disease, but the controls are individuals who do not have any cardiovascular disease.

Pheno	M-REGLE		U-REGLE	
	Standard	Refined	Standard	Refined
Afib	0.08 (0.08, 0.09)	0.13 (0.13, 0.14)	0.08 (0.08, 0.09)	0.13 (0.13, 0.14)
MI	0.06 (0.06, 0.06)	0.09 (0.09, 0.10)	0.06 (0.06, 0.06)	0.10 (0.09, 0.10)
HTN	0.41 (0.40, 0.41)	0.42 (0.42, 0.43)	0.40 (0.40, 0.41)	0.42 (0.42, 0.43)
Stroke	0.04 (0.03, 0.04)	0.06 (0.06, 0.07)	0.04 (0.03, 0.04)	0.06 (0.06, 0.07)

Table S44: **AUPRC comparison of M-REGLE and U-REGLE for standard and refined disease ECG lead I + PPG.** We defined a refined version of the reference group for all diseases (e.g., Afib, myocardial infarction, hypertension, stroke), where the cases are again individuals who have the disease, but the controls are individuals who do not have any cardiovascular disease.

Method	AUROC	AUPRC	Top 1% prevalence	Top 5% prevalence
M-REGLE	0.55 (0.53-0.57) *	0.22 (0.21-0.24)	0.21 (0.10-0.34)	0.24 (0.18-0.30)
U-REGLE	0.53 (0.51-0.55)	0.21 (0.20-0.23)	0.27 (0.17-0.40)	0.25 (0.19-0.31)

Table S45: **Comparison of M-REGLE vs U-REGLE Afib prediction in Indiana Biobank dataset.** Star (*) indicates M-REGLE is significantly better than U-REGLE based on paired bootstrap test.

Method	AUROC	AUPRC	Top 1% prevalence	Top 5% prevalence
M-REGLE	0.58 (0.56-0.59)*	0.21 (0.20-0.22)*	0.25 (0.19-0.32)	0.24 (0.22-0.27)*
U-REGLE	0.55 (0.54-0.56)	0.19 (0.18-0.20)	0.22 (0.16-0.29)	0.20 (0.18-0.23)

Table S46: **Comparison of M-REGLE vs U-REGLE Afib prediction in EPIC-Norfolk dataset.** Star (*) indicates M-REGLE is significantly better than U-REGLE based on paired bootstrap test.

Phenotype	Method	Pearson R
PPG pulse rate	M-REGLE	0.10 (0.08-0.11)
PPG pulse rate	U-REGLE	0.09 (0.08-0.10)
SBP	M-REGLE	0.03 (0.01-0.04)
SBP	U-REGLE	0.02 (0.00-0.03)

Table S47: **Comparison of M-REGLE vs U-REGLE PPG pulse rate, ECG QT interval, and SBP prediction in EPIC-Norfolk dataset.**

Method	AUROC	AUPRC	Top 1% prevalence	Top 5% prevalence
M-REGLE	0.55 (0.52-0.58)	0.11 (0.09-0.13)*	0.16 (0.08-0.28)	0.16 (0.12-0.21)*
U-REGLE	0.53 (0.50-0.56)	0.10 (0.08-0.11)	0.09 (0.03-0.22)	0.09 (0.05-0.13)

Table S48: **Comparison of M-REGLE vs U-REGLE Afib prediction in BWHHS dataset.** Star (*) indicates M-REGLE is significantly better than U-REGLE based on paired bootstrap test.

Phenotype	Method	Pearson R
QT interval	M-REGLE	0.003 (-0.030-0.036)
QT interval	U-REGLE	-0.002 (-0.036-0.036)
PPG pulse rate	M-REGLE	0.08 (0.046-0.115)
PPG pulse rate	U-REGLE	-0.020 (-0.054-0.014)
SBP	M-REGLE	0.028 (-0.006-0.061)
SBP	U-REGLE	0.034 (0.0003-0.066)

Table S49: **Comparison of M-REGLE vs U-REGLE PPG pulse rate, ECG QT interval, and SBP prediction in BWHHS dataset.**

Variance explained	Multimodal	Unimodal
70%	6.17e-04 $\pm$ 6.07e-06	8.26e-04 $\pm$ 1.54e-05
83%	3.00e-04 $\pm$ 2.91e-06	2.71e-04 $\pm$ 2.43e-06
93%-94%	1.17e-04 $\pm$ 1.32e-07	1.27e-04 $\pm$ 1.12e-06
96%	7.2e-05 $\pm$ 1.04e-06	8.8e-05 $\pm$ 9.39e-07
97%	6.7e-05 $\pm$ 1.12e-06	8.1e-05 $\pm$ 8.1e-07
98%	6.4e-05 $\pm$ 8.94e-07	7.1e-05 $\pm$ 7.59e-07
99%	6.4e-05 $\pm$ 9.07e-07	7.2e-05 $\pm$ 7.11e-07

Table S50: **Comparison of multimodal reconstruction losses and an average of all unimodal reconstruction losses across different latent dimension numbers on lead I ECG + PPG + Spirogram dataset.**

Pheno	AUROC	AUPRC	Top 1% prevalence	Top 10% prevalence
Hypertension	0.64 (0.62, 0.66)	0.44 (0.42, 0.47)	0.53 (0.49, 0.57)	0.61 (0.47, 0.73)
Diabetes type 2	0.69 (0.65, 0.73)	0.11 (0.09, 0.13)	0.13 (0.10, 0.15)	0.22 (0.13, 0.31)
Cardiovascular disease	0.63 (0.61, 0.64)	0.45 (0.43, 0.48)	0.54 (0.50, 0.58)	0.56 (0.45, 0.69)
Myocardial infarction	0.63 (0.59, 0.67)	0.06 (0.05, 0.07)	0.06 (0.05, 0.09)	0.08 (0.02, 0.15)
Afib	0.65 (0.62, 0.69)	0.07 (0.05, 0.08)	0.08 (0.06, 0.09)	0.08 (0.02, 0.17)
Coronary artery disease	0.62 (0.59, 0.65)	0.11 (0.08, 0.13)	0.12 (0.09, 0.15)	0.24 (0.14, 0.36)
Stroke	0.57 (0.51, 0.62)	0.03 (0.02, 0.05)	0.04 (0.02, 0.05)	0.02 (0.00, 0.08)
Asthma	0.66 (0.63, 0.67)	0.27 (0.23, 0.29)	0.34 (0.30, 0.37)	0.43 (0.25, 0.53)
Angina	0.58 (0.55, 0.61)	0.07 (0.06, 0.09)	0.08 (0.06, 0.10)	0.13 (0.07, 0.23)
All cause mortality	0.58 (0.53, 0.64)	0.03 (0.02, 0.04)	0.03 (0.01, 0.04)	0.06 (0.02, 0.11)

Table S51: **Prediction of U-REGLE embeddings from ECG lead I + PPG + Spirograms.**

Pheno	AUROC	AUPRC	Top 1% prevalence	Top 10% prevalence
hypertension	0.66 (0.65, 0.68)	0.47 (0.45, 0.49)	0.56 (0.52, 0.60)	0.67 (0.55, 0.78)
diabetes type 2	0.70 (0.67, 0.74)	0.12 (0.09, 0.15)	0.13 (0.10, 0.15)	0.23 (0.14, 0.35)
cardiovascular disease	0.65 (0.64, 0.67)	0.48 (0.46, 0.51)	0.57 (0.53, 0.61)	0.64 (0.51, 0.76)
myocardial infarction	0.65 (0.61, 0.68)	0.07 (0.05, 0.10)	0.06 (0.05, 0.09)	0.14 (0.07, 0.24)
afib	0.66 (0.62, 0.70)	0.07 (0.06, 0.09)	0.09 (0.07, 0.10)	0.08 (0.02, 0.15)
coronary artery disease	0.64 (0.61, 0.67)	0.10 (0.08, 0.13)	0.11 (0.09, 0.14)	0.23 (0.11, 0.34)
stroke	0.60 (0.54, 0.65)	0.03 (0.02, 0.05)	0.03 (0.02, 0.04)	0.03 (0.00, 0.08)
asthma	0.67 (0.64, 0.68)	0.28 (0.25, 0.30)	0.36 (0.32, 0.39)	0.46 (0.35, 0.58)
angina	0.62 (0.58, 0.65)	0.08 (0.07, 0.10)	0.10 (0.07, 0.12)	0.16 (0.08, 0.26)
all cause mortality	0.59 (0.53, 0.64)	0.03 (0.02, 0.04)	0.03 (0.02, 0.04)	0.04 (0.00, 0.10)

Table S52: **Prediction of M-REGLE embeddings from ECG lead I + PPG + Spirograms.**
