# Supplementary material for: Applying multimodal AI to physiological waveforms improves genetic prediction of cardiovascular traits
Source: Am J Hum Genet. 2025 Jun 20;112(7):1562–79. doi: 10.1016/j.ajhg.2025.05.015 (PMC12256885; doi:10.1016/j.ajhg.2025.05.015)
Supplement: Document S2. Article plus supplemental information [file mmc3.pdf]

Applying multimodal AI to physiological waveforms improves genetic prediction of cardiovascular traits

Graphical abstract

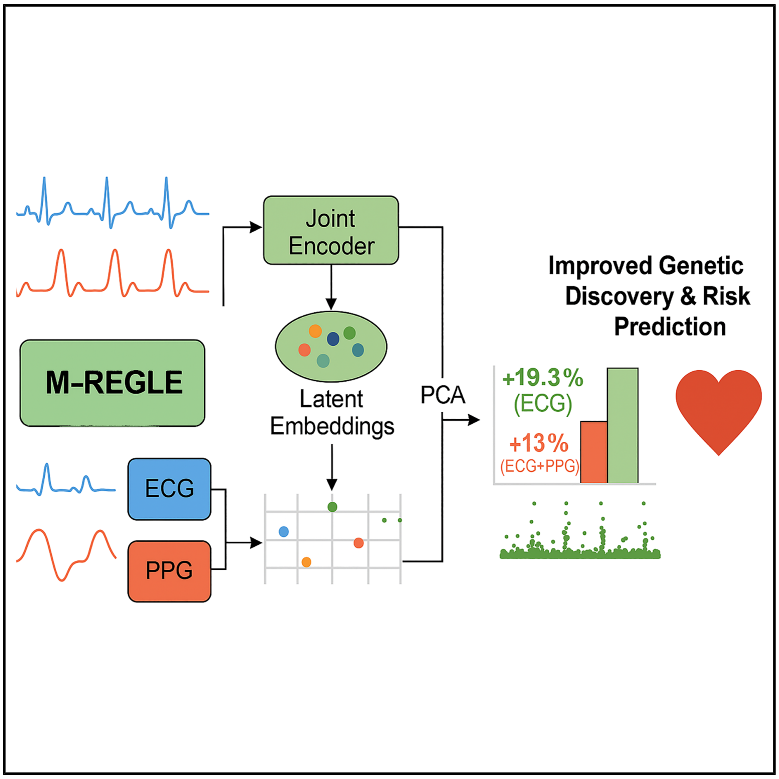

Authors

Yuchen Zhou, Justin Khasentino, Taedong Yun, ..., Cory Y. McLean, Babak Behsaz, Farhad Hormozdiari

Correspondence

babak.behsaz@gmail.com (B.B.),
fhormoz@google.com (F.H.)

Health data are inherently multimodal; we developed a multimodal AI (M-REGLE) that jointly analyzes complementary physiological waveforms (like ECG and PPG) to enhance genetic discovery for cardiovascular traits. Compared to analyzing waveforms individually, our approach identifies significantly more genetic associations and improves out-of-sample prediction for cardiac conditions, including atrial fibrillation.

Zhou et al., 2025, The American Journal of Human Genetics *112*, 1562–1579
July 3, 2025 © 2025 The Author(s). Published by Elsevier Inc. on behalf of American Society of Human Genetics.
https://doi.org/10.1016/j.ajhg.2025.05.015

ARTICLE

Applying multimodal AI to physiological waveforms improves genetic prediction of cardiovascular traits

Yuchen Zhou,[1] Justin Khasentino,[2] Taedong Yun,[1] Mahantesh I. Biradar,[3] Jacqueline Shreibati,[5] Dongbing Lai,[6] Tae-Hwi Schwantes-An,[6] Robert Luben,[3,4] Zachary R. McCaw,[7] Jorgen Engmann,[8] Rui Providencia,[9,10] Amand Floriaan Schmidt,[11,12,13] Patricia B. Munroe,[14] Howard Yang,[2] Andrew Carroll,[2] Anthony P. Khawaja,[3,4] Cory Y. McLean,[1] Babak Behsaz,[1,15,*] and Farhad Hormozdiari[1,15,*]

Summary

Electronic health records, biobanks, and wearable biosensors enable the collection of multiple health modalities from many individuals. Access to multimodal health data provides a unique opportunity for genetic studies of complex traits because different modalities relevant to a single physiological system (e.g., circulatory system) encode complementary and overlapping information. We propose a multimodal deep learning method, multimodal representation learning for genetic discovery on low-dimensional embeddings (M-REGLE), for discovering genetic associations from a joint representation of complementary electrophysiological waveform modalities. M-REGLE jointly learns a lower representation (i.e., latent factors) of multimodal physiological waveforms using a convolutional variational autoencoder, performs genome-wide association studies (GWASs) on each latent factor, then combines the results to study the genetics of the underlying system. To validate the advantages of M-REGLE and multimodal learning, we apply it to common cardiovascular modalities (photoplethysmogram [PPG] and electrocardiogram [ECG]) and compare its results to unimodal learning methods in which representations are learned from each data modality separately but are statistically combined for downstream genetic comparison. M-REGLE identifies 19.3% more loci on the 12-lead ECG dataset, 13.0% more loci on the ECG lead I + PPG dataset, and its genetic risk score significantly outperforms the unimodal risk score at predicting cardiac phenotypes, such as atrial fibrillation (Afib), in multiple biobanks.

Introduction

Health data (e.g., electrocardiograms [ECGs], photoplethysmograms [PPGs], spirograms, magnetic resonance imaging [MRI]) are valuable assets for clinical diagnosis, treatment, and prognosis and provide a unique opportunity for studying the genetic basis of complex traits.[1–9] Recent technological progress in electronic health record (EHR) systems enables access to multiple health-data modalities per individual.[10–12] When multiple clinical modalities pertain to a single organ system (e.g., circulatory system) or disease process (e.g., cardiovascular diseases), these modalities may encode complementary information. To maximize information for genetic association analyses, such as genome-wide association studies (GWASs), we propose to extract information from these modalities via a joint representation model. Frequently, the term "multimodal data" is used to refer to different sensory modalities or distinct data types for the same underlying phenomenon. For example, this would include utilizing spirograms, PPG, and ECG in one study. Conversely, "multiview data" is used to refer to data gathered from a single modality from different perspectives, such as different views of MRI (T1-weighted images and T2-weighted images) or different leads of an ECG. Although there is a subtle difference between multimodal and multiview data, in this work, we reference both types as multimodal, as the main objective of this work is to develop an AI model to incorporate multiple data sources.

In the past few years, a large body of work has utilized multimodal learning in medicine and biology. The existing efforts in medicine range from disease progression prediction,[13] disease subtyping,[14,15] healthcare applications,[12] disease diagnoses,[16] and recently general medical capabilities utilizing foundation models.[17–20] Furthermore, many biological datasets have utilized

[1]Google Research, Cambridge, MA 02142, USA; [2]Google Research, San Francisco, CA 94105, USA; [3]NIHR Biomedical Research Centre at Moorfields Eye Hospital & UCL Institute of Ophthalmology, London EC1V 9EL, UK; [4]MRC Epidemiology Unit, University of Cambridge, Cambridge CB2 0SL, UK; [5]Google, Mountain View, CA 94043, USA; [6]Department of Medical and Molecular Genetics, Indiana University School of Medicine, Indianapolis, IN 46202, USA; [7]Department of Biostatistics, University of North Carolina at Chapel Hill, Chapel Hill, NC 27599, USA; [8]Center for Translational Genomics, Population Science and Experimental Medicine, Institute of Cardiovascular Science, University College London, London, UK; [9]Institute of Health Informatics Research, University College London, London, UK; [10]Electrophysiology Department, Barts Heart Centre, St. Bartholomew's Hospital, London, UK; [11]Department of Cardiology, Amsterdam University Medical Centres, Amsterdam, the Netherlands; [12]Institute of Cardiovascular Science, University College London, London, UK; [13]Division of Heart and Lungs, University Medical Center Utrecht, Utrecht, the Netherlands; [14]William Harvey Research Institute, Barts and the London Faculty of Medicine and Dentistry, Queen Mary University of London, London, UK
[15]These authors contributed equally
*Correspondence: babak.behsaz@gmail.com (B.B.), fhormoz@google.com (F.H.)
https://doi.org/10.1016/j.ajhg.2025.05.015.
© 2025 The Author(s). Published by Elsevier Inc. on behalf of American Society of Human Genetics.
This is an open access article under the CC BY license (http://creativecommons.org/licenses/by/4.0/).

multimodal data such as chromatin accessibility, gene expression, protein, and others to improve the biological understanding of cell structure, subpopulations, and physiology.[21–25] In this work, we expand these recent efforts into the field of genomics. Recently, a method has been developed to perform GWAS on health data, called representation learning for genetic discovery on low-dimensional embeddings (REGLE).[8] REGLE uses convolutional variational autoencoders (VAEs) to compute a non-linear, low-dimensional, uncorrelated embedding of the data without clinical labels. Although REGLE's performance in genetic analysis and downstream polygenic risk scoring (PRS) surpasses prior methods,[8] it is limited to a single data modality. Recent studies have applied unsupervised learning methods to extract embeddings from multimodal data, such as ECG and MRI.[26,27] However, there are multiple ways to model multimodal data using unsupervised learning, including different fusion strategies, and prior work has not systematically explored or compared their effectiveness. In our work, we addressed this gap by investigating and comparing different multimodal modeling strategies and showing the benefits of using a joint representation model.

Different clinical modalities can include both complementary and overlapping information. Modeling each modality separately with unimodal representation learning, as in the REGLE framework, does not fully leverage these properties. We hypothesized that jointly modeling multiple complementary modalities would increase signal relative to noise by learning more informative representations, boosting the biological signal, and mitigate noise, thus improving genetic discovery and polygenic risk prediction. Furthermore, the shared information among related modalities is captured more efficiently in the joint embedding space, as opposed to having duplicated information in separate representations for each modality. This reserves more modeling capacity for identifying independent biological signals that are unique to each modality. Incorporating such signals can lead to groundbreaking genetic discoveries.

In this work, we proposed a method, called multimodal REGLE (M-REGLE), where we extended REGLE to incorporate multimodal data. In M-REGLE, we first conducted a test to check whether two modalities contain complementary information. If they do, we jointly learned a lower-dimensional representation (i.e., latent factors) of the multimodal health data (physiological waveforms) using convolutional VAEs[28] (i.e., using early fusion), orthogonalized the latent space, performed GWAS on each orthogonalized latent factor, and finally combined them to study the genetics of the underlying system. We validated the advantages of multimodal learning via M-REGLE by comparing it to unimodal learning in which representations were learned on each data modality separately, and downstream analyses were performed on the concatenation of all representations (i.e., late fusion). We call this unimodal REGLE (U-REGLE). Two sets of cardiovascular modalities in the UK Biobank dataset were used in the experiments: 12 leads of ECG as separate modalities and the lead I of ECG and PPG (the modalities prevalent in modern smart watches). In both sets of multimodal data, M-REGLE not only detected more previously unidentified loci but also significantly improved genetic risk scoring for cardiac phenotypes, such as atrial fibrillation (Afib).

Our main contributions are as follows: (1) we utilized 12-lead ECG and PPG data in UK Biobank to demonstrate the utility of multimodal modeling for genetic analysis and PRS. Most of the popular smart wearable devices contain sensors which allow users to record their ECG (equivalent to the lead I ECG) and PPG. Our experiments demonstrated that M-REGLE will enable researchers and users to optimally leverage this rapid acquisition of data for genetic discovery. (2) We observed that M-REGLE improved genomics discovery over existing methods for 12-lead ECG dataset and ECG lead I + PPG dataset for three metrics: Number of loci (19.3% more loci for 12-lead ECG and 13.0% more loci on the ECG lead I + PPG), number of hits (31.0% more loci for 12-lead ECG and 15.7% more loci on the ECG lead I + PPG), and expected chi-squared statistics as measure of power (22.0% higher $E[\chi^2]$ for 12-lead ECG and 16.4% higher $E[\chi^2]$ for ECG lead I + PPG). (3) We observed that PRS obtained from M-REGLE for 12-lead ECG significantly outperformed U-REGLE on cardiovascular traits and in particular Afib in UK Biobank. These results were independently validated in Indiana Biobank, EPIC-Norfolk datasets, and British Women's Heart and Health Study. (4) We observed that M-REGLE improved genomics discovery over U-REGLE for lead I ECG, PPG, and spirograms dataset.

Methods

Overview of M-REGLE

M-REGLE learns the low-dimensional embeddings of multiple modalities in a joint model to improve genetic analyses compared to learning embeddings from each modality individually then combining them for the genetic analyses. It is more effective in the presence of complementary and overlapping information across modalities. There are three steps in M-REGLE: (1) jointly learning a general non-linear, low-dimensional, largely uncorrelated representation from multimodal health data; (2) obtaining completely uncorrelated embeddings by computing principal components (PCs) of the embeddings; and (3) performing GWAS on each PC, combining them, then performing downstream analyses on combined summary statistics (Figure 1A).

In the first step, we combined the multimodal health data (e.g., by concatenation), and used the combined data as input to learn a low-dimensional representation by using a VAE. The VAE model contains two parts: an encoder that encodes the input data into lower-dimensional representations, and a decoder, which reconstructs the original data from the representation. During training, the VAE model learned to compress the information from multimodal data into a latent embedding vector with a prior that encouraged the VAE to learn uncorrelated

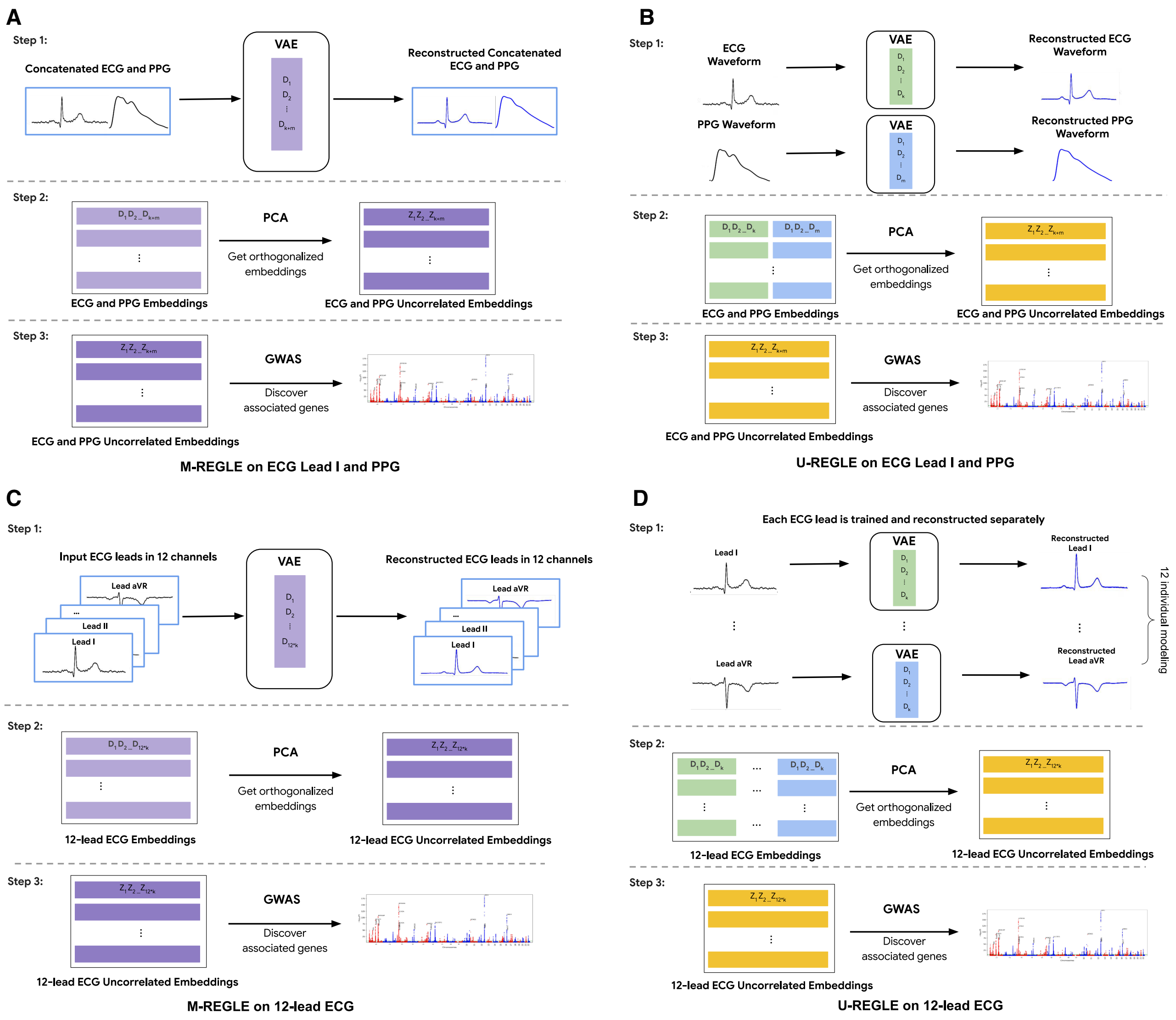

Figure 1. Overview of M-REGLE

(A) M-REGLE steps on lead I ECG and PPG, (B) U-REGLE steps on lead I ECG and PPG, (C) M-REGLE steps on 12-lead ECG, and (D) U-REGLE steps on 12-lead ECG. Step 1 in M-REGLE (A and C) obtains the raw embeddings from multimodal health data in a joint fit while step 1 in U-REGLE (B and D) obtains the raw embeddings for each modality separately. In step 2, to ensure completely uncorrelated embeddings, we applied PCA on the raw embeddings. Lastly, we ran GWAS on the uncorrelated embeddings and combined them.

embeddings. We obtained an *almost* uncorrelated joint representation for the input multimodal data. We observed a phenotypic correlation of <0.13 for 12-lead ECG (Figure S1) and < 0.05 for ECG lead I + PPG (Figure S2). In the second step, to ensure completely uncorrelated embeddings, we ran PC analysis (PCA) on the VAE embeddings to project them into completely uncorrelated PCs where PCA results are identifiable up to sign indeterminacies (unique eigenvalues) or rotational indeterminacies (repeated eigenvalues). In the final step, the PCs of the multimodal joint embeddings were used as synthetic phenotypes for GWASs. We performed GWASs on each PC of the embeddings for all individuals. We then combined the preliminary GWAS results by summing the chi-squared statistics of each phenotype,[29] deriving combined p values, and forming combined hits and loci (Figure 1A). It is worth mentioning that performing PCA on a multivariate outcome then summing the individual chi-squared statistics is a valid test (Table S1) and is equivalent to running the multivariate analysis of variance (M-ANOVA)[29,30] (Table S2). Finally, to obtain a polygenic risk score (PRS), we used elastic net regression to learn a linear combination of the hits. The main difference between M-REGLE and U-REGLE is that M-REGLE learns the embeddings of all modalities in a joint model, while U-REGLE learns the embedding for each modality separately (Figure 1B). In addition to comparing M-REGLE with U-REGLE, we also compared the representations learned by multimodal and unimodal PCA and regular (non-variational) convolutional autoencoders (CAEs).

Data preparation for ECG and PPG waveform

12-lead ECG data were sourced from UK Biobank field 20205. We used the at-rest, median heartbeat (median complex) waveforms.

This median complex is the composite of several consecutive complexes that are aligned and of which the median value was taken for each time point of the cardiac cycle. Each median waveform contains 600 points for all leads. The median data are cleaner compared to the raw ECG full waveform; therefore, it did not require extensive preprocessing. The PPG waveform was extracted from UK Biobank field 4205, but, unlike ECG, the PPG data have the median heartbeat waveform. Each PPG waveform contains 100 points, which are median values from single heartbeats. We made two multimodal datasets from the data: (1) 12-lead ECG as 12 different modalities, and (2) lead I of ECG and PPG. For both 12-lead ECG and PPG, we used data instance 2 in UK Biobank, which contains the most overlapping samples. We obtained 50,469 samples for 12-lead ECG and 55,041 samples of PPG as of April 17, 2023.

We first applied finite impulse response filters (FIRs) on all the ECG data to reduce noise. FIR filters are widely used in signal processing for removing unwanted frequency components from a signal. We set the cutoff frequencies to 0.05 and 40. We performed this step on ECG because it contains more noise compared to PPG. For quality control, we calculated several statistics (minimum, maximum, mean, and median) on each waveform and compared them with the statistics of all the other waveforms of the same type. We set a cutoff percentile of 0.1 and 99.9 for each statistic. The waveforms with at least one statistic that fell outside of these cutoffs were dropped. After this step, we had 48,259 samples for the 12-lead ECG and 41,282 samples in the ECG lead I and PPG dataset.

Dataset generation for machine-learning model

For both datasets, we created train/validation/test splits for hyper-parameter tuning, model evaluation, and a final test for the PRS model. We first created the splits by using a static hash on all the available ENCODED IDs (EIDs or participant identifier). Each EID was assigned to one of the training, validation, and test sets. Since we expect a uniform distribution from a hash function, the splits should exhibit the same properties for any feature of interest (e.g., the age of the individuals). The datasets were split by looking up each of the EIDs in the datasets in the created splits. The training, validation, and test dataset contained 70%, 20%, and 10% of the samples, respectively.

In order to standardize the magnitudes of waveforms from different modalities, we proceeded to scale each waveform type, ensuring their values fell approximately within $[-1, 1]$. We calculated the scale factor of each modality separately by picking the 90th percentile of the absolute maximum values of all waveforms of the modality in the training dataset. Therefore, we avoided the impact of outlier waveforms with extreme maximum values. We then applied the scales for each modality on all training, validation, and test datasets by dividing the waveforms with the corresponding scale factor.

Learning 12-lead ECG representations with generative models

Variational autoencoder (VAE) models were trained to learn lower-dimensional representations from 12-lead ECG data. Autoencoders comprise as encoder and decoder function approximators linked by a narrow bottleneck layer, and autoencoders condense input data into a concise set of numbers at the bottleneck layer, with the decoder subsequently reconstructing the input data from this condensed representation.[31] VAEs[28] represent a distinctive variant of autoencoders, introducing stochasticity into the encoding process. For M-REGLE, one VAE model was trained on the combined 12-lead ECG data to reconstruct the 12 leads together. For U-REGLE, 12 separate VAE models were trained on each ECG lead.

We used similar model structures for M-REGLE and U-REGLE. The encoder of VAE models consists of 1D convolutional layers, each followed by maximum pooling, then fully connected layers to generate the mean and variance of the bottleneck layer. The decoder architecture is a mirror image of the encoder. It starts with fully connected layers, followed by transpose convolutional layers, each prepended by an upsampling layer (supplemental note). For unimodal VAEs, the ECG lead was directly used as one input channel. In M-REGLE, the 12 leads were first stacked to create an input in the shape of 600 points $\times$ 12 channels. The VAE models for single lead ECG all had eight latent dimensions. We explored the latent dimension number by performing PCA using various PC numbers on the ECG leads. When we used eight PCs, it explained 0.93 of total variance of the data, demonstrating that eight latent dimensions is sufficient for a linear model to learn good representations from single ECG leads. We used 96 latent dimensions for multimodal VAE so that the total number of dimensions in the unimodal representations and multimodal representations were the same. The standard VAE loss function consisting of the reconstruction loss and the (rescaled) Kullback-Leibler (KL) divergence loss were used as the objective during the training of all the VAE models. The Adam optimizer was used to optimize the objective.

For each of the VAE models, we performed a large-scaled hyper-parameter search independently (Table S3). Each unimodal VAE model was trained for at most 300 epochs. The multimodal VAE models were trained for, at most, 1,200 epochs. Unimodal models needed fewer training epochs to converge due to their smaller model size. We verified that the validation loss curves for all VAE models plateaued within the number of epochs we selected (Figures S3 and S4). We additionally checked that, even if the unimodal models were trained for up to 1,200 epochs, the resulting embeddings had minimal differences from the embeddings obtained after up to 300 epochs (Figures S5–S17). After training, the checkpoints with the lowest validation loss were selected. We also used how uncorrelated the resulting representation coordinates were as a metric to pick the hyper-parameter sets. We calculated the correlation matrix of the representations that the model checkpoint generates on the validation dataset and found the maximum absolute value of the correlation coefficients (among the non-diagonal values of the correlation matrix), which we used as the metrics for the disentangleness of generated representations. To decide which hyper-parameter set to use, we first set a threshold of 0.1 on the maximum absolute correlation value and chose the one with the lowest validation reconstruction loss among all the models that passed the maximum absolute correlation threshold (Table S4). To ensure our models were robust against random seeds, we retrained the selected hyper-parameter combination of every modality and datasetting on 100 different random seeds. The retrained models all reached similar reconstruction loss and overall loss on the validation dataset (Figure S18A).

As a baseline, PCA were trained on each of the ECG 12 leads separately (unimodal PCA) and a combination of ECG 12 leads (multimodal PCA). We used eight PCs for PCA on a single ECG lead and 96 PC numbers for the PCA on joint ECG 12 lead. The learned PCs were used in the same way as the lower representations learned by VAE models in GWAS and downstream analyses.

Learning lead I ECG and PPG representations with generative models

VAE models were trained to learn representations of ECG lead I and PPG. We compared M-REGLE, where we trained one VAE model to reconstruct both waveforms together, and U-REGLE, where we trained two separate VAE models to learn representations of the waveforms independently.

We used the same model structure here as the 12-lead ECG models, which consist of 1D convolution layers and fully connected layers. For unimodal VAEs, the waveform was directly used as a one-channel input. In M-REGLE, we connected each individual's PPG waveform to their ECG waveform to form a 700-point-long one-channel input (supplemental note). We explored an alternative model architecture of using ECG and PPG (after upsampling) as two input channels, which led to inferior performance (Table S5). We used eight latent dimensions in the unimodal ECG model, four latent dimensions in the unimodal PPG model, and 12 latent dimensions in M-REGLE. We selected the latent dimension numbers by experimenting with different PC numbers.

The training, model-selecting strategy, and the baseline creation of ECG lead I and PPG followed the same procedure as the 12-lead ECG (Tables S3 and S4; Figure S18B).

Similar to the 12-lead ECG setting, we also created baselines using multimodal PCA and unimodal PCA. For multimodal learning, we first concatenated the ECG lead I and PPG waveform and then learned a PCA transformation that reduces the data to 12 dimensions. For unimodal learning, we learned a PCA that generates eight PCs for ECG lead I and a PCA that generates four PCs for PPG.

Learning lead I ECG and PPG and spirogram representations with generative models

To assess the generalizability of our findings across additional modalities, we also applied M-REGLE and U-REGLE to another set of multimodal data: lead I ECG, PPG, and spirogram. For M-REGLE, we first concatenated the three waveforms into a single sequence and trained one VAE model to jointly reconstruct all three modalities. For U-REGLE, we trained separate VAE models on each waveform independently. The ECG lead I and PPG data used are the same as in the previous experiment, and the spirogram data are also sourced from the UK Biobank data field 3066.[8] We used the same VAE architecture as in the previous two experiments. For U-REGLE, we used eight latent dimensions for ECG, four for PPG, and three for spirogram. For M-REGLE, we used a total of 15 latent dimensions to match the combined latent dimensionality of the three individual modalities (Figure S35).

GWASs

We generated unimodal representations and multimodal representations for the 12-lead ECG and the ECG lead I + PPG on the combination of the training and validation datasets. We performed PCA on each set of representations of the dataset to transform the representations as uncorrelated coordinates. Then preliminary GWASes were performed on each of the PCs of the representations for each datasetting and modality type using REGENIE.[32] All GWASes were adjusted for age, sex, body mass index (BMI), smoking status, genotyping array, and the top 15 genetic PCs.[7,8]

To get an overall result of genetic discovery on the multimodal waveform data, we combined the preliminary GWASs on the PCs of the representations. We computed the summation of the chi-squared statistics for each GWAS and computed the combined p value by applying the survival function of the combined chi-squared statistics using the number of the phenotypes as the degree of freedom. We utilized the combined chi-squared statistics to obtain the final GWAS result. More specifically, we considered that we had access to m phenotypes denoted by $P = [P_1, P_2, \ldots P_m]$ that were not correlated and where $\chi^2_{P_j,i}$ indicated the chi-squared statistics of i-th SNP for P_j phenotype. As phenotypes were not correlated, we computed chi-squared statistics for all phenotypes and then summed these statistics. The final p value was computed assuming that the summation followed a chi-squared distribution with m degree of freedom ($\sum_{j=1}^{m} \chi^2_{P_j,i} \sim \chi^2_{(m)}$).[29]

GWAS was restricted to individuals with European ancestry to minimize confounding. For quality control, we retained variants with minor allele frequency $\geq$0.001, imputation INFO score $\geq$0.8, missing call fraction $\leq$0.05, and Hardy-Weinberg equilibrium $p > 1.0\text{E}{-10}$, among all genotyped and imputed variants provided by UK BiobankUK Biobank. Genome-wide significant "hits" were defined as the most significant variants with $p < 5.0\text{E}{-8}$ and independent at $R2 < 0.1$ using the PLINK –clump command. A reference panel for linkage disequilibrium (LD) calculation contained 10,000 unrelated European samples from the UK Biobank. Significant "loci" were created based on the span of reference panel SNPs in LD (R2 $\geq$ 0.1) with the hits. Loci separated by fewer than 250 kb were subsequently merged.

GWAS Catalog loci replication

The number of hits and loci may be misleading because some of them might be false positives. However, if the hits/loci are known to be related to ECG or PPG traits, they are less likely to be false positive. We investigated relevant hits/loci by examining whether they are previously reported in the literature to be ECG or PPG related. We performed a GWAS Catalog search using key words (case insensitive), such as "electrocardio," "ecg," "pr interval," "ventricular rate," "pulse wave," and "notch position," (Table S6).

GWAS power analysis via expected chi-squared statistics

We compared the GWAS power of M-REGLE and U-REGLE in terms of the high-quality hits by calculating the mean of the chi-squared stats of all the rediscovered hits (i.e., the ones with support by GWAS Catalog) from the GWAS of each modality and datasetting.

Simulated data for statistical power comparison

Comparing PCA-based combined Chi-squared statistics and MANOVA

We performed a series of simulations to illustrate that PCA model power is equivalent to MANOVA. We simulated 10 factors using the following model:

$$F_i = Xb + e_i$$

F_i is the i-th factor, X is the set of variants (e.g., SNPs), and e models the environment or measurement noise. b is a vector of effect size where the first value is 0.001 (model alternative) and the rest are 0 (model null) and $e \sim MVN(0, \Sigma)$. Diagonal values of Σ are set to 1 and off-diagonal are set to ρ. We compare three models: naive model where we test each factor independently and then sum the chi square, PCA model where we perform

PCA on each factor and then sum the chi square, and finally MANOVA. We computed type I error and power for all three models when we set ρ to 0, 0.2, and 0.3 (Tables S1 and S2).

Simulated data for models with similar phenotype prediction and different statistical power

It is possible for two models to have very similar phenotype prediction while one has better GWAS power as it better captures the genetic signal. For simplicity of this toy example we assume we perform PCA. We assume the following generative model for our data modalities:

$$M_1 \sim MVN([F, 0.1 * s], I)$$
$$M_2 \sim MVN([F, 0.2 * s], I)$$

and the phenotype of interest is generated as follows:

$$Y = s * 0.05 + F + 0.8 * e$$

where F has two factors (i.e., non-genetic factors) and we have two data modalities (M_1 and M_2). Both data modalities are generated from the F factors and s (i.e., genetic factor), while the F factors capture the main signal of both modalities. Thus, in the U-style model with four total dimensions, the model learns the primary F factors from both modalities, leading to an overall U-style embedding of [F, F] that has no s component. In contrast, the M-style model can use just two dimensions to learn the contribution of F to both modalities, leading to an overall M-style embedding of [F, s]. Correlation of phenotype prediction between the models may remain very close given the strong contribution of F to phenotype; however, the M-style model will yield better genetic analysis and PRS prediction.

Functional enrichment using GREAT

Genomic Regions Enrichment of Annotations Tool (GREAT) enrichment analyses were performed on the human GRCh37 assembly using GREAT v4.0.4[33] using program defaults. Terms were considered statistically significant if the Bonferroni-corrected p values for both the region-based and gene-based tests were $\leq$ 0.05. Cardiovascular terms were identified by performing a case-insensitive match on enriched ontology term descriptions using the regular expression "`cardiac | cardio | cardial | heart | circulatory.`" Paired t tests were performed on $-\log_{10}$-transformed raw region-based p values for statistically significant cardiovascular terms.

Creating PRSs from significant hits

To evaluate the improvement of one model over another (e.g., M-REGLE vs. U-REGLE), we utilized phenotypic prediction as a measure of comparison. We created PRSs from the significant hits obtained for each model by training an ElasticNet model. The significant hits reported by each model were used as features for ElasticNet, with the prediction target set as the phenotype of interest (e.g., Afib). The same training set that was used to train M-REGLE was used to fit ElasticNet and we performed 5-fold cross-validation to find the right hyperparameters. We applied `sklearn.linear_model.ElasticNet` to train our ElasticNet and performed a hyper-parameter search of `l1_ratio` over [0.1, 0.5, 0.7, 0.9, 0.95, 0.99, 1.0] values.

All the PRS values reported in the UK Biobank were obtained from the test dataset. Furthermore, we shared the variant weights derived from significant hits in the UK Biobank with our collaborators for assessment on the EPIC-Norfolk[34] and Indiana Biobank datasets.[35] Our collaborators assessed PRS across the phenotypic data available to them. Consequently, we obtained phenotype-prediction results for the Afib phenotype in the Indiana Biobank dataset,[35] while, in the EPIC-Norfolk dataset, we obtained results for Afib, PPG pulse rate, and systolic blood pressure (SBP).

PRS validation on multiple datasets

Indiana Biobank dataset

Indiana Biobank samples were genotyped using Illumina Infinium Global Screening Array (GSA, Illumina, San Diego, CA) by Regeneron (Tarrytown, NY). Variants with palindromic alleles, missing rate >5%, minor allele frequency (MAF) <3%, and Hardy-Weinberg equilibrium P < 1E−4 were excluded. PCs of population stratification were calculated using Eigenstrat.[36] Based on the first two PCs, those clustered with the European reference samples from the 1000 Genomes Project[37] were grouped as European samples and they were used in this study (N = 4,030). Indiana Biobank samples were imputed using the Michigan Imputation Server[38] with the 1000 Genomes project as the reference panel. Variants with imputation INFO < 0.3 or MAF < 0.01 were excluded. PRS were calculated by using imputation dosages. Afib cases ($n = 778$) were determined based on International Classification of Diseases (ICD-9) (427.3, 427.31, and 427.32) and ICD-10 (I48.0, I48.1, I48.2, and I48.9) codes. Those not having Afib in their electronic health records were considered as controls (n = 3,252).

EPIC-Norfolk dataset

Genotyping in EPIC-Norfolk used the Affymetrix UK Biobank Axiom Array with the Axiom GT1 algorithm used for calling. Quality-control exclusions were made for variants with Hardy-Weinberg P < 1.0E−6 and checks made for abnormal clustering or plate batch effect. Samples were excluded for SNP call rate <97% with further checks for heterozygosity and gender. Imputation was performed by the Sanger Imputation Service using the IMPUTE4 software and Haplotype Reference Consortium and UK10K plus 1000 Genomes phase 3 reference panels. Variants with a genotyping quality score (INFO) <0.4 were excluded. Afib (n = 3,554) was ascertained using Hospital Episode Statistics (HES) records maintained in a database containing medical records for all UK National Health Service (NHS) hospitals. Linkage to cohort participants used the unique NHS number. Cases were defined as ICD-10 I48.0, I48.1, I48.2, I48.9, or I48 unspecified (coded as I48X). Controls (n = 18,002) were defined as non-cases with available GWASs.

British Women's Heart and Health Study

The British Women's Heart and Health Study (BWHHS) utilized genotyping techniques employed in the Human Cardiovascular Disease (HumanCVD) BeadChip, also known as the ITMAT-Broad-CARe (IBC) v2 array. This array contained up to 49,240 SNPs. To ensure the reliability of the data, rigorous quality-control measures were implemented. All SNPs were clustered into genotypes via Illumina Beadstudio software and stringent quality-control filters were applied at both the sample and SNP level. Samples failing to meet criteria, including individual call rates below 90%, gender mismatches, and duplicate discordance, were excluded. Similarly, SNPs with call rates below 95% or exhibiting Hardy-Weinberg disequilibrium with a p < 1.0E–6 were removed. Furthermore, to capture low-frequency variants of significant effect across the extensive dataset, filtering was conducted based on an MAF threshold of less than 0.01. Incidence cases of Afib were identified through general practitioner (GP) information provided during BWHHS record reviews. Additionally,

prevalent Afib cases were identified through ECG diagnosis, ensuring a comprehensive assessment of Afib prevalence within the study population.

Statistical tests for performance metrics

To assess the performance of phenotype-prediction and PRS models, we applied nonparametric bootstrapping. We created 100 bootstrap samples by randomly resampling the target dataset with replacement. We calculated the target metrics for each sample and reported the mean and standard error of the resulting distribution. To assess whether the performance difference between two models was statistically significant, we used paired nonparametric bootstrapping. For each of the 100 bootstrap samples, we calculated the difference of the target metrics by the two models. We reported that the first model had a significantly larger metric value than the second if the mean differences were positive and the confidence interval did not include zero. In contrast, the second model was reported to have significantly larger metric value if the mean difference was negative and the confidence interval did not include zero. However, if the confidence interval overlapped with zero, we concluded that the difference between the models was not statistically significant.

Results

Overview of cardiovascular modalities

We used the ECG and PPG health data in UK Biobank to define two multimodal tasks. Although both ECG and PPG are electrophysiological waveforms and are often analyzed together in healthcare settings, different sensor technologies are utilized to measure different physiological quantities: PPG employs optical sensors to detect volumetric changes in blood flow, while ECG relies on electrical sensors to measure the heart's electrical activity. Therefore, we considered them as distinct data modalities in this work. First, we used the at-rest, median complex ECG waveforms from UK Biobank. This median complex is the composite of several consecutive complexes that are aligned and of which the median value was taken for each time point of the cardiac cycle. Second, we extracted median heartbeat PPG waveforms from UK Biobank. We studied two multimodal tasks: (1) 12-lead ECG at rest, where the leads form 12 different modalities; and (2) ECG lead I plus PPG (which are available in modern smartwatches). After performing quality control on the data, we created train, validation, and test splits for model training, hyper-parameter tuning and model evaluation, and PRS model evaluation, respectively (Figures S19 and S20). Overall, the training, validation, and test datasets contain 70%, 20%, and 10% of samples, and each dataset has similar phenotypic distributions (Figure S21; Table S7).

Multimodal training is more effective in the presence of complementary information across modalities. To understand signal overlap and whether jointly learning embeddings is helpful, we performed canonical-correlation analysis (CCA) to identify and measure the associations between the modalities. This measure ranges between 0 for no overlap and 1 for complete overlap. First, we performed both CCA and deep CCA (DCCA) to compute the maximum projected correlation between each pair of the 12 leads of ECG. We observed that, while some pairs have extremely high mapped correlation (e.g., 0.98 ± 0.01 correlation between contiguous leads V5 and V6), other leads have lower correlation and thus contain complementary information (e.g., 0.62 ± 0.01 correlation between limb lead III and precordial lead V2). Next, we analyzed ECG lead I plus PPG and observed a modest correlation, indicative of complementary signals (0.43 ± 0.01) (Table S8). As a reference point, we observed that the spirogram data—a measure of lung function—in UK Biobank[7,8] have a lower projected correlation with lead I ECG (0.29 ± 0.03) and PPG (0.37 ± 0.03). It is worth noting that we do expect to see some non-zero correlation as health data capture general health information of individuals such as age, sex, and BMI. Lastly, we observed a similar pattern when we utilized DCCA,[39] which relaxes the linear assumption in CCA (Figure S22; Table S8) and when we utilized dynamic time warping (Figure S23).

M-REGLE produces better learned representations

When modalities have complementary and overlapping information, multimodal learning is expected to use the latent coordinates more effectively and thus produce better representations. One way to quantify a better representation is to have a lower overall reconstruction error, given a fixed latent dimension. We verified this hypothesis by applying step 1 of M-REGLE and U-REGLE (i.e., jointly or separately learn representations from multimodal data) to both 12-lead ECG and lead I ECG and PPG tasks and compared the reconstruction results.

We observed that M-REGLE reduced reconstruction error compared to U-REGLE on the validation set of the 12-lead ECG task. For M-REGLE, all the modalities needed to be combined as one input. We stacked the 12-lead waveforms as 12 channels (i.e., similar to the three channels of a color image). Then we trained a VAE model with 96 latent dimensions on the training data to reconstruct the 12-lead waveforms all together. For U-REGLE, we trained a VAE model independently on each ECG lead, getting 12 different VAE models. Each VAE had eight latent dimensions. In total each sample had $12 \times 8 = 96$ latent dimensions, the same number as M-REGLE. We compared the mean squared error (MSE) of the reconstructed 12-leads ECG by M-REGLE with the average MSE of the 12 ECG leads reconstructed by U-REGLE (which makes them comparable). M-REGLE reduced the overall MSE by 72.5%. The lower MSEs of M-REGLE were achieved consistently against different random seeds that were used in model training (Figure S18A). We observed the same trend across different numbers of latent dimensions. While both M-REGLE and U-REGLE showed decreasing reconstruction loss as latent dimensions increased, they eventually reached a point of diminishing returns. At this stage, U-REGLE failed to recover all the signals that M-REGLE captured (Figure 2A; Table S9). In

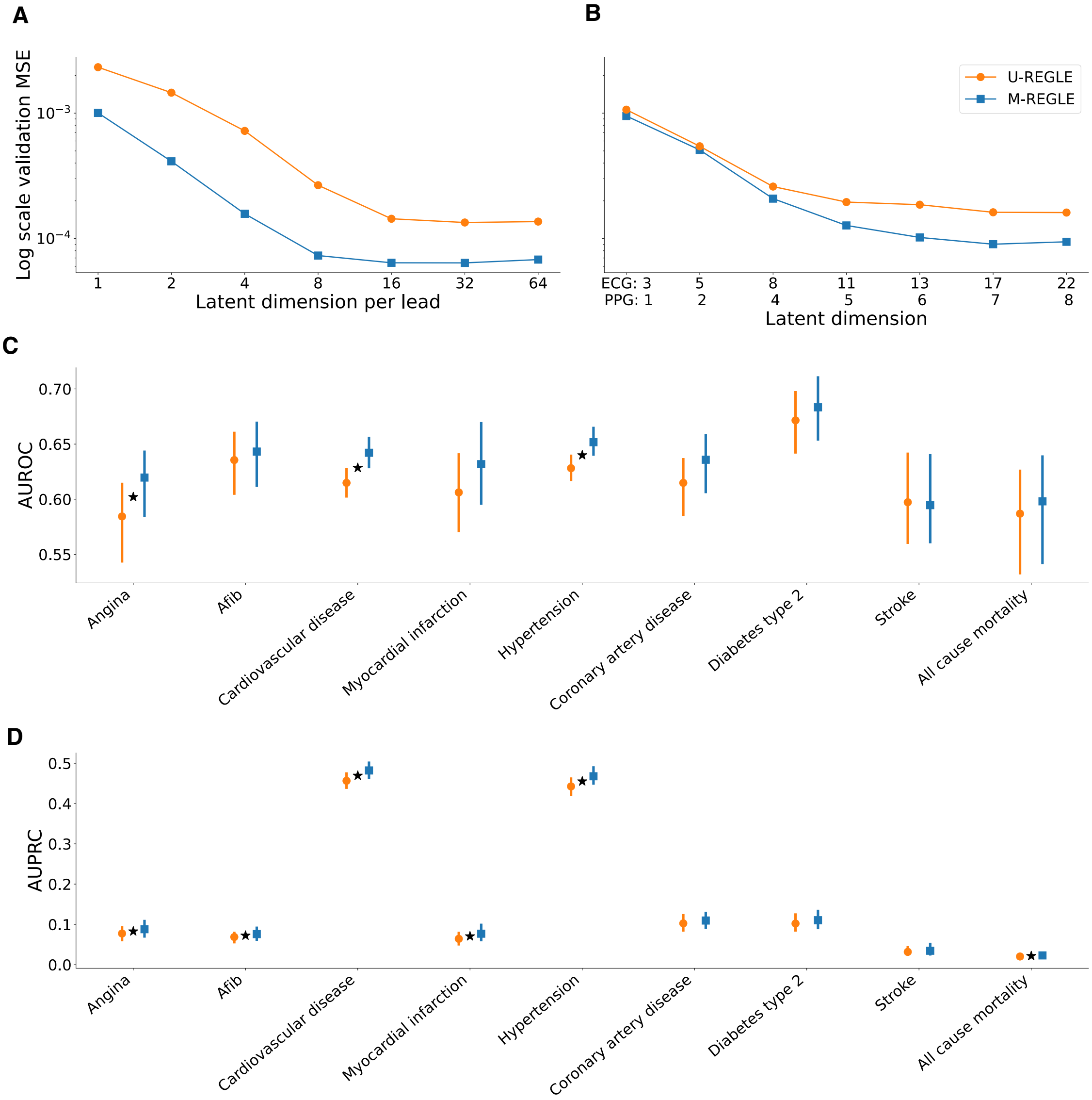

Figure 2. M-REGLE embeddings improve cardiovascular trait prediction
(A) Validation reconstruction losses in log scale of U-REGLE and M-REGLE on 12-lead ECG data. The *x* axis is the numbers of latent dimensions (1, 2, 4, 8, 16, 32, 64) per ECG lead. Standard errors (SEs) are too small to plot (see Table S9 for values of reconstruction losses and SEs. See Figure S25 for the normal scaled reconstruction loss plots.).
(B) Validation reconstruction losses in log scale of M-REGLE and U-REGLE across numbers of latent dimensions on lead I ECG and PPG data. The *x* axis is the numbers of latent dimensions used for each modality (see Table S13 for values of M-REGLE and U-REGLE reconstruction losses and SEs). All the difference between M-REGLE and U-REGLE in (A) and (B) are significant.
(C) AUROC prediction of nine phenotypes utilizing ElasticNet trained on the 12 embeddings obtained from ECG lead I and PPG.
(D) AUPRC prediction of nine phenotypes utilizing ElasticNet trained on the 12 embeddings obtained from ECG lead I and PPG.
Star sign indicates a statistically significant difference between the two methods using paired bootstrapping (100 repetitions) with 95% confidence.

addition, we compared multimodal PCA and CAE with unimodal PCA and CAE and the same trend was observed (Figure S24; Tables S10 and S11).

The hypothesis was also verified on the lead I ECG and PPG task. We applied M-REGLE on ECG lead I and PPG. To combine the two modalities, we concatenated ECG and PPG waveforms to form a long one-channel input. We then trained a VAE model that learns to reconstruct the concatenated ECG and PPG waveforms. The VAE model generated 12 latent dimensions from the concatenated ECG and PPG waveforms. In U-REGLE, we trained a VAE model for each modality independently. We used eight latent dimensions in the ECG VAE model and four latent dimensions in the PPG VAE model. We assigned more dimensions to ECG, since ECG is more complex (Table S12), and we observed comparable variance explained ratios in PCA when using eight and four as PC numbers for ECG and PPG. We compared the overall reconstruction loss of M-REGLE with the weighted average of the U-REGLE reconstruction losses (weighted by the waveform

lengths; see section "methods"). M-REGLE achieved 20.23% lower overall reconstruction loss compared to U-REGLE, and this performance was robust against random seeds that models were trained on (Figure S18B). This reduction is lower compared to 12-lead ECG (72.5% vs. 20.23%), possibly due to the lower observed correlation between lead I ECG and PPG compared to 12-lead ECG, as computed using CCA. We hypothesize that, when more complementary information is available, the gap will be larger between M-REGLE and U-REGLE reconstruction. We observed the same trend for different numbers of latent dimensions in VAE and different methods such as PCA and CAE (Figures 2B, S24, and S25; Tables S13–S15).

M-REGLE embeddings improve cardiovascular trait prediction over U-REGLE on ECG + PPG dataset

To understand the M-REGLE embeddings, we computed the phenotypic correlation between M-REGLE embeddings obtained from the 12-lead ECG and lead I plus PPG with 10,602 UK Biobank phenotypes. Since these embeddings are highly associated with covariates age, sex, height, BMI, and smoking status, we first removed (residualized up to second-order polynomial) the effect of these covariates from M-REGLE embeddings prior to computing the phenotypic correlation. We observed that 1,989 out of all combinations of M-REGLE embeddings by phenotype pairs (10,602 phenotypes and 108 embeddings from 12-lead ECG and lead I ECG and PPG) had significant correlations after Bonferroni correction. These correlations included very strong phenotypic correlations between M-REGLE embeddings and cardiac phenotypes indicating M-REGLE embeddings captured considerable cardiovascular information. Examples of these phenotypes were position of the pulse wave peak ($R = -0.69$; $p < 1.6E-300$) with second embedding of PPG and ECG lead I, ventricular rate ($R = 0.50$; $p < 1.77E-232$) with seventh embedding of PPG and ECG lead I, R axis ($R = -0.50$; $p < 3.25E-232$) with 96th embedding of 12-lead ECG, QT interval ($R = -0.42$; $p < 9.43E-158$) with fourth embedding of PPG and ECG lead I, QRS num ($R = 0.37$; $p < 3.80E-121$) with seventh embedding of PPG and ECG lead I, Pulse rate ($R = 0.36$; $p < 1.59E-118$) with fourth embedding of PPG and ECG lead I, and heart rate during PWA ($R = 0.36$; $p < 9.86E-117$) with seventh embedding of PPG and ECG lead I (Table S16).

To further investigate the correlation between M-REGLE embeddings and other phenotypes, we fitted single-task elastic net models on M-REGLE embeddings to predict nine cardiovascular disease phenotypes in UK Biobank (Table S17). We used the 12 M-REGLE embeddings obtained from lead I ECG and PPG. We observed that, for the lead I ECG plus PPG embeddings, M-REGLE outperformed or matched U-REGLE in area under receiver operating curve (AUROC) and area under precision-recall curve (AUPRC) across all nine phenotypes (Figures 2C and 2D; Tables S18–S21). Based on a paired bootstrap analysis, M-REGLE was significantly better than U-REGLE, in terms of AUROC, for predicting hypertension, cardiovascular disease, and ever smoking, while M-REGLE was significantly better than U-REGLE, in terms of AUPRC, for predicting for Afib, cardiovascular disease, myocardial infarction, hypertension, and all-cause mortality. Although M-REGLE embeddings are created in an unsupervised manner, making them agnostic to any particular disease condition, these embeddings are nonetheless predictive of cardiovascular disease phenotypes. There are multiple established predictors or risk factors for cardiovascular disease, including age, sex, obesity, metabolic panel such as lipid, activity, and socioeconomic factors.[40,41] We investigated whether M-REGLE embeddings provide additional and complementary information to these known features (289 features; Table S22). We compared known features prediction with M-REGLE embeddings plus known features and observed in all cases including M-REGLE embedding obtained from 12-lead ECG or lead I ECG and PPG outperform known features alone (Tables S23–S27).

Finally, we observed modest associations between cardiovascular medications and M-REGLE 12-lead ECG embeddings and PPG + ECG embeddings as shown in Table S28 and Figures S26–S28. These results indicated that M-REGLE embeddings, which are learned in an unsupervised and disease-agnostic fashion, can capture information concerning disease and medication status. Note that no causal interpretation should be ascribed to these associations.

Interpretability of M-REGLE embeddings

To examine the association between M-REGLE embeddings and cardiac traits, we investigated how perturbing individual embedding coordinates affects the decoded waveforms. Specifically, we identified coordinates that best differentiate samples labeled as Afib from those without Afib by analyzing the distribution of each coordinate in healthy and unhealthy samples. Dimensions 4, 6, and 10 emerged as the most distinctive. To assess the impact of these dimensions, we selected M-REGLE embedding pairs of one healthy sample and one unhealthy sample, where they were maximally separated along a single distinctive coordinate (4, 6, or 10) while remaining similar across all other dimensions. We then interpolated between these embeddings in 20 evenly spaced steps along the distinctive coordinate and decoded each step using the M-REGLE VAE model. The resulting waveforms were analyzed to determine physiological changes.

Our findings indicate that the fourth dimension is associated with the QT interval in ECG signals and the presence of a PPG notch. As the embedding transitioned from healthy to unhealthy along this dimension, the decoded ECG exhibited a prolonged QT interval, while the PPG notch disappeared (Figure 3A). Similarly, the 10th dimension is linked to ECG amplitude, with a progressive decrease in amplitude as the embedding shifted from healthy to unhealthy along

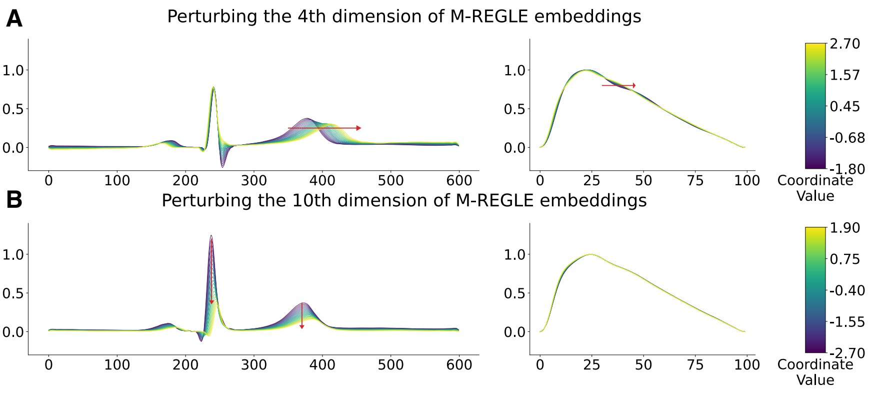

Figure 3. The effect of perturbing distinctive M-REGLE embedding dimensions on decoded ECG and PPG waveforms from a healthy toward an unhealthy sample
(A) The impact of the fourth dimension. As the embedding transitions from healthy to unhealthy, the decoded ECG (left) exhibits a prolonged QT interval, while the PPG (right) loses its notch.
(B) The impact of the 10th dimension. A shift from healthy to unhealthy along this dimension results in a progressive decrease in ECG amplitude.

this axis (Figure 3B).We validated these findings using an "average" M-REGLE embedding, where all coordinates were set to 0 (the mean value of the VAE prior). Next, we perturbed the 4th and 10th dimensions by varying their values between -2 and 2, corresponding to two standard deviations of the VAE prior. The decoded waveform showed the same effects from the two dimensions as our observations above (Figure S29).

M-REGLE on 12-lead ECG enhances genetic discovery

We compared the genetic discovery performance of M-REGLE with U-REGLE on the combined training and validation set ($n = 38{,}697$). M-REGLE generated 96 dimensional representations for each individual and then PCA was performed to project the raw embeddings into 96 uncorrelated PCs (i.e., there was no loss of information and this was done for orthogonalization). We performed GWAS on each of the 96 PCs using REGENIE[32] (Table S29). As the 96 PCs were uncorrelated, we combined these 96 GWASs by summing the chi-squared statistics and computed the final combined p value from this summation by choosing 96 degrees of freedom.[29] We observed similar genomics inflation of combined p value for M-REGLE and U-REGLE (Figures S30 and S31). We used the combined p values to create the hits and loci (see section "methods"). Hits were independent genome-wide significant (GWS) variants ($R^2 \leq 0.1$ and $P \leq 5 \times 10^{-8}$) and loci were obtained by merging hits within 250 kb together (Figure 4A).

M-REGLE had higher strength of association compared to U-REGLE (Figure 4B; Table S30) where M-REGLE discovered 262 hits (62 more than U-REGLE) and 142 loci (23 more than U-REGLE) (Figure 4C; Table S31). To compare our results with known loci previously detected for ECG and cardiovascular traits (Table S6), we compiled all 989 ECG-related trait hits and 498 loci from the GWAS Catalog. From 262 hits and 142 loci discovered by M-REGLE, 231 (88.5%) hits and 122 loci (85.92%) were previously reported (Figure 3C; Table S32) leaving 20 loci genome-wide not previously associated with these traits. Lastly, we observed that 11 out of 20 loci are unique to M-REGLE compared to U-REGLE (Figure 4C). To further validate these additional loci, we observed that 9 out of 11 loci have been detected for cardiovascular traits in Open Targets[42,43] (Table S33). To compare GWAS statistical power, we calculated the expected chi-squared statistics on all the variants reported as ECG-related traits in GWAS Catalog. We observed that M-REGLE had higher statistical power than U-REGLE ($E[\chi^2] = 112.48 \pm 2.05$ vs. $E[\chi^2] = 92.2 \pm 1.83$) (Figure 4D; Table S32).

As a secondary analysis, we used PCA and CAE in multimodal and unimodal settings to obtain latent representations. We observed similar results where multimodal learning led to better genetics discovery and higher statistical power. Multimodal PCA discovered 215 hits and 122 loci, while unimodal PCA found 167 hits and 104 loci (Table S31). Similarly, most of the hits and loci were known to be ECG-related traits (see section "methods"). As for statistical power, the expected chi-squared statistic of multimodal PCA was 90.98 ± 1.70, which is significantly higher than that of unimodal PCA ($E[\chi^2] = 71.53 \pm 1.38$). We observed similar trends for CAE (Table S32).

To assess functional enrichments, we used GREAT[33] to quantify the number of cardiovascular terms significantly associated with loci identified by each method as well as the statistical significance of enriched terms. For both multimodal and unimodal models, REGLE outperformed the corresponding PCA method in all cases, and it outperformed CAE in three of the four cases with respect to cardiovascular term enrichments (Table S34) and overall term significance (Table S35).

M-REGLE on ECG lead I and PPG enhances genetic discovery

Similar to the 12-lead ECG case, in the ECG lead I and PPG task, we observed that M-REGLE had stronger genetic discovery results than U-REGLE. We generated 12-dimensional joint representations for all individuals in the training and validation sets ($n = 33{,}192$) by running the M-REGLE model on concatenated ECG lead I and PPG waveforms, then performed PCA to obtain 12 uncorrelated PCs of the joint representations. We performed GWASs using REGENIE on each of the 12 PCs (Table S36) and combined the GWAS result by summing the chi-squared statistics of the 12 PC GWAS (Figure 5A).

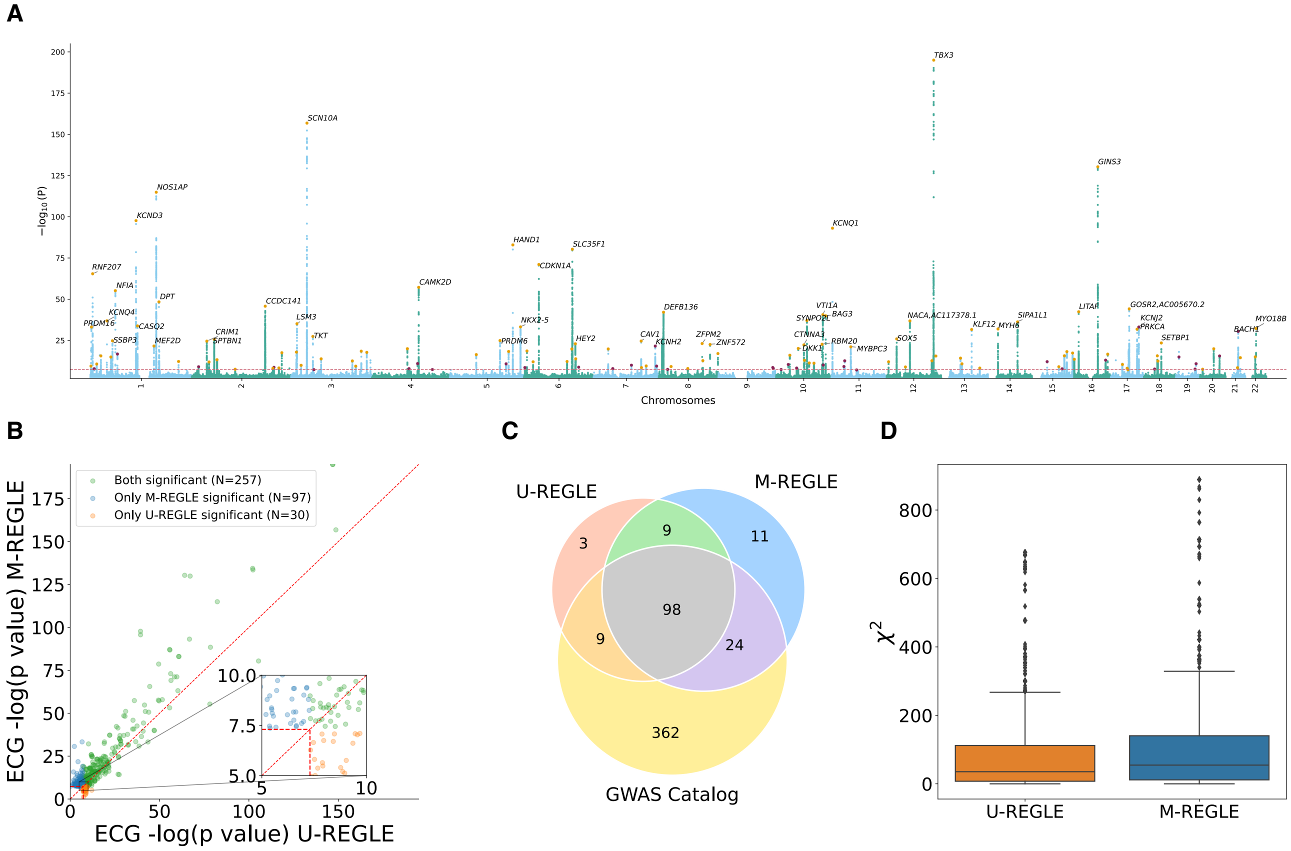

Figure 4. M-REGLE on 12 ECG leads increases genomic discovery

(A and B) (A) Manhattan plot depicting M-REGLE GWAS p values for all 22 autosomal chromosomes. Black gene names indicate the closest gene for each locus with $-\log_{10} p > 20$. Purple dots denote the GWS loci uniquely detected by M-REGLE. Orange dots indicate loci also identified in U-REGLE. (B) Comparison of M-REGLE GWS variants in hits with U-REGLE. The x axis is the $-\log p$ value of U-REGLE. The y axis is the $-\log p$ value of the M-REGLE. All p values in (A) and (B) are computed by summing the chi-squared statistics for all 96 embeddings to perform a single joint chi-squared test. The vertical and horizontal red lines indicate the GWS level. The diagonal red line indicates $y = x$. The orange dots indicate variants in hits that are significant for U-REGLE but not significant for our M-REGLE and green dots indicate variants in hits that are significant for our M-REGLE but not significant for U-REGLE.

(C) A three-way Venn diagram of the GWAS Catalog loci, loci discovered by M-REGLE, and loci discovered by U-REGLE.

(D) Comparison of the chi-squared statistics for all known significant variants in GWAS Catalog for both U-REGLE and M-REGLE. The difference is statistically significant.

We observed similar genomics inflation of combined p value for M-REGLE and U-REGLE (Figures S32 and S33).

M-REGLE has higher strength of association compared to U-REGLE (Figure 5B) where GWAS on M-REGLE embeddings detected 103 GWS hits and 61 loci, 14 and seven more than U-REGLE, respectively (Table S37). We compared the loci with GWAS Catalog. Out of 61 GWS loci detected by M-REGLE from lead I ECG and PPG, 59 fall in the known loci (96.72%) and two were not previously known (Figure 5C; Table S38). To further show that M-REGLE improves statistical power, we calculated the expected chi-squared statistics on all GWAS Catalog variants for ECG- and PPG-related traits. We observed a significant power increase for M-REGLE ($E[\chi^2] = 51.66 \pm 0.98$) compared to U-REGLE ($E[\chi^2] = 44.37 \pm 0.88$) (Figure 5D; Table S39). We observed the same pattern for CAE, but multimodal and unimodal PCA were comparable (Table S39).

Similar to 12-lead ECG, functional enrichments of cardiovascular terms for ECG lead I and PPG models showed superiority of multimodal and unimodal REGLE over PCA and CAE modeling methods for both cardiovascular term enrichments (Table S34) and overall term significance (Table S35).

M-REGLE improves the PRS for cardiovascular traits

To ensure the previously unidentified hits detected by M-REGLE are biologically important, we created genetic risk scores by extracting all variants from the significant hits. For each set of embeddings, we trained an ElasticNet model to predict the phenotype of interest on the train dataset utilizing all significant variants (see section "methods"). We considered multiple ECG-derived (e.g., ECG QT interval, ECG P axis,) and PPG-derived (e.g., PPG pulse velocity) phenotypes as well as nine additional cardiovascular disease or cardiovascular risk phenotypes for which there is extensive knowledge available on genetic associations such as Afib, angina, BMI, coronary artery disease, hypertension, stroke, myocardial infarction

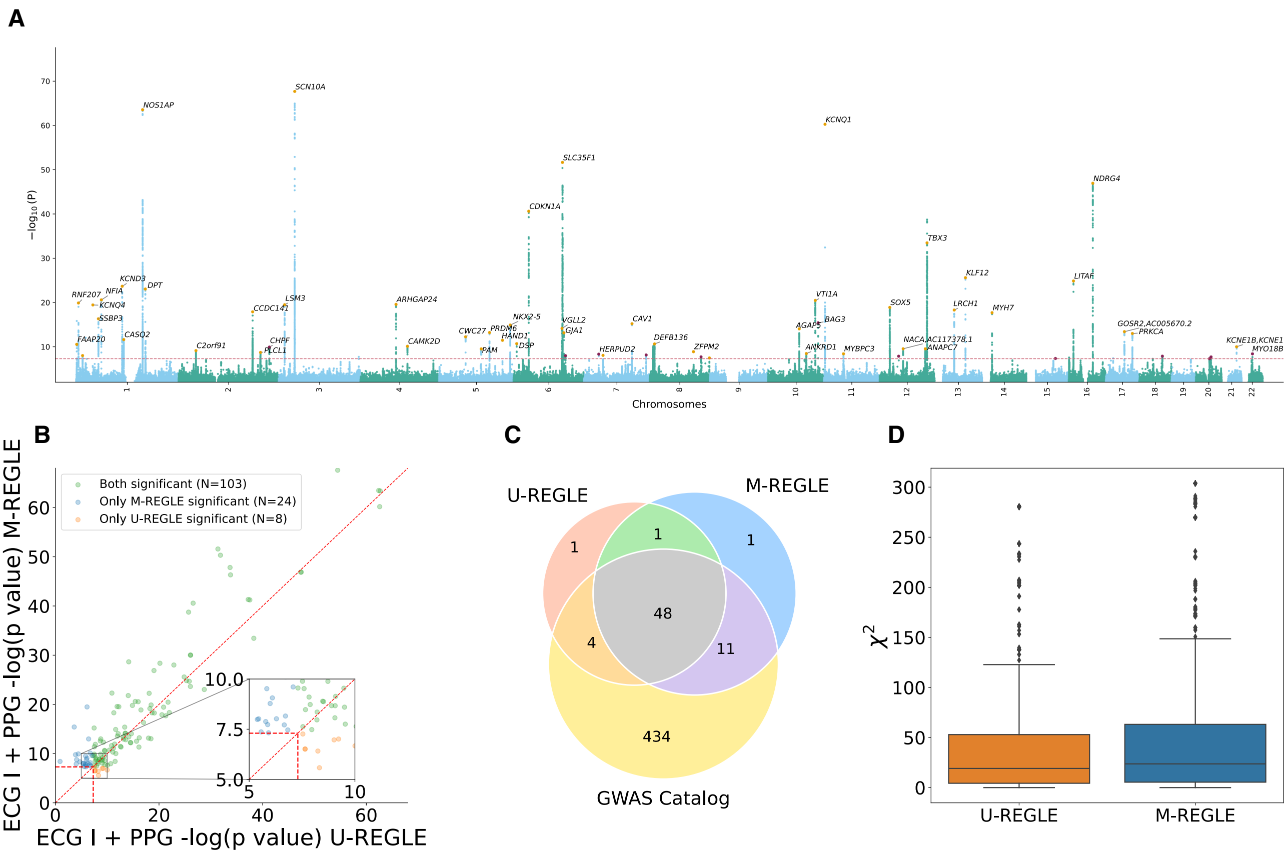

Figure 5. M-REGLE on ECG lead I and PPG increases genomic discovery
(A) Manhattan plot depicting M-REGLE GWAS p values. Black gene names indicate the closest gene for each locus with $-\log_{10} p > 20$. Purple dots denote the GWS loci detected uniquely by M-REGLE. Orange dots indicate loci also identified in U-REGLE.
(B) Comparison of M-REGLE GWS variants-in-hits with U-REGLE. The x axis is the $-\log p$ value of baseline. The y axis is the $-\log p$ value of the M-REGLE. All p values (A) and (B) are computed by summing the chi-squared statistics for all 12 embeddings to perform a single joint chi-squared test. The vertical and horizontal red lines indicate the GWS level. The diagonal red line indicates $y = x$. The orange dots indicate variants in hits that are significant for U-REGLE but not significant for our M-REGLE and green dots indicate variants in hits that are significant for our M-REGLE but not significant for baseline.
(C) A three-way Venn diagram of the GWAS Catalog loci, loci discovered by M-REGLE, and loci discovered by U-REGLE.
(D) Comparison of the chi-squared statistics for all known significant variants in GWAS Catalog for both U-REGLE and M-REGLE. The difference is statistically significant.

(MI), type 2 diabetes (T2D), and SBP. In UK Biobank, we have 22 derived phenotypes from ECG and PPG while in total we utilized 31 phenotypes.

We observed that genetic risk for 5 out of 31 phenotypes were significantly improved by M-REGLE compared to U-REGLE after Bonferroni correction (Table S40). The most notable improvement was observed for Afib (Figure 6), with M-REGLE showing an AUROC of 0.59 (95% confidence interval [CI], 0.582–0.599) versus U-REGLE AUROC of 0.57 (95% CI, 0.562–0.578) and an AUPRC of 0.10 (95% CI, 0.094–0.103) versus 0.09 (95% CI, 0.084–0.092) when utilizing all 12 ECG leads (Table S40). Additionally, other phenotypes such as PPG pulse rate, ECG QT interval, SBP, and height also exhibit significant improvement in phenotypic prediction with M-REGLE loci compared to U-REGLE loci (Table S40).

The main PRS analysis was done with a standard disease definition where we considered cases as individuals who have the disease, and control are individuals who do not have the disease. In addition, for each disease, we considered an analysis where the cases are defined as before, but the controls are individuals who do not have any cardiovascular disease. With the second, more-conservative definition, we observed similar results for AUROC but significantly improved results for AUPRC (Tables S41–S44).

To further validate our PRS results, we computed the PRS in Indiana Biobank datasets,[35] European Prospective Investigation into Cancer (EPIC)-Norfolk,[34] and the BWHHS[44–46] using the weights learned in the UK Biobank (see section "methods"). In the Indiana Biobank,[35] we have access to Afib and observed that, for all metrics, M-REGLE outperforms U-REGLE among European samples. In the case of AUROC, M-REGLE was significantly better (Table S45). In EPIC-Norfolk,[34] we have access to Afib, PPG pulse rate, ECG QT interval, and SBP. We observed that M-REGLE outperformed U-REGLE for all metrics. For Afib in particular, the AUROC, AUPRC, and top 5% prevalence were significantly better for M-REGLE

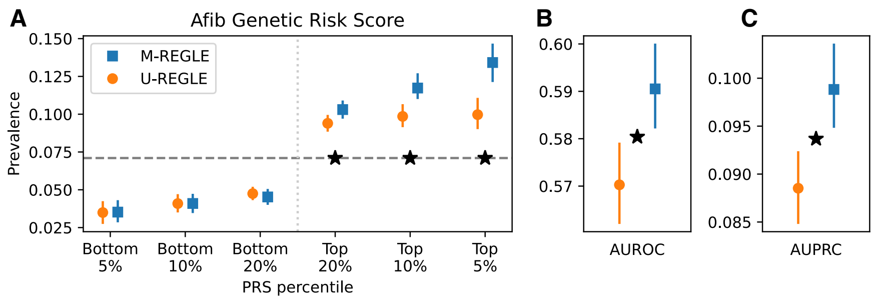

Figure 6. M-REGLE improves Afib genetic risk score
(A) The x axis is genetic risk score percentile and y axis is the prevalence. Lower is better for the bottom percentiles; higher is better for the top percentiles.
(B and C) (B) AUROC and (C) AUPRC (precision recall) Star sign indicates a statistically significant difference between the two methods using paired bootstrapping (100 repetitions) with 95% confidence.

compared to U-REGLE (Tables S46 and S47). Similar to EPIC-Norfolk, in BWHHS, we have access to Afib, PPG pulse rate, ECG QT interval, and SBP. We observed that, for all metrics, M-REGLE outperforms U-REGLE (Table S48) and, for Afib, AUPRC and top 5% prevalence were significantly better for M-REGLE compared to U-REGLE (Table S48). Lastly, for all other phenotypes, M-REGLE outperformed U-REGLE except for SBP in BWHHS (Table S49).

M-REGLE on ECG lead I and PPG and spirograms enhances genetic discovery

We conducted an experiment incorporating an additional data modality, the spirogram, alongside the ECG lead I and PPG dataset. We utilized a similar process and quality-control steps to those in our previous work for the spirogram data.[8] We showed that M-REGLE continues to outperform U-REGLE, particularly in terms of the reconstruction error, where M-REGLE achieves significantly lower MSEs than U-REGLE across various numbers of latent dimensions (Figure S35; Table S50). M-REGLE also achieved slightly better performance than U-REGLE in the downstream phenotype-prediction task (Tables S51 and S52).

We performed GWAS learned representations using the same steps utilized in the previous section. We observed that M-REGLE has higher power than U-REGLE (Figure S36). To compare GWAS statistical power, we calculated the expected chi-squared statistics on all the variants reported as ECG-related, PPG-related, and spirogram traits in GWAS Catalog (Table S6). We observed that M-REGLE had higher statistical power than U-REGLE ($E[\chi^2] = 22.93 \pm 0.49$ vs. $E[\chi^2] = 19.58 \pm 0.41$). These results highlight the adaptability of M-REGLE to new multimodal settings. It is worth noting that we cannot directly compare the power of M-REGLE on ECG lead I, PPG, and spirograms with previous results (ECG lead I and PPG) because the sample sizes are different. We have 37,144 samples for ECG lead I and PPG but only 27,213 for the combined analysis of ECG lead I, PPG, and spirograms.

Factors contributing to M-REGLE's superiority over U-REGLE in genetic studies

We considered two main reasons M-REGLE can outperform U-REGLE in genetic analyses (GWAS and PRS): first, M-REGLE finds better representations of health-data modalities and these embeddings present the phenotype of interest better, thus improving the downstream genetic analysis. M-REGLE would increase signal relative to noise by learning more informative representations, which can improve genetic analyses. Second, the shared information among related modalities is captured more efficiently in the joint embedding space, as opposed to having duplicated information in separate representations for each modality (Figure S34). We simulated data to show an example in which two data modalities have both genetic and non-genetic underlying factors, with the non-genetic factors driving the main phenotypic signal. Under this phenotypic architecture, we observe that both M-REGLE and U-REGLE can capture the strong non-genetic factors, but M-REGLE also demonstrates the capability to capture the genetic factors. Interestingly, both models have very similar phenotype prediction (Figure S34) but very different statistical power in detecting the genetic factor.

Discussion

Access to multiple health-data modalities for many individuals in biobanks and EHR systems provides unique opportunities to study the genetics of complex traits. In particular, when several data modalities are related to a single organ system, these modalities together can provide a better picture of the organ's function than any individual modality. However, we currently lack statistical methods to fully utilize these multimodal health data in genetic analyses. REGLE[8] provides a means to study the genetic underpinnings of high-dimensional data, but it is limited to one data modality and does not leverage the shared and complementary information in multimodal health data. Here, we developed an unsupervised representation learning method, M-REGLE, which aims to utilize multimodal health data in a joint model to improve genetic analyses. We showcased the advantages of M-REGLE over applying REGLE independently on different health-data modalities and then combining the results. M-REGLE creates embeddings in an *unsupervised* process that is agnostic to any disease status and medication; however, the obtained embeddings are associated with cardiovascular disease and medications.

We compared M-REGLE with the unimodal representation learning analog (U-REGLE) side by side on the same input data, and we observed significant performance improvements in several aspects. First, M-REGLE has lower reconstruction error compared to U-REGLE, which suggests it compresses and keeps more of the available signal in the dense representations for subsequent use in GWASs. Second, we validated that the joint embeddings learned by M-REGLE capture more genetic signals related to the input data compared to the collective U-REGLE embeddings and higher statistical power over known cardiovascular traits. Lastly, the genetic variants identified by M-REGLE enable better polygenic scoring for 14% (4 out of 28; excluding height from 5 out of 29 phenotypes reported in the "results" section) of tested cardiac phenotypes. Overall, M-REGLE provided greater statistical power for GWAS on health data, which could improve downstream applications such as biological discovery, better PRSs, and more possible drug targets. Additionally, we tested PCA and CAE as base models instead of VAE, applying the same M-style and U-style methods. Our results suggest that CAE and VAE were more effective at capturing meaningful signals and led to larger performance gaps between M-style and U-style methods (Tables S9–S11, S13–S15, S31, and S38). Based on these results, we recommend using a non-linear, deep-representation learning model alongside the M-style approach to maximize the extraction of informative signals from multimodal data.

The key for the effectiveness of M-REGLE is the existence of complementary and overlapping information in the modalities. On one end of the spectrum, when the information in different modalities is completely non-overlapping, there is no duplication of information (and consequently inefficiency). Hence, learning separately will not be wasteful, and M-REGLE is not expected to learn more effectively than U-REGLE. On the other hand, when some modality completely covers another modality, we will not expect to gain any boost from using M-REGLE either, because the duplicated information does not add additional signal to the learned representation. Health data of a single organ system or disease are usually measured through different methods and sensors and fall in the middle of this spectrum. However, before applying M-REGLE, we recommend performing a preliminary analysis to check that the modalities include complementary information (e.g., by performing CCA or DCCA).

With the presence of complementary and overlapping information, we expect that, when using a deep neural network, the compression of information in a low-dimensional dense representation happens more effectively and, as a result, we retain more signal that translates into better reconstruction by the decoder. Thus, the key question was whether the more effective compression also translates into better genetic discovery. We hypothesized that the answer would be positive, because, by using more than one source, the signal-to-noise ratio can be improved, which translates into better statistical power. On the one hand, the representation learning will be more efficient and can retain more information that improves the signal. Furthermore, the modality-specific noise can be reduced because one modality can correct the noise in the other. Our results using different data modalities, different learning methods, and multiple biobanks corroborate this hypothesis.

It is worth noting that our work has a connection to fusion models in deep learning. When dealing with multimodal data, there are three common paradigms often used when combining modalities. First, in early fusion, one combines modalities before the representation learning process and learns a joint latent that is used for the final task, such as prediction of Afib status. Second, in intermediate fusion, one learns representations for each modality separately, combines the independent representations (e.g., by concatenation), and performs the final task on this combined representation. Third, in late fusion, one learns the representations separately and also uses the separate latents to obtain a separate result using each modality for the final task. One then combines the final results. For example, we use PPG and ECG separately to obtain two separate probabilities for having Afib and then get their average as the final probability. Note that M-REGLE is an early fusion, while U-REGLE is an intermediate fusion. In contrast, a late-fusion method would be to run original REGLE on each modality separately (which includes the final task of GWAS) and then meta-analyze the resulting separate GWASs. We intentionally choose U-REGLE (i.e., intermediate fusion) as the baseline instead of the late fusion of separate REGLEs. This is to limit the difference to only multimodal vs. unimodal learning and have a better comparison.

Utilizing ECG parameters for cardiovascular disease have been well studied and multiple expert defined features (EDFs) have been shown to be important for cardiovascular disease. Some of these EDFs includes PR interval, where an abnormal PR interval is a risk factor for Afib and cardiovascular mortality[47–49]; QT interval, where a long QT interval is a risk factor for arrhythmic events and death[50,51]; P wave, which is associated with Afib[52–55]; and QRS complex, where QRS complex is associated with increased ventricular arrhythmic event and mortality.[56–58] Furthermore, there are large bodies of works that performed GWAS[58,59] on these EDFs; however, M-REGLE can utilize other electrophysiological waveforms in a joint model and can detect these embeddings unsupervised with no prior knowledge. Furthermore, we show that not only can M-REGLE GWASs rediscover known GWAS hits but they can also detect previously unidentified hits.

Our exploratory work on ECG and PPG data also opens up opportunities in the large-scale study of cardiovascular disease risk and the genetics of phenotypes derived from smart wearable or mobile phones. Most of the popular

smart wearable devices contain sensors that allow users to easily record their ECG (equivalent to the lead I ECG) and PPG. Our experiments demonstrate that M-REGLE will enable researchers to optimally leverage this newly abundant data for genetic discovery. In particular, we emphasize the importance of early fusion of the data before the representation learning and compression into low-dimensional data as there is a large gap between before and after fusion. This is in contrast with the usual practice of using meta-analysis after GWAS to combine multiple sources of data in genetic analysis.

Our work has several limitations. First, our aim was to focus on the method and illustrate the advantage of M-REGLE for genetic discovery over combining the results of unimodal representation learning. As a result, many aspects of genetic discovery can be improved, and the goal was not to have the strongest GWAS on cardiovascular phenotypes. Second, we utilized the median of waveforms of heartbeats for both PPG and ECG. Alternative preprocessing methods can be explored on full ECG and PPG waveforms, which potentially could produce more comprehensive representations, when the focus is on the genetic architecture of heart function. However, recent work[26] has shown that utilizing median ECG waveforms can reduce noise and improve results. Third, we performed the main experiments on samples with all the modalities available and have not fully explored M-REGLE's potential in handling missing data. Being able to learn joint representations from a multimodal dataset with missing data would enable us to carry out larger-scale genetic studies that cover all individuals with only some of the data modalities available. Fourth, our 12-lead ECG task does not exhibit all properties of multi-modality as the different leads conceptually measure the same type of signal (i.e., voltage) and are correlated, but they still have enough non-overlapping signal to be a good choice for a proof of concept as discussed in section "overview of cardiovascular modalities." Fifth, we observed that the value of β in β-VAE and scaling factor of inputs jointly have significant impact on M-REGLE embeddings (Figures S37 and S38), while we used a fixed scaling factor and performed hyper-parameter search over β to find the optimal β for the given scaling factor. A more systematic search over both β and scaling factor can produce better embeddings. Sixth, all the genetic analyses were performed on individuals of European ancestry, and the specific impact of ancestry on M-REGLE and U-REGLE was not explored. Lastly, the learned representations obtained from VAEs are known to be non-identifiable without further assumptions.[60] However, we observed that, in all of our experiments, the learned embeddings are robust up to a simple permutation and reversal of sign.

Despite these limitations, M-REGLE provides a means to better leverage multimodal health data for genetic discovery and has shown improved performance compared to unimodal representation learning. We demonstrated its advantages by using ECG and PPG data to study genetics of cardiovascular traits. We believe M-REGLE will become a standard method for using multimodal health data for GWAS and downstream analyses.

Data and code availability

Open-source code and trained model weights are available at https://github.com/Google-Health/genomics-research under the M-REGLE directory.

Acknowledgments

We thank all participants, dataset creators, and maintainers of UK Biobank, BWHHS, EPIC-Norfolk, and Indiana Biobank. This research has been conducted using the UK Biobank Resource under application number 65275. The EPIC-Norfolk study (https://doi.org/10.22025/2019.10.105.00004) has received funding from the Medical Research Council (MR/N003284/1 MC-UU_12015/1 and MC_UU_00006/1) and Cancer Research UK (C864/A14136). The genetics work in the EPIC-Norfolk study was funded by the Medical Research Council (MC_PC_13048). P.B.M. acknowledges the support of the National Institute for Health and Care Research Barts Biomedical Research Centre (NIHR203330). A.P.K. is supported by a UK Research and Innovation Future Leaders Fellowship, an Alcon Research Institute Young Investigator Award, and a Lister Institute of Preventive Medicine Award. This research was supported by the NIHR Biomedical Research Centre at Moorfields Eye Hospital and the UCL Institute of Ophthalmology. A.F.S. is supported by BHF grants PG/18/5033837, PG/22/10989, the UCL BHF Research Accelerator AA/18/6/34223 and by the UK Research and Innovation (UKRI) under the UK government's Horizon Europe funding guarantee EP/Z000211/1. A.F.S. received additional support from the National Institute for Health Research University College London Hospitals Biomedical Research Centre and the Rosetrees Trust (supplemental note).

Declaration of interests

Y.Z., J.K., T.Y., H.Y., A.C., C.Y.M., B.B., and F.H. are current or former employees of Google, and own Alphabet Inc stocks. This study was funded by Google LLC. A.P.K. has acted as a paid consultant or lecturer to Abbvie, Aerie, Allergan, Google Health, Heidelberg Engineering, Novartis, Reichert, Santen, Thea, and Topcon. A.F.S. has received funding from New Amsterdam Pharma for an unrelated project.

Supplemental information

Supplemental information can be found online at https://doi.org/10.1016/j.ajhg.2025.05.015.

Received: September 18, 2024
Accepted: May 30, 2025
Published: June 20, 2025

References

1. Elliott, L.T., Sharp, K., Alfaro-Almagro, F., Shi, S., Miller, K.L., Douaud, G., Marchini, J., and Smith, S.M. (2018). Genome-wide association studies of brain imaging phenotypes in UK

biobank. Nature *562*, 210–216. https://doi.org/10.1038/s41586-018-0571-7.

2. Jónsson, B., Bjornsdottir, G., Thorgeirsson, T., Ellingsen, L. M., Walters, G.B., Gudbjartsson, D.F., Stefansson, H., Stefansson, K., and Ulfarsson, M.O. (2019). Brain age prediction using deep learning uncovers associated sequence variants. Nat. Commun. *10*, 5409. https://doi.org/10.1038/s41467-019-13163-9.
3. Verweij, N., Benjamins, J.-W., Morley, M.P., van de Vegte, Y. J., Teumer, A., Trenkwalder, T., Reinhard, W., Cappola, T.P., and van der Harst, P. (2020). The genetic makeup of the electrocardiogram. Cell Syst. *11*, 229–238.e5. https://doi.org/10.1016/j.cels.2020.08.005.
4. Alipanahi, B., Hormozdiari, F., Behsaz, B., Cosentino, J., McCaw, Z.R., Schorsch, E., Sculley, D., Dorfman, E.H., Foster, P.J., Peng, L.H., et al. (2021). Large-scale machine-learning-based phenotyping significantly improves genomic discovery for optic nerve head morphology. Am. J. Hum. Genet. *108*, 1217–1230. https://doi.org/10.1016/j.ajhg.2021.05.004.
5. Aung, N., Vargas, J.D., Yang, C., Fung, K., Sanghvi, M.M., Piechnik, S.K., Neubauer, S., Manichaikul, A., Rotter, J.I., Taylor, K.D., et al. (2022). Genome-wide association analysis reveals insights into the genetic architecture of right ventricular structure and function. Nat. Genet. *54*, 783–791. https://doi.org/10.1038/s41588-022-01083-2.
6. Pirruccello, J.P., Di Achille, P., Nauffal, V., Nekoui, M., Friedman, S.F., Klarqvist, M.D.R., Chaffin, M.D., Weng, L.-C., Cunningham, J.W., Khurshid, S., et al. (2022). Genetic analysis of right heart structure and function in 40,000 people. Nat. Genet. *54*, 792–803. https://doi.org/10.1038/s41588-022-01090-3.
7. Cosentino, J., Behsaz, B., Alipanahi, B., McCaw, Z.R., Hill, D., Schwantes-An, T.-H., Lai, D., Carroll, A., Hobbs, B.D., Cho, M. H., et al. (2023). Inference of chronic obstructive pulmonary disease with deep learning on raw spirograms identifies new genetic loci and improves risk models. Nat. Genet. *55*, 787–795. https://doi.org/10.1038/s41588-023-01372-4.
8. Yun, T., Cosentino, J., Behsaz, B., McCaw, Z.R., Hill, D., Luben, R., Lai, D., Bates, J., Yang, H., Schwantes-An, T.-H., et al. (2024). Unsupervised representation learning on high-dimensional clinical data improves genomic discovery and prediction. Nat. Genet. *56*, 1604–1613. https://doi.org/10.1038/s41588-024-01831-6.
9. Gomes, B., Singh, A., O'Sullivan, J., Schnurr, T.M., Goddard, P.C., Loong, S., Amar, D., Hughes, J.W., Kostur, M., Haddad, F., et al. (2024). Genetic architecture of cardiac dynamic flow volumes. Nat. Genet. *56*, 245–257. https://doi.org/10.1038/s41588-023-01587-5.
10. Acosta, J.N., Falcone, G.J., Rajpurkar, P., and Topol, E.J. (2022). Multimodal biomedical ai. Nat. Med. *28*, 1773–1784. https://doi.org/10.1038/s41591-022-01981-2.
11. Kline, A., Wang, H., Li, Y., Dennis, S., Hutch, M., Xu, Z., Wang, F., Cheng, F., and Luo, Y. (2022). Multimodal machine learning in precision health: A scoping review. npj Digit. Med. *5*, 171. https://doi.org/10.1038/s41746-022-00712-8.
12. Soenksen, L.R., Ma, Y., Zeng, C., Boussioux, L., Villalobos Carballo, K., Na, L., Wiberg, H.M., Li, M.L., Fuentes, I., and Bertsimas, D. (2022). Integrated multimodal artificial intelligence framework for healthcare applications. npj Digit. Med. *5*, 149. https://doi.org/10.1038/s41746-022-00689-4.
13. Tomašev, N., Glorot, X., Rae, J.W., Zielinski, M., Askham, H., Saraiva, A., Mottram, A., Meyer, C., Ravuri, S., Protsyuk, I., et al. (2019). A clinically applicable approach to continuous prediction of future acute kidney injury. Nature *572*, 116–119. https://doi.org/10.1038/s41586-019-1390-1.
14. Reyes, D.M., Chao, H., Hahn, J., Shen, L., and Yan, P. (2024). Multimodal neurodegenerative disease subtyping explained by chatgpt. Preprint at arXiv. https://doi.org/10.48550/ARXIV.2402.00137.
15. Yang, Z., Wen, J., Abdulkadir, A., Cui, Y., Erus, G., Mamourian, E., Melhem, R., Srinivasan, D., Govindarajan, S.T., Chen, J., et al. (2024). Gene-sgan: discovering disease subtypes with imaging and genetic signatures via multi-view weakly-supervised deep clustering. Nat. Commun. *15*, 354. https://doi.org/10.1038/s41467-023-44271-2.
16. Mei, X., Lee, H.-C., Diao, K.-y., Huang, M., Lin, B., Liu, C., Xie, Z., Ma, Y., Robson, P.M., Chung, M., et al. (2020). Artificial intelligence–enabled rapid diagnosis of patients with covid-19. Nat. Med. *26*, 1224–1228. https://doi.org/10.1038/s41591-020-0931-3.
17. Yang, L., Xu, S., Sellergren, A., Kohlberger, T., Zhou, Y., Ktena, I., Kiraly, A., Ahmed, F., Hormozdiari, F., Jaroensri, T., et al. (2024). Advancing multimodal medical capabilities of gemini. Preprint at arXiv. https://doi.org/10.48550/ARXIV.2405.03162.
18. Wu, C., Zhang, X., Zhang, Y., Wang, Y., and Xie, W. (2023). Towards generalist foundation model for radiology by leveraging web-scale 2d and 3d medical data. Preprint at arXiv. https://doi.org/10.48550/ARXIV.2308.02463.
19. Zhang, K., Yu, J., Adhikarla, E., Zhou, R., Yan, Z., Liu, Y., Liu, Z., He, L., Davison, B., Li, X., et al. (2023). Biomedgpt: A unified and generalist biomedical generative pre-trained transformer for vision, language, and multimodal tasks. Preprint at arXiv. https://doi.org/10.48550/ARXIV.2305.17100.
20. Belyaeva, A., Cosentino, J., Hormozdiari, F., Eswaran, K., Shetty, S., Corrado, G., Carroll, A., McLean, C.Y., and Furlotte, N.A. (2023). Multimodal llms for health grounded in individual-specific data. In Machine Learning for Multimodal Healthcare Data (Springer Nature Switzerland), pp. 86–102. https://doi.org/10.1007/978-3-031-47679-2_7.
21. Peterson, V.M., Zhang, K.X., Kumar, N., Wong, J., Li, L., Wilson, D.C., Moore, R., McClanahan, T.K., Sadekova, S., and Klappenbach, J.A. (2017). Multiplexed quantification of proteins and transcripts in single cells. Nat. Biotechnol. *35*, 936–939. https://doi.org/10.1038/nbt.3973.
22. Mimitou, E.P., Cheng, A., Montalbano, A., Hao, S., Stoeckius, M., Legut, M., Roush, T., Herrera, A., Papalexi, E., Ouyang, Z., et al. (2019). Multiplexed detection of proteins, transcriptomes, clonotypes and crispr perturbations in single cells. Nat. Methods *16*, 409–412. https://doi.org/10.1038/s41592-019-0392-0.
23. Mimitou, E.P., Lareau, C.A., Chen, K.Y., Zorzetto-Fernandes, A.L., Hao, Y., Takeshima, Y., Luo, W., Huang, T.-S., Yeung, B. Z., Papalexi, E., et al. (2021). Scalable, multimodal profiling of chromatin accessibility, gene expression and protein levels in single cells. Nat. Biotechnol. *39*, 1246–1258. https://doi.org/10.1038/s41587-021-00927-2.
24. Hao, Y., Hao, S., Andersen-Nissen, E., Mauck, W.M., Zheng, S., Butler, A., Lee, M.J., Wilk, A.J., Darby, C., Zager, M., et al. (2021). Integrated analysis of multimodal single-cell data. Cell *184*, 3573–3587.e29. https://doi.org/10.1016/j.cell.2021.04.048.
25. Kuchroo, M., Huang, J., Wong, P., Grenier, J.-C., Shung, D., Tong, A., Lucas, C., Klein, J., Burkhardt, D.B., Gigante, S.,

et al. (2022). Multiscale phate identifies multimodal signatures of covid-19. Nat. Biotechnol. *40*, 681–691. https://doi.org/10.1038/s41587-021-01186-x.
26. Friedman, S.F., Moran, G.E., Rakic, M., and Phillipakis, A. (2024). Genetic architectures of medical images revealed by registration of multiple modalities. Bioinf. Biol. Insights *18*, 11779322241282489. https://doi.org/10.1177/11779322241282489.
27. Radhakrishnan, A., Friedman, S.F., Khurshid, S., Ng, K., Batra, P., Lubitz, S.A., Philippakis, A.A., and Uhler, C. (2023). Cross-modal autoencoder framework learns holistic representations of cardiovascular state. Nat. Commun. *14*, 2436. https://doi.org/10.1038/s41467-023-38125-0.
28. Kingma, D.P., and Welling, M. (2013). Auto-encoding variational bayes. Preprint at arXiv. https://doi.org/10.48550/ARXIV.1312.6114.
29. Aschard, H., Vilhjálmsson, B.J., Greliche, N., Morange, P.-E., Trégouët, D.-A., and Kraft, P. (2014). Maximizing the power of principal-component analysis of correlated phenotypes in genome-wide association studies. Am. J. Hum. Genet. *94*, 662–676. https://doi.org/10.1016/j.ajhg.2014.03.016.
30. Porter, H.F., and O'Reilly, P.F. (2017). Multivariate simulation framework reveals performance of multi-trait gwas methods. Sci. Rep. *7*, 38837. https://doi.org/10.1038/srep38837.
31. Rumelhart, D.E., Hinton, G.E., and Williams, R.J. (1986). Learning internal representations by error propagation. In Parallel Distributed Processing: Explorations in the Microstructure of Cognition, *1* (Cambridge, MA, USA: MIT Press). https://doi.org/10.7551/mitpress/5236.001.0001.
32. Mbatchou, J., Barnard, L., Backman, J., Marcketta, A., Kosmicki, J.A., Ziyatdinov, A., Benner, C., O'Dushlaine, C., Barber, M., Boutkov, B., et al. (2021). Computationally efficient whole-genome regression for quantitative and binary traits. Nat. Genet. *53*, 1097–1103. https://doi.org/10.1038/s41588-021-00870-7.
33. McLean, C.Y., Bristor, D., Hiller, M., Clarke, S.L., Schaar, B.T., Lowe, C.B., Wenger, A.M., and Bejerano, G. (2010). GREAT improves functional interpretation of cis-regulatory regions. Nat. Biotechnol. *28*, 495–501. https://doi.org/10.1038/nbt.1630.
34. Day, N., Oakes, S., Luben, R., Khaw, K.T., Bingham, S., Welch, A., and Wareham, N. (1999). EPIC-Norfolk: study design and characteristics of the cohort. european prospective investigation of cancer. Br. J. Cancer *80*, 95–103. https://doi.org/10.17863/CAM.89388.
35. Lai, D., Schwantes-An, T.-H., Abreu, M., Chan, G., Hesselbrock, V., Kamarajan, C., Liu, Y., Meyers, J.L., Nurnberger, J.I., Plawecki, M.H., et al. (2022). Gene-based polygenic risk scores analysis of alcohol use disorder in african americans. Transl. Psychiatry *12*, 266. https://doi.org/10.1038/s41398-022-02029-2.
36. Price, A.L., Patterson, N.J., Plenge, R.M., Weinblatt, M.E., Shadick, N.A., and Reich, D. (2006). Principal components analysis corrects for stratification in genome-wide association studies. Nat. Genet. *38*, 904–909. https://doi.org/10.1038/ng1847.
37. Auton, A., Abecasis, G.R., Altshuler, D.M., Durbin, R.M., Abecasis, G.R., Bentley, D.R., Chakravarti, A., Clark, A.G., Donnelly, P., Eichler, E.E., et al. (2015). A global reference for human genetic variation. Nature *526*, 68–74. https://doi.org/10.1038/nature15393.
38. Das, S., Forer, L., Schönherr, S., Sidore, C., Locke, A.E., Kwong, A., Vrieze, S.I., Chew, E.Y., Levy, S., McGue, M., et al. (2016). Next-generation genotype imputation service and methods. Nat. Genet. *48*, 1284–1287. https://doi.org/10.1038/ng.3656.
39. Andrew, G., Arora, R., Bilmes, J., and Livescu, K. (2013). Deep canonical correlation analysis. In Proceedings of the 30th International Conference on Machine Learning volume 28 of Proceedings of Machine Learning Research, S. Dasgupta and D. McAllester, eds. (Atlanta, Georgia, USA: PMLR), pp. 1247–1255.
40. Yang, H., Zeng, Y., Chen, W., Sun, Y., Hu, Y., Ying, Z., Wang, J., Qu, Y., Fang, F., Valdimarsdóttir, U.A., and Song, H. (2022). The role of genetic predisposition in cardiovascular risk after cancer diagnosis: a matched cohort study of the uk biobank. Br. J. Cancer *127*, 1650–1659. https://doi.org/10.1038/s41416-022-01935-y.
41. Rentsch, C.T., Garfield, V., Mathur, R., Eastwood, S.V., Smeeth, L., Chaturvedi, N., and Bhaskaran, K. (2023). Sex-specific risks for cardiovascular disease across the glycaemic spectrum: a population-based cohort study using the uk biobank. Lancet Reg. Health. Eur. *32*, 100693. https://doi.org/10.1016/j.lanepe.2023.100693.
42. Ghoussaini, M., Mountjoy, E., Carmona, M., Peat, G., Schmidt, E.M., Hercules, A., Fumis, L., Miranda, A., Carvalho-Silva, D., Buniello, A., et al. (2021). Open targets genetics: systematic identification of trait-associated genes using large-scale genetics and functional genomics. Nucleic Acids Res. *49*, D1311–D1320. https://doi.org/10.1093/nar/gkaa840.
43. Mountjoy, E., Schmidt, E.M., Carmona, M., Schwartzentruber, J., Peat, G., Miranda, A., Fumis, L., Hayhurst, J., Buniello, A., Karim, M.A., et al. (2021). An open approach to systematically prioritize causal variants and genes at all published human gwas trait-associated loci. Nat. Genet. *53*, 1527–1533. https://doi.org/10.1038/s41588-021-00945-5.
44. Lawlor, D.A., Ebrahim, S., Davey Smith, G.; and British women's heart and health study (2002). Socioeconomic position in childhood and adulthood and insulin resistance: cross sectional survey using data from british women's heart and health study. BMJ *325*, 805. https://doi.org/10.1136/bmj.325.7368.805.
45. Lawlor, D.A., Patel, R., and Ebrahim, S. (2003). Association between falls in elderly women and chronic diseases and drug use: cross sectional study. BMJ *327*, 712–717. https://doi.org/10.1136/bmj.327.7417.712.
46. Kim, L.G., Carson, C., Lawlor, D.A., and Ebrahim, S. (2010). Geographical variation in cardiovascular incidence: results from the british women's heart and health study. BMC Public Health *10*, 696. https://doi.org/10.1186/1471-2458-10-696.
47. Rasmussen, P.V., Nielsen, J.B., Skov, M.W., Pietersen, A., Graff, C., Lind, B., Struijk, J.J., Olesen, M.S., Haunsø, S., Køber, L., et al. (2017). Electrocardiographic pr interval duration and cardiovascular risk: Results from the copenhagen ecg study. Can. J. Cardiol. *33*, 674–681. https://doi.org/10.1016/j.cjca.2017.02.015.
48. van Setten, J., Brody, J.A., Jamshidi, Y., Swenson, B.R., Butler, A.M., Campbell, H., Del Greco, F.M., Evans, D.S., Gibson, Q., Gudbjartsson, D.F., et al. (2018). Pr interval genome-wide association meta-analysis identifies 50 loci associated with atrial and atrioventricular electrical activity. Nat. Commun. *9*, 2904. https://doi.org/10.1038/s41467-018-04766-9.

49. Ntalla, I., Weng, L.-C., Cartwright, J.H., Hall, A.W., Sveinbjornsson, G., Tucker, N.R., Choi, S.H., Chaffin, M.D., Roselli, C., Barnes, M.R., et al. (2020). Multi-ancestry gwas of the electrocardiographic pr interval identifies 202 loci underlying cardiac conduction. Nat. Commun. *11*, 2542. https://doi.org/10.1038/s41467-020-15706-x.
50. Ward, D.E. (1988). Prolongation of the qt interval as an indicator of risk of a cardiac event. Eur. Heart J. *9*, 139–144. https://doi.org/10.1093/eurheartj/9.suppl_g.139.
51. Robbins, J., Nelson, J.C., Rautaharju, P.M., and Gottdiener, J. S. (2003). The association between the length of the qt interval and mortality in the cardiovascular health study. Am. J. Med. *115*, 689–694. https://doi.org/10.1016/j.amjmed.2003.07.014.
52. Magnani, J.W., Gorodeski, E.Z., Johnson, V.M., Sullivan, L. M., Hamburg, N.M., Benjamin, E.J., and Ellinor, P.T. (2011). P wave duration is associated with cardiovascular and all-cause mortality outcomes: the national health and nutrition examination survey. Heart Rhythm *8*, 93–100. https://doi.org/10.1016/j.hrthm.2010.09.020.
53. Magnani, J.W., Zhu, L., Lopez, F., Pencina, M.J., Agarwal, S. K., Soliman, E.Z., Benjamin, E.J., and Alonso, A. (2015). P-wave indices and atrial fibrillation: Cross-cohort assessments from the framingham heart study (fhs) and atherosclerosis risk in communities (aric) study. Am. Heart J. *169*, 53–61.e1. https://doi.org/10.1016/j.ahj.2014.10.009.
54. Nielsen, J.B., Kühl, J.T., Pietersen, A., Graff, C., Lind, B., Struijk, J.J., Olesen, M.S., Sinner, M.F., Bachmann, T.N., Haunsø, S., et al. (2015). P-wave duration and the risk of atrial fibrillation: Results from the copenhagen ecg study. Heart Rhythm *12*, 1887–1895. https://doi.org/10.1016/j.hrthm.2015.04.026.
55. Christophersen, I.E., Magnani, J.W., Yin, X., Barnard, J., Weng, L.-C., Arking, D.E., Niemeijer, M.N., Lubitz, S.A., Avery, C.L., Duan, Q., et al. (2017). Fifteen genetic loci associated with the electrocardiographic p wave. Circ. Cardiovasc. Genet. *10*, e001667. https://doi.org/10.1161/circgenetics.116.001667.
56. Das, M.K., Maskoun, W., Shen, C., Michael, M.A., Suradi, H., Desai, M., Subbarao, R., and Bhakta, D. (2010). Fragmented qrs on twelve-lead electrocardiogram predicts arrhythmic events in patients with ischemic and nonischemic cardiomyopathy. Heart Rhythm *7*, 74–80. https://doi.org/10.1016/j.hrthm.2009.09.065.
57. Kurl, S., Mäkikallio, T.H., Rautaharju, P., Kiviniemi, V., and Laukkanen, J.A. (2012). Duration of qrs complex in resting electrocardiogram is a predictor of sudden cardiac death in men. Circulation *125*, 2588–2594. https://doi.org/10.1161/circulationaha.111.025577.
58. Young, W.J., Haessler, J., Benjamins, J.-W., Repetto, L., Yao, J., Isaacs, A., Harper, A.R., Ramirez, J., Garnier, S., van Duijvenboden, S., et al. (2023). Genetic architecture of spatial electrical biomarkers for cardiac arrhythmia and relationship with cardiovascular disease. Nat. Commun. *14*, 1411. https://doi.org/10.1038/s41467-023-36997-w.
59. Kolder, I.C.R.M., Tanck, M.W.T., and Bezzina, C.R. (2012). Common genetic variation modulating cardiac ecg parameters and susceptibility to sudden cardiac death. J. Mol. Cell. Cardiol. *52*, 620–629. https://doi.org/10.1016/j.yjmcc.2011.12.014.
60. Locatello, F., Bauer, S., Lucic, M., Raetsch, G., Gelly, S., Schölkopf, B., and Bachem, O. (2019). Challenging common assumptions in the unsupervised learning of disentangled representations. In Proceedings of the 36th International Conference on Machine Learning volume 97 of Proceedings of Machine Learning Research, K. Chaudhuri and R. Salakhutdinov, eds. (PMLR), pp. 4114–4124. https://doi.org/10.48550/ARXIV.1811.12359.

The American Journal of Human Genetics, Volume *112*

Supplemental information

Applying multimodal AI to physiological waveforms improves genetic prediction of cardiovascular traits

Yuchen Zhou, Justin Khasentino, Taedong Yun, Mahantesh I. Biradar, Jacqueline Shreibati, Dongbing Lai, Tae-Hwi Schwantes-An, Robert Luben, Zachary R. McCaw, Jorgen Engmann, Rui Providencia, Amand Floriaan Schmidt, Patricia B. Munroe, Howard Yang, Andrew Carroll, Anthony P. Khawaja, Cory Y. McLean, Babak Behsaz, and Farhad Hormozdiari

Supplemental Notes

Dataset acknowledgment

We are grateful to all the participants who have been part of the project and to the many members of the study teams at the University of Cambridge who have enabled this research. Indiana Biobank was made possible, in part, with support from the Indiana Clinical and Translational Sciences Institute funded, in part by Award Number UL1TR002529 from the National Institutes of Health, National Center for Advancing Translational Sciences, Clinical and Translational Sciences Award, and the National Center for Research Resources, Construction grant number RR020128 and the Lilly Endowment. The content is solely the responsibility of the authors and does not necessarily represent the official views of the National Institutes of Health. The authors acknowledge the Indiana University Pervasive Technology Institute for providing [HPC (Big Red II, Karst, Carbonate), visualization, database, storage, or consulting] resources that have contributed to the research results reported within this paper. BWHHS is supported by funding from the British Heart Foundation and the Department of Health Policy Research Programme (England).

M-REGLE model architecture (ECG + PPG)

Encoder

Layer (type)	Output Shape	Param #
vae_encoder_input (InputLayer)	[(None, 700, 1)]	0
vae_encoder_conv1 (Conv1D)	(None, 700, 16)	128
leaky_re_lu (LeakyReLU)	(None, 700, 16)	0
vae_encoder_pooling1 (MaxPooling1D)	(None, 350, 16)	0
vae_encoder_conv2 (Conv1D)	(None, 350, 16)	1808
leaky_re_lu_1 (LeakyReLU)	(None, 350, 16)	0
vae_encoder_pooling2 (MaxPooling1D)	(None, 175, 16)	0
vae_encoder_conv3 (Conv1D)	(None, 175, 16)	1808
leaky_re_lu_2 (LeakyReLU)	(None, 175, 16)	0
vae_encoder_pooling3 (MaxPooling1D)	(None, 88, 16)	0
vae_encoder_conv4 (Conv1D)	(None, 88, 16)	1808
leaky_re_lu_3 (LeakyReLU)	(None, 88, 16)	0
vae_encoder_pooling4 (MaxPooling1D)	(None, 44, 16)	0
vae_encoder_conv5 (Conv1D)	(None, 44, 16)	1808
leaky_re_lu_4 (LeakyReLU)	(None, 44, 16)	0
vae_encoder_pooling5 (MaxPooling1D)	(None, 22, 16)	0
vae_encoder_flatten (Flatten)	(None, 352)	0
vae_encoder_dense1 (Dense)	(None, 64)	22592
vae_encoder_dense2 (Dense)	(None, 32)	2080
z_mean (Dense)	(None, 12)	396
z_log_var (Dense)	(None, 12)	396
gaussian_sampling (GaussianSampling)	(None, 12)	0

Total params: 32824

Trainable params: 32824

Decoder

Layer (type)	Output Shape	Param #
decoder_input (InputLayer)	[(None, 12)]	0
decoder_dense1 (Dense)	(None, 32)	416
decoder_dense2 (Dense)	(None, 64)	2112
decoder_dense3 (Dense)	(None, 352)	22880
reshape (Reshape)	(None, 22, 16)	0
decoder_upsample1 (UpSampling1D)	(None, 44, 16)	0
decoder_trans_conv1 (Conv1DTranspose)	(None, 44, 16)	1808
leaky_re_lu_5 (LeakyReLU)	(None, 44, 16)	0
decoder_upsample2 (UpSampling1D)	(None, 88, 16)	0
decoder_trans_conv2 (Conv1DTranspose)	(None, 88, 16)	1808
leaky_re_lu_6 (LeakyReLU)	(None, 88, 16)	0
decoder_upsample3 (UpSampling1D)	(None, 176, 16)	0
decoder_trans_conv3 (Conv1DTranspose)	(None, 176, 16)	1808
leaky_re_lu_7 (LeakyReLU)	(None, 176, 16)	0
decoder_upsample4 (UpSampling1D)	(None, 352, 16)	0
decoder_trans_conv4 (Conv1DTranspose)	(None, 352, 16)	1808
leaky_re_lu_8 (LeakyReLU)	(None, 352, 16)	0
decoder_upsample5 (UpSampling1D)	(None, 704, 16)	0
decoder_trans_conv5 (Conv1DTranspose)	(None, 704, 1)	113
leaky_re_lu_9 (LeakyReLU)	(None, 704, 1)	0
cropping1d (Cropping1D)	(None, 700, 1)	0

Total params: 32753

Trainable params: 32753

M-REGLE model architecture (12 Lead ECG)

Encoder

Layer (type)	Output Shape	Param #
vae_encoder_input (InputLayer)	[(None, 600, 12)]	0
vae_encoder_conv1 (Conv1D)	(None, 600, 32)	1184
leaky_re_lu (LeakyReLU)	(None, 600, 32)	0
vae_encoder_pooling1 (MaxPooling1D)	(None, 300, 32)	0
vae_encoder_conv2 (Conv1D)	(None, 300, 32)	3104
leaky_re_lu_1 (LeakyReLU)	(None, 300, 32)	0
vae_encoder_pooling2 (MaxPooling1D)	(None, 150, 32)	0
vae_encoder_conv3 (Conv1D)	(None, 150, 32)	3104
leaky_re_lu_2 (LeakyReLU)	(None, 150, 32)	0
vae_encoder_pooling3 (MaxPooling1D)	(None, 75, 32)	0
vae_encoder_flatten (Flatten)	(None, 2400)	0
vae_encoder_dense1 (Dense)	(None, 512)	1229312
z_mean (Dense)	(None, 96)	49248
z_log_var (Dense)	(None, 96)	49248
gaussian_sampling (GaussianSampling)	(None, 96)	0

Total params: 1335200

Trainable params: 1335200

Decoder

Layer (type)	Output Shape	Param #
decoder_input (InputLayer)	[(None, 96)]	0
decoder_dense1 (Dense)	(None, 512)	49664
decoder_dense2 (Dense)	(None, 2400)	1231200
reshape (Reshape)	(None, 75, 32)	0
decoder_upsample1 (UpSampling1D)	(None, 150, 32)	0
decoder_trans_conv1 (Conv1DTranspose)	(None, 150, 32)	3104
leaky_re_lu_3 (LeakyReLU)	(None, 150, 32)	0
decoder_upsample2 (UpSampling1D)	(None, 300, 32)	0
decoder_trans_conv2 (Conv1DTranspose)	(None, 300, 32)	3104
leaky_re_lu_4 (LeakyReLU)	(None, 300, 32)	0
decoder_upsample3 (UpSampling1D)	(None, 600, 32)	0
decoder_trans_conv3 (Conv1DTranspose)	(None, 600, 12)	1164
leaky_re_lu_5 (LeakyReLU)	(None, 600, 12)	0
cropping1d (Cropping1D)	(None, 600, 12)	0

Total params: 1288236

Trainable params: 1288236

Supplemental Figures

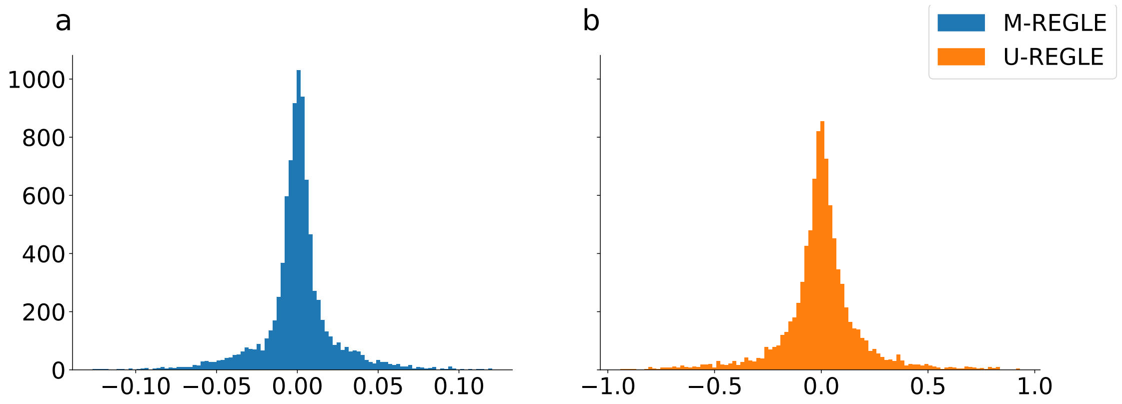

Figure S1: **Correlation coefficients (non-diagonal) histogram of M-REGLE and U-REGLE embeddings of 12-lead ECG.** a) Histogram of non-diagonal correlation coefficient matrices of M-REGLE 12-lead ECG embeddings. Most of the non-diagonal coefficients fall in [-0.1,0.1], which shows that the coordinates of M-REGLE embeddings are mostly orthogonalized. b) Histogram of non-diagonal correlation coefficient matrices of U-REGLE 12-lead ECG embeddings.

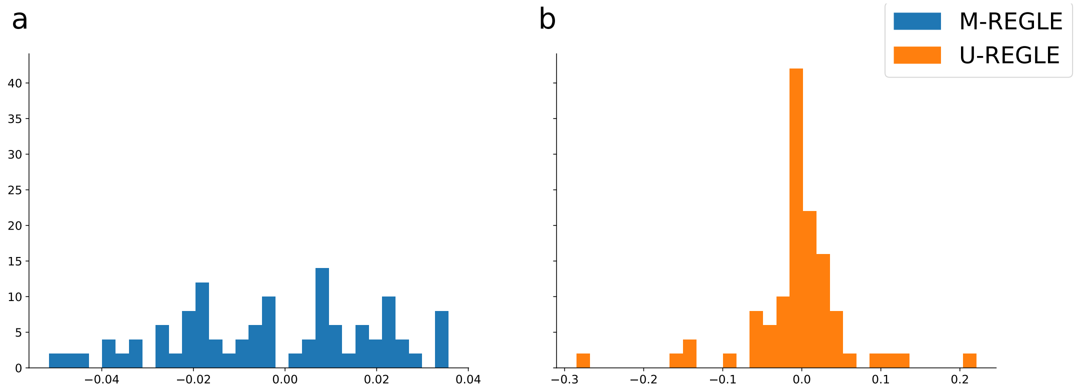

Figure S2: **Correlation coefficients (non-diagonal) histogram of M-REGLE and U-REGLE embeddings of ECG lead I + PPG.** a) Histogram of non-diagonal correlation coefficient matrices of M-REGLE ECG lead I + PPG embeddings. Most of the non-diagonal coefficients fall in [-0.05,0.05], which shows that the coordinates of M-REGLE embeddings are mostly orthogonalized. b) Histogram of non-diagonal correlation coefficient matrices of U-REGLE ECG lead I + PPG embeddings.

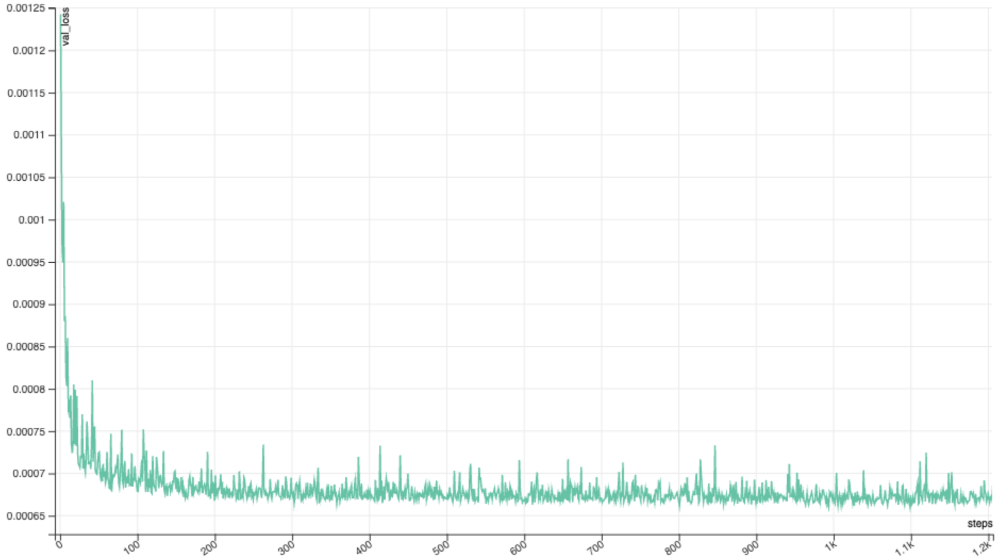

Figure S3: **Validation loss curve of training unimodal PPG model for up to 1200 epochs.** We trained the U-REGLE models for the same number of epochs with M-REGLE models and plotted the validation loss curve. The curve plateaued before 300 epochs which is the maximum number of epochs we used for U-REGLE models in this paper.

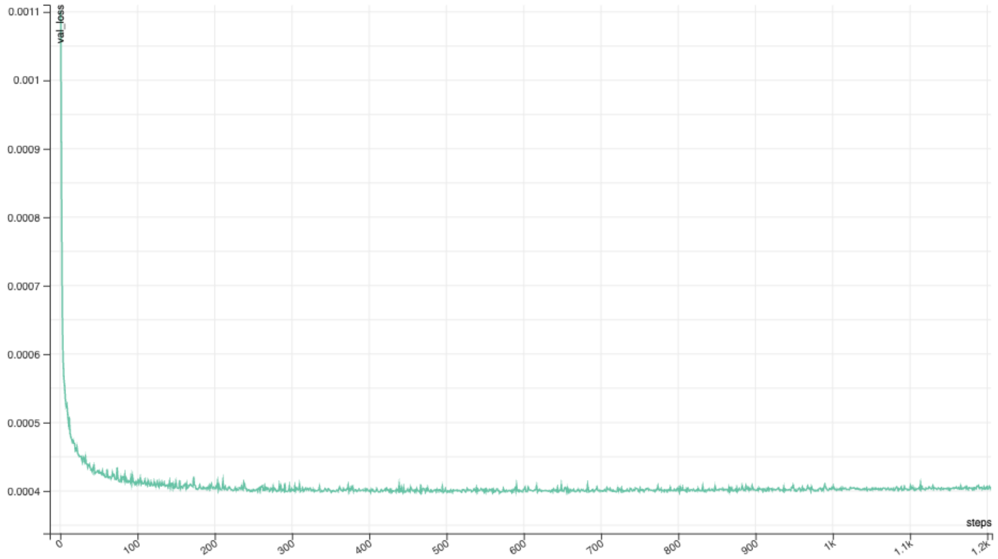

Figure S4: **Validation loss curve of training unimodal ECG lead I model for up to 1200 epochs.** See details in Figure S3.

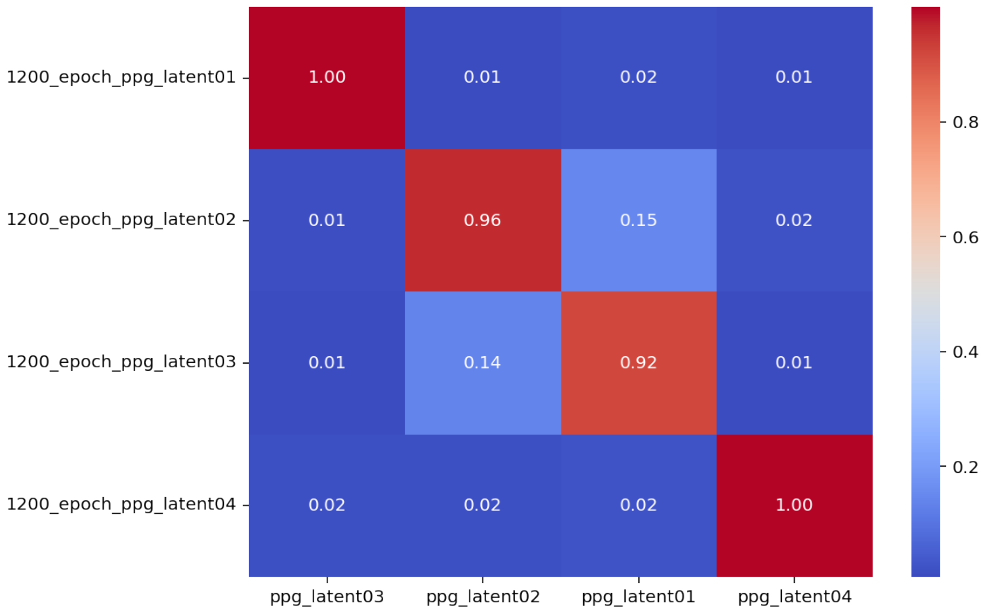

Figure S5: **Correlation coefficient matrix of the best unimodal PPG embeddings within 300 training epochs and the best unimodal PPG embeddings within 1200 training epochs.** To further show that the U-REGLE embeddings and downstream analyses remain stable when increasing the training epochs, we extracted the embeddings from the best model we got by training the model for up to 1200 epochs (see Figure S3), and compared them with the embeddings from the best model within 300 epochs (the ones we used in the paper). We got a correlation coefficient matrix close to an identity matrix, by reordering the coordinates of the embeddings and running Spearman correlation on the 1200-epoch embeddings and the 300-epoch embeddings.

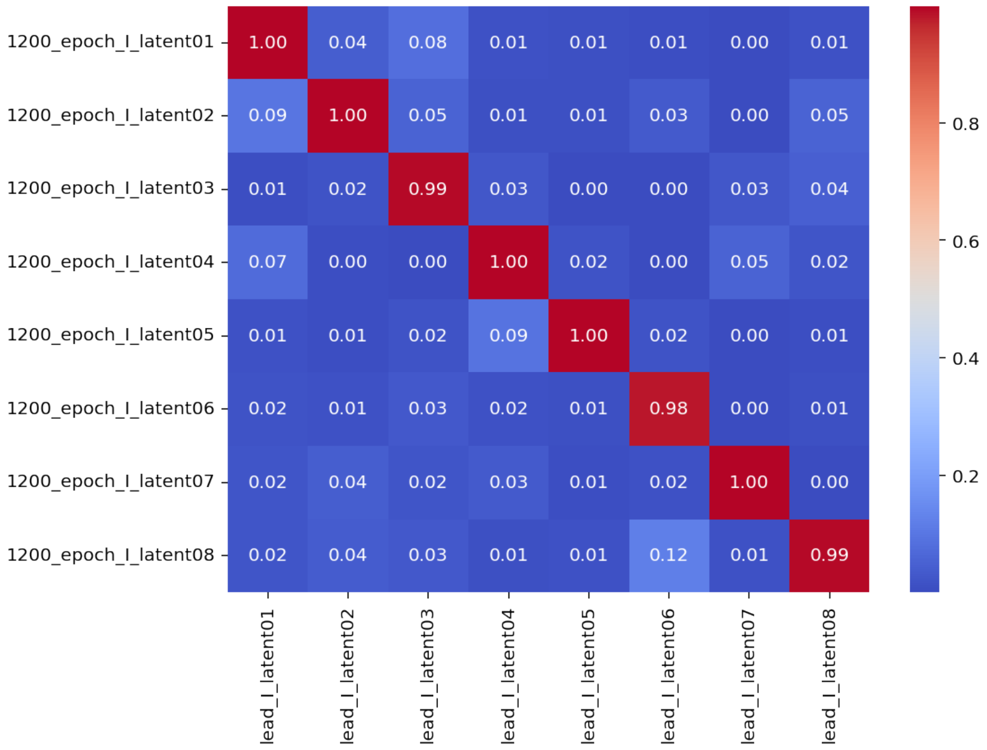

Figure S6: **Correlation coefficient matrix of the best unimodal ECG lead I embeddings within 300 training epochs and the best unimodal ECG lead I embeddings within 1200 training epochs.** See details in Figure S5)

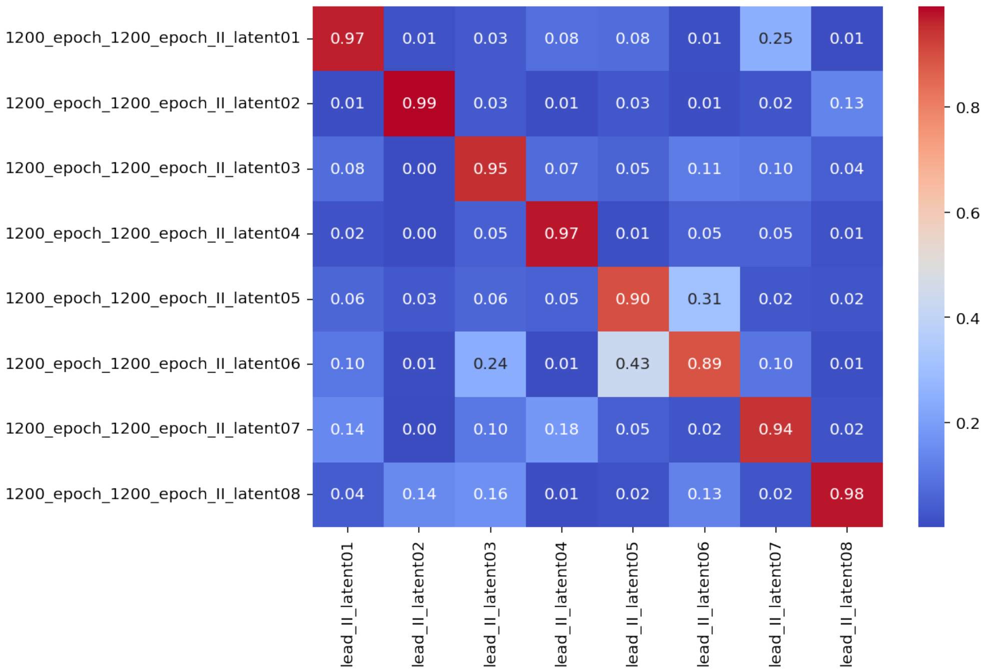

Figure S7: **Correlation coefficient matrix of the best unimodal ECG lead II embeddings within 300 training epochs and the best unimodal ECG lead II embeddings within 1200 training epochs.** See details in Figure S5)

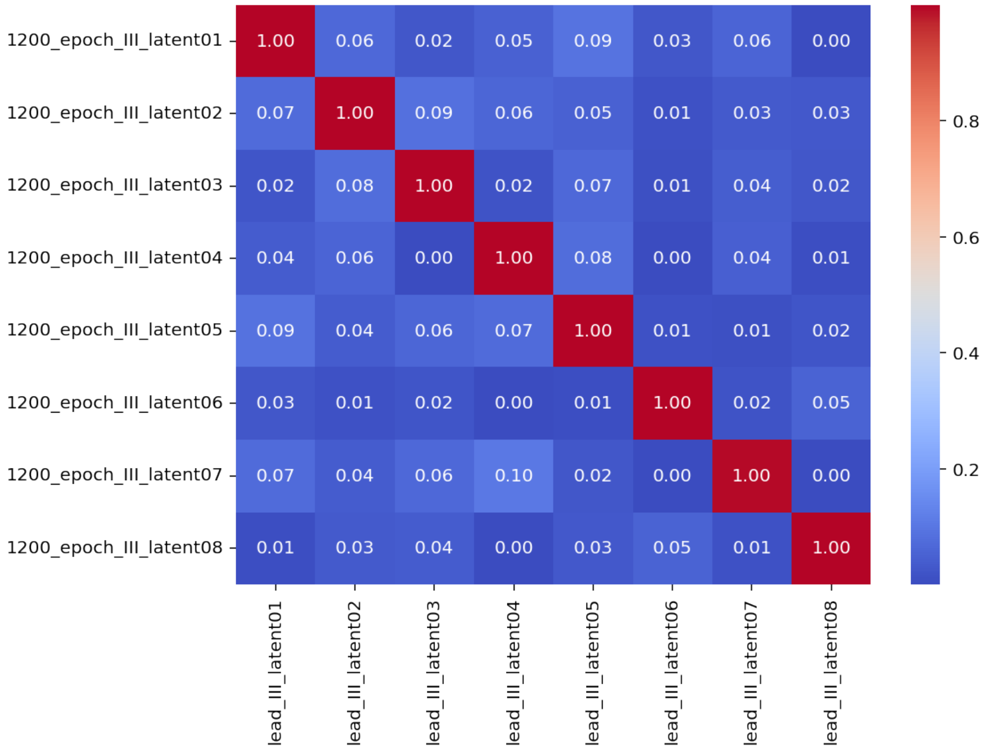

Figure S8: **Correlation coefficient matrix of the best unimodal ECG lead III embeddings within 300 training epochs and the best unimodal ECG lead III embeddings within 1200 training epochs.** See details in Figure S5)

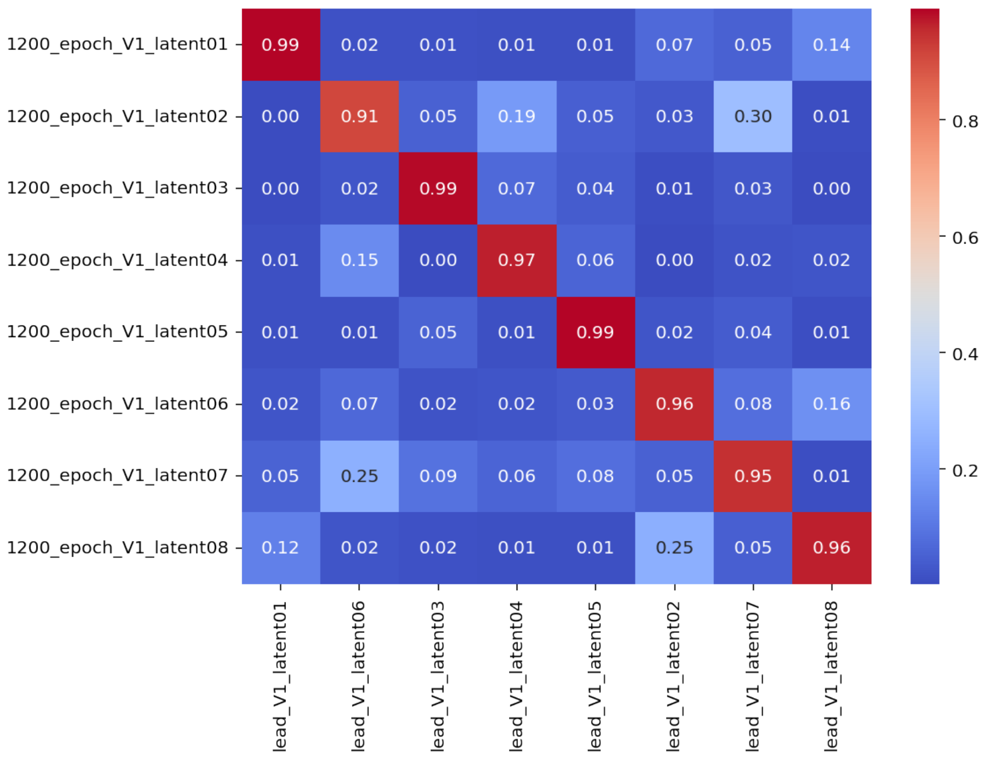

Figure S9: **Correlation coefficient matrix of the best unimodal ECG lead V1 embeddings within 300 training epochs and the best unimodal ECG lead V1 embeddings within 1200 training epochs.** See details in Figure S5)

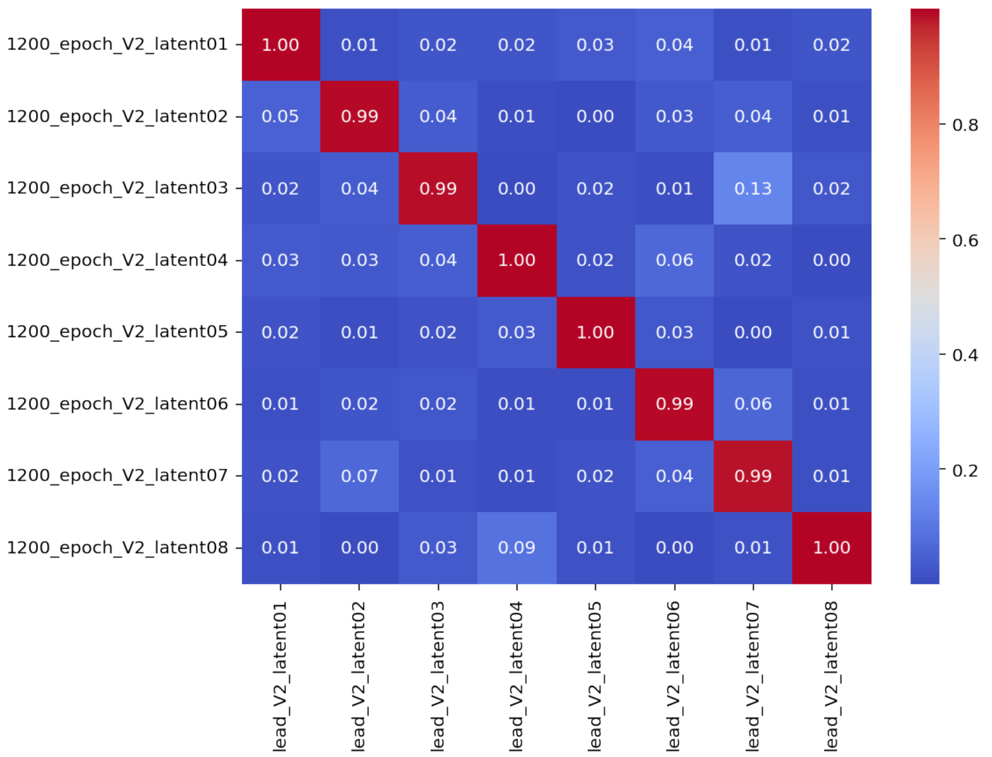

Figure S10: **Correlation coefficient matrix of the best unimodal ECG lead V2 embeddings within 300 training epochs and the best unimodal ECG lead V2 embeddings within 1200 training epochs.** See details in Figure S5)

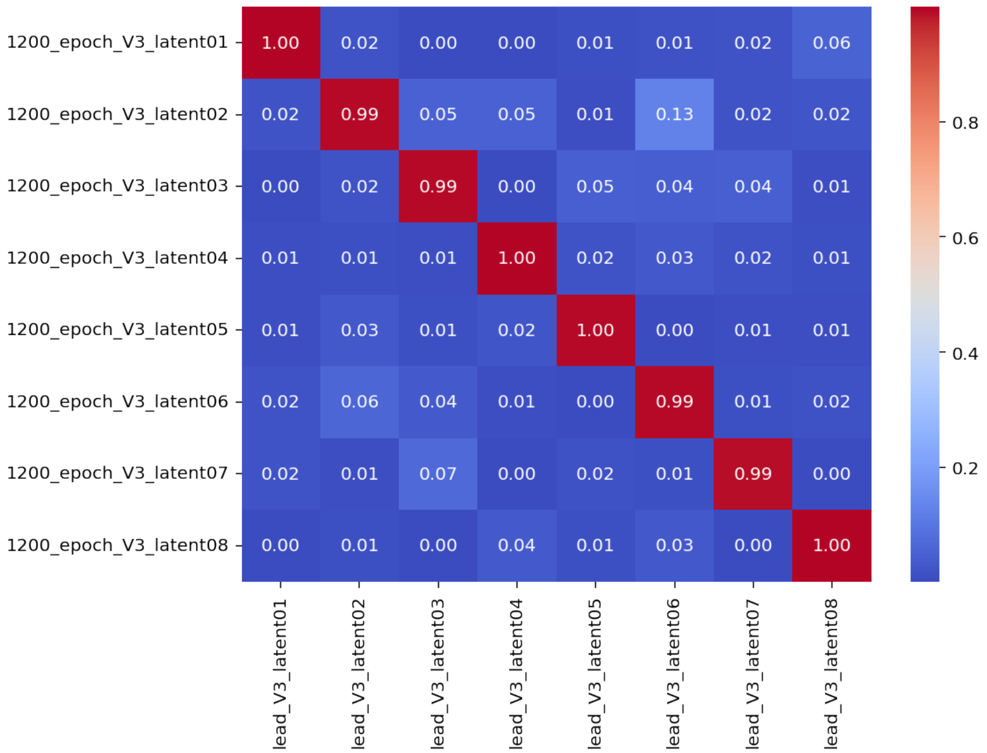

Figure S11: **Correlation coefficient matrix of the best unimodal ECG lead V3 embeddings within 300 training epochs and the best unimodal ECG lead V3 embeddings within 1200 training epochs.** See details in Figure S5)

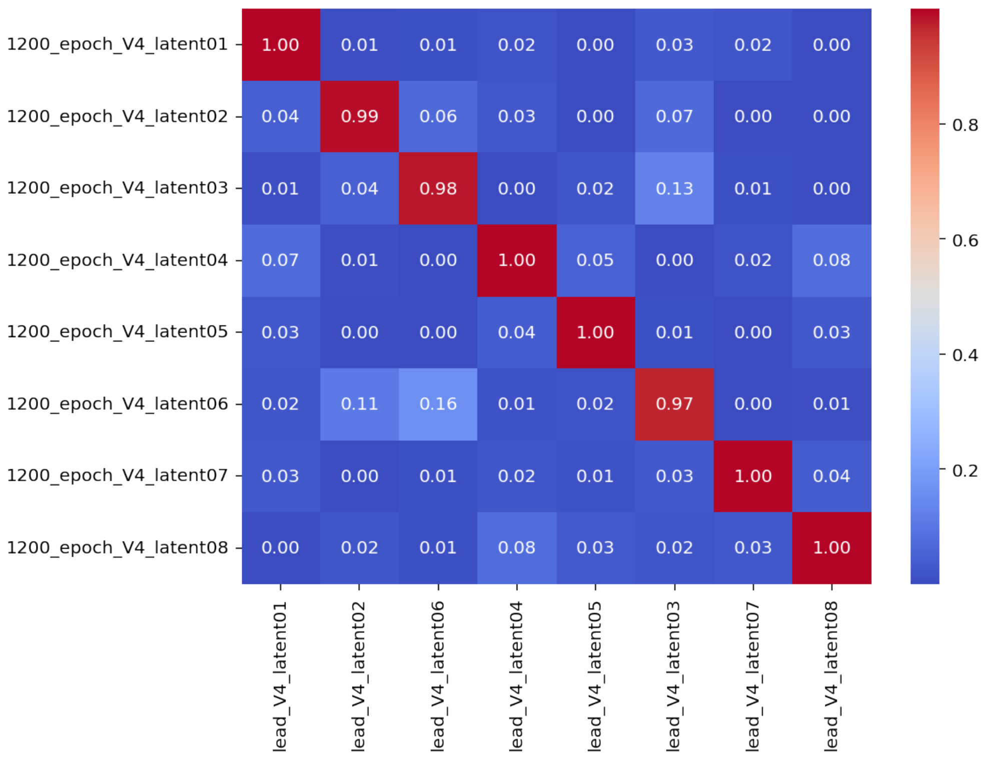

Figure S12: **Correlation coefficient matrix of the best unimodal ECG lead V4 embeddings within 300 training epochs and the best unimodal ECG lead V4 embeddings within 1200 training epochs.** See details in Figure S5)

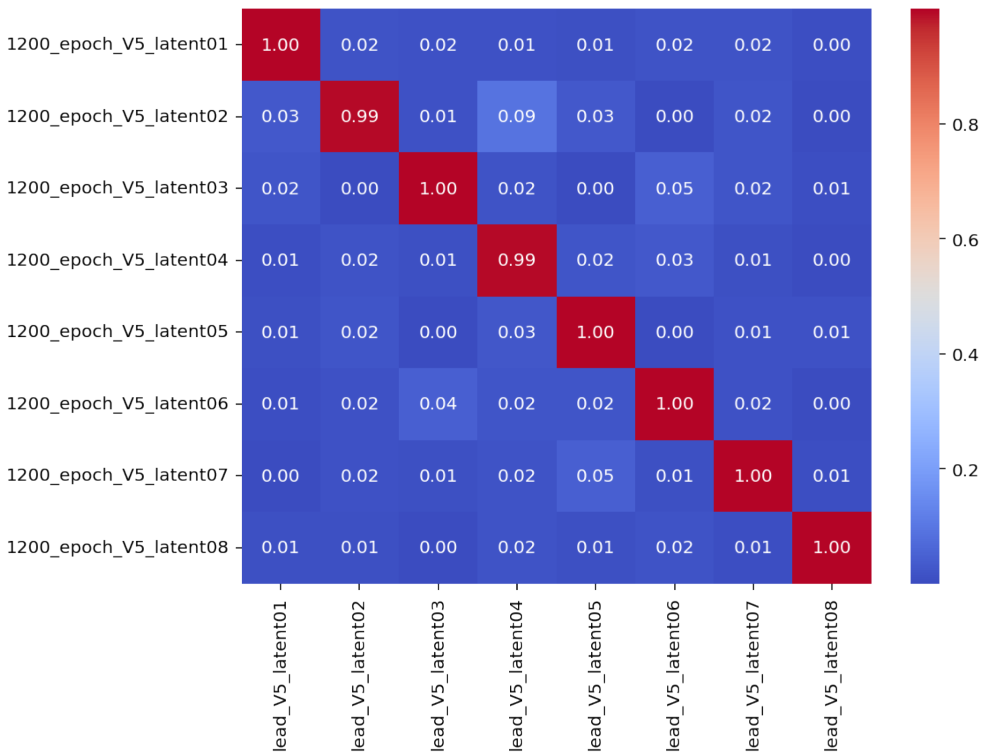

Figure S13: **Correlation coefficient matrix of the best unimodal ECG lead V5 embeddings within 300 training epochs and the best unimodal ECG lead V5 embeddings within 1200 training epochs.** See details in Figure S5)

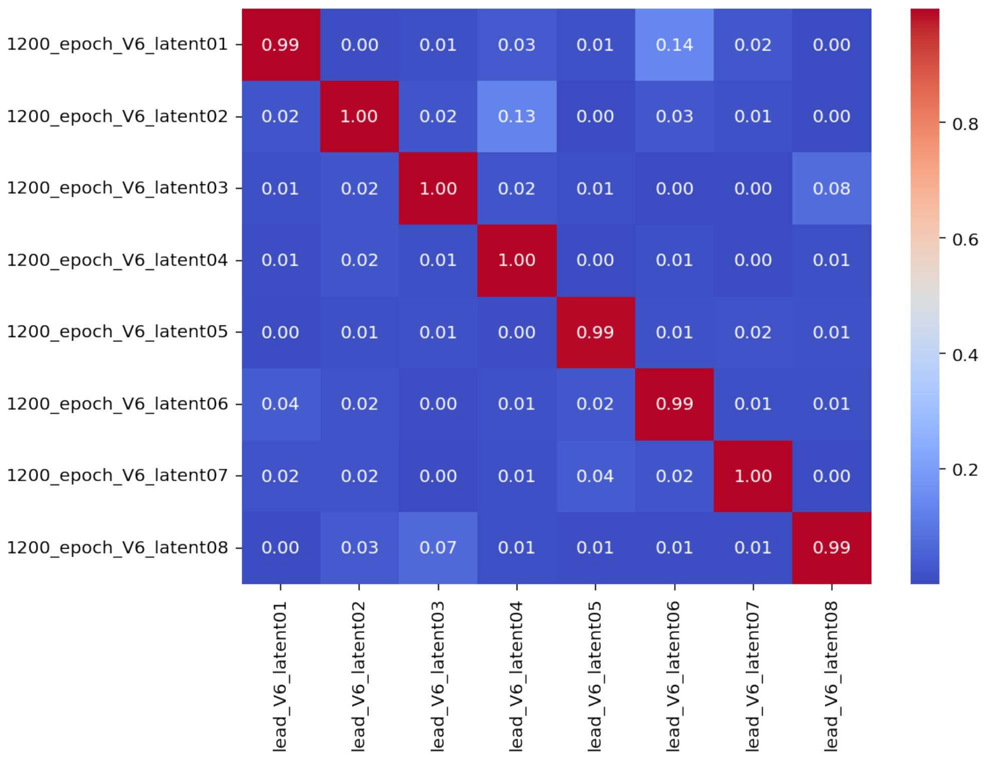

Figure S14: **Correlation coefficient matrix of the best unimodal ECG lead V6 embeddings within 300 training epochs and the best unimodal ECG lead V6 embeddings within 1200 training epochs.** See details in Figure S5)

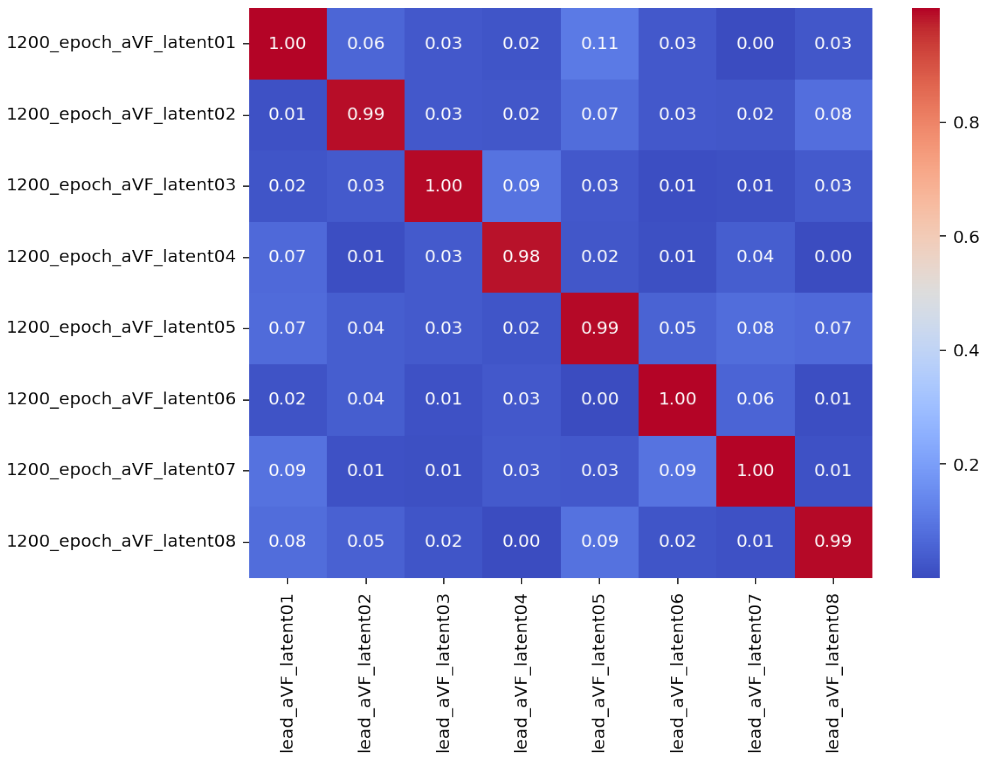

Figure S15: **Correlation coefficient matrix of the best unimodal ECG lead aVF embeddings within 300 training epochs and the best unimodal ECG lead aVF embeddings within 1200 training epochs.** See details in Figure S5)

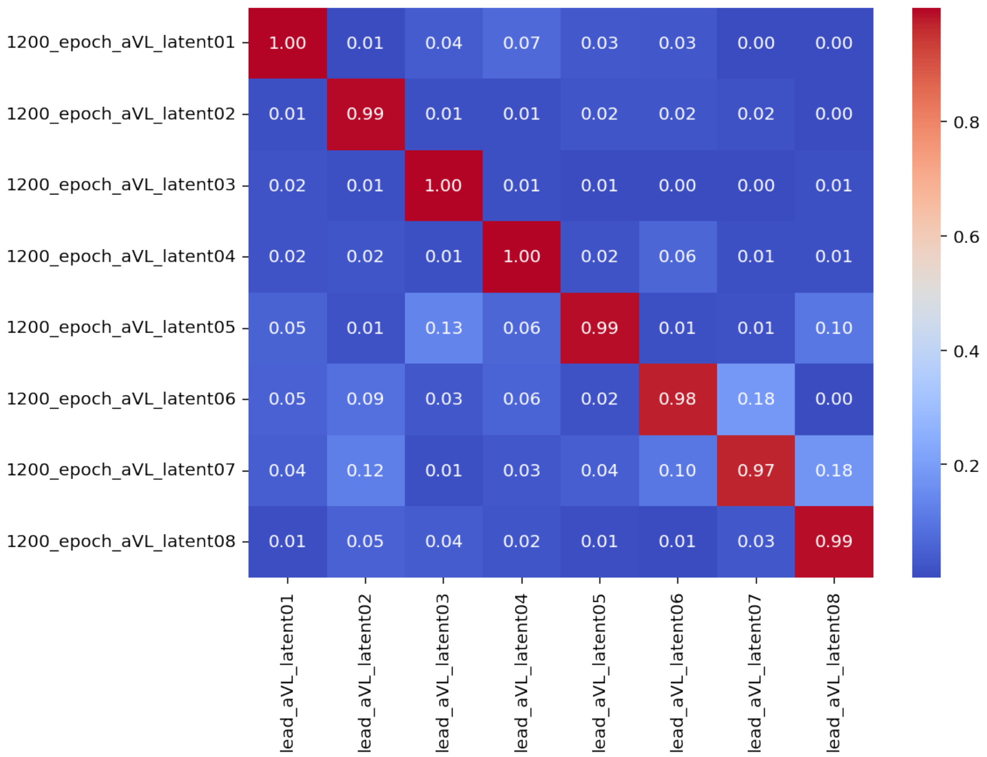

Figure S16: **Correlation coefficient matrix of the best unimodal ECG lead aVL embeddings within 300 training epochs and the best unimodal ECG lead aVL embeddings within 1200 training epochs.** See details in Figure S5)

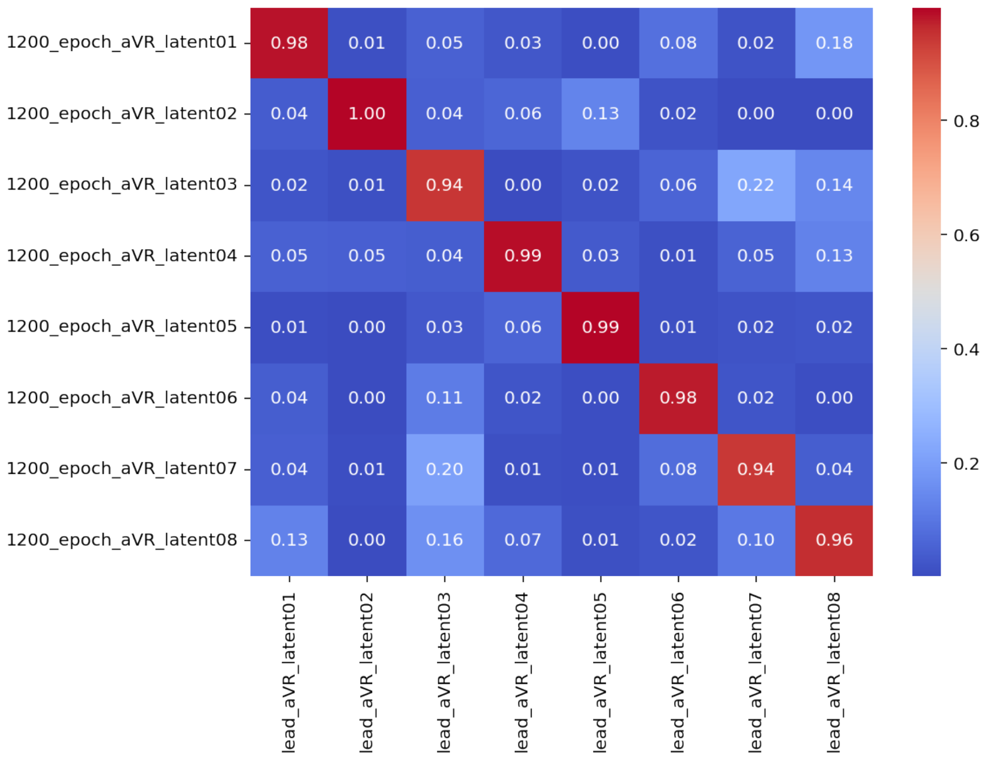

Figure S17: **Correlation coefficient matrix of the best unimodal ECG lead aVR embeddings within 300 training epochs and the best unimodal ECG lead aVR embeddings within 1200 training epochs.** See details in Figure S5)

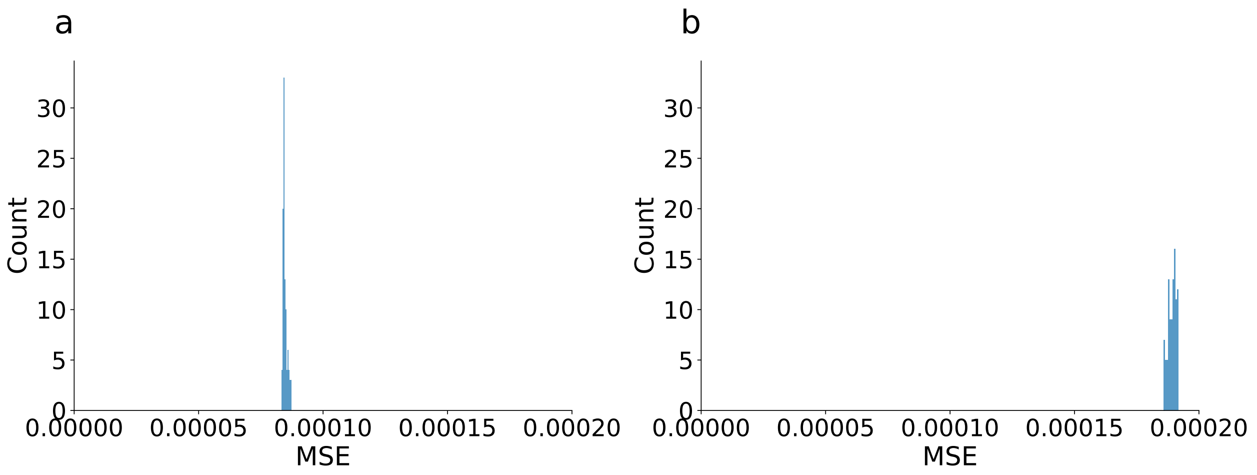

Figure S18: **M-REGLE's performance is robust against random seeds.** a) Histogram of MSEs of 12-lead ECG models (96 latent dimensions) trained on 100 different random seeds. b) Histogram of MSEs of ECGPPG models (12 latent dimensions) trained on 100 different random seeds.

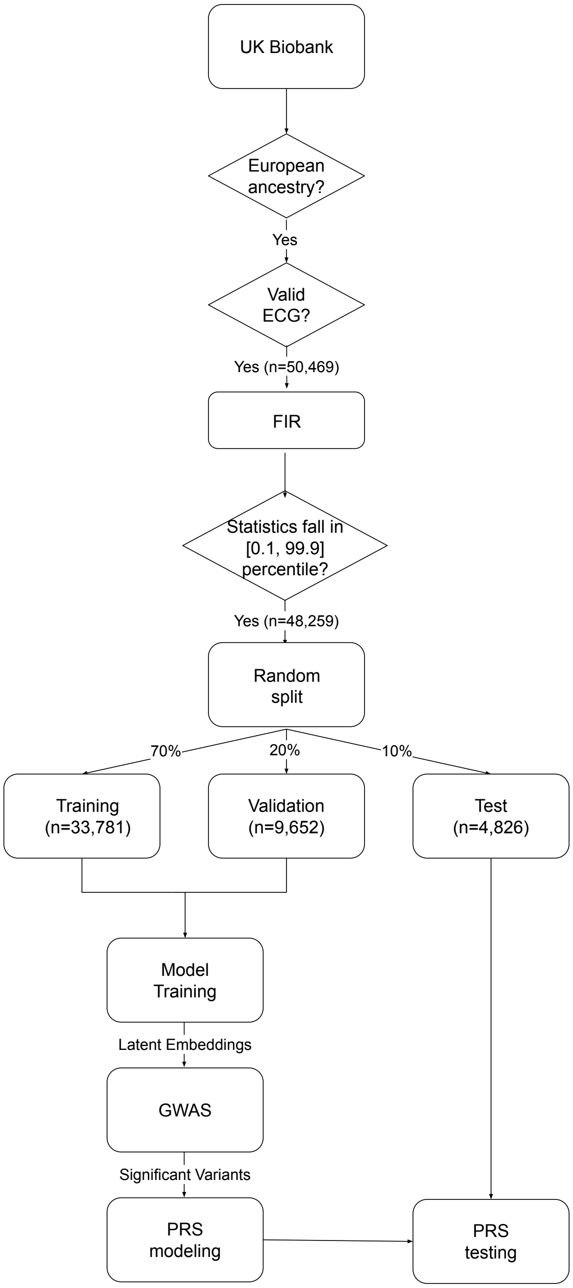

Figure S19: **An overview of UK Biobank 12-lead ECG used in this study** Our initial dataset consists of all European-ancestry in UK Biobank (n=435,766). We considered all individuals with valid 12-lead ECG waveform at instance 2 (n=50,469). After the preprocessing and quality control steps, we split the dataset to training (70%) validation (20%) and test (10%) sets. We use all individuals in training and validation sets for GWAS analysis, and test set for reporting the PRS results.

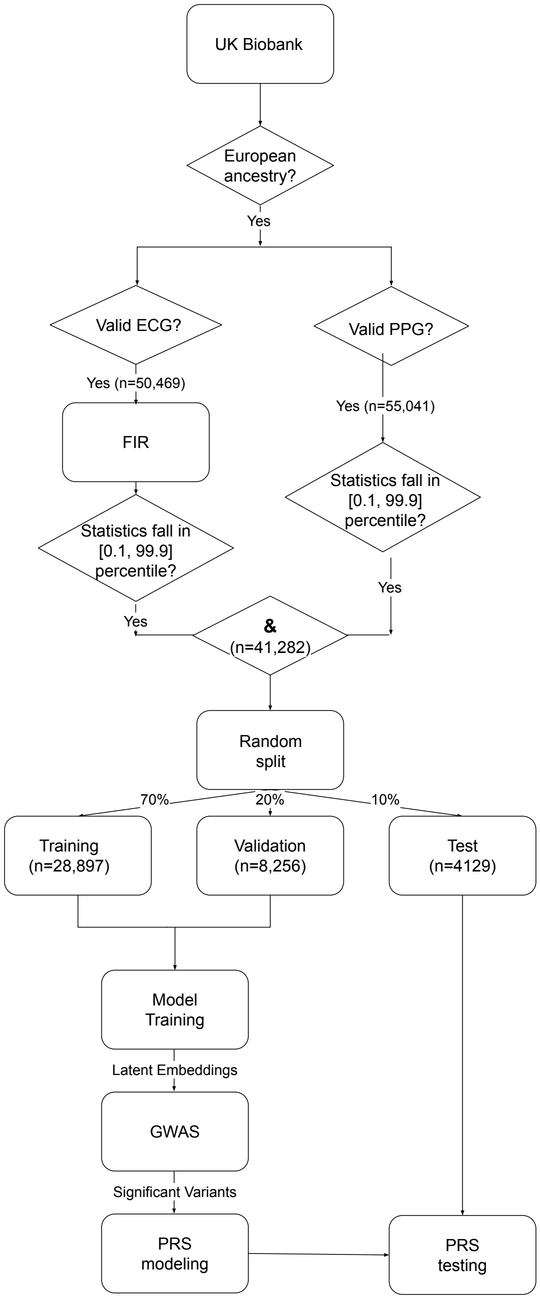

Figure S20: **An overview of UK Biobank ECG lead I and PPG used in this study** Our initial dataset consists of all European-ancestry in UK Biobank (n=435,766). We considered all individuals with valid ECG lead I (n=50,469) and PPG (n=55,041) waveform at instance 2. After the preprocessing and quality control steps, we get all the individuals with both ECG and PPG qualified. We split the dataset to training (70%) validation (20%) and test (10%) sets. We use all individuals in training and validation sets for GWAS analysis, and test set for reporting the PRS results.

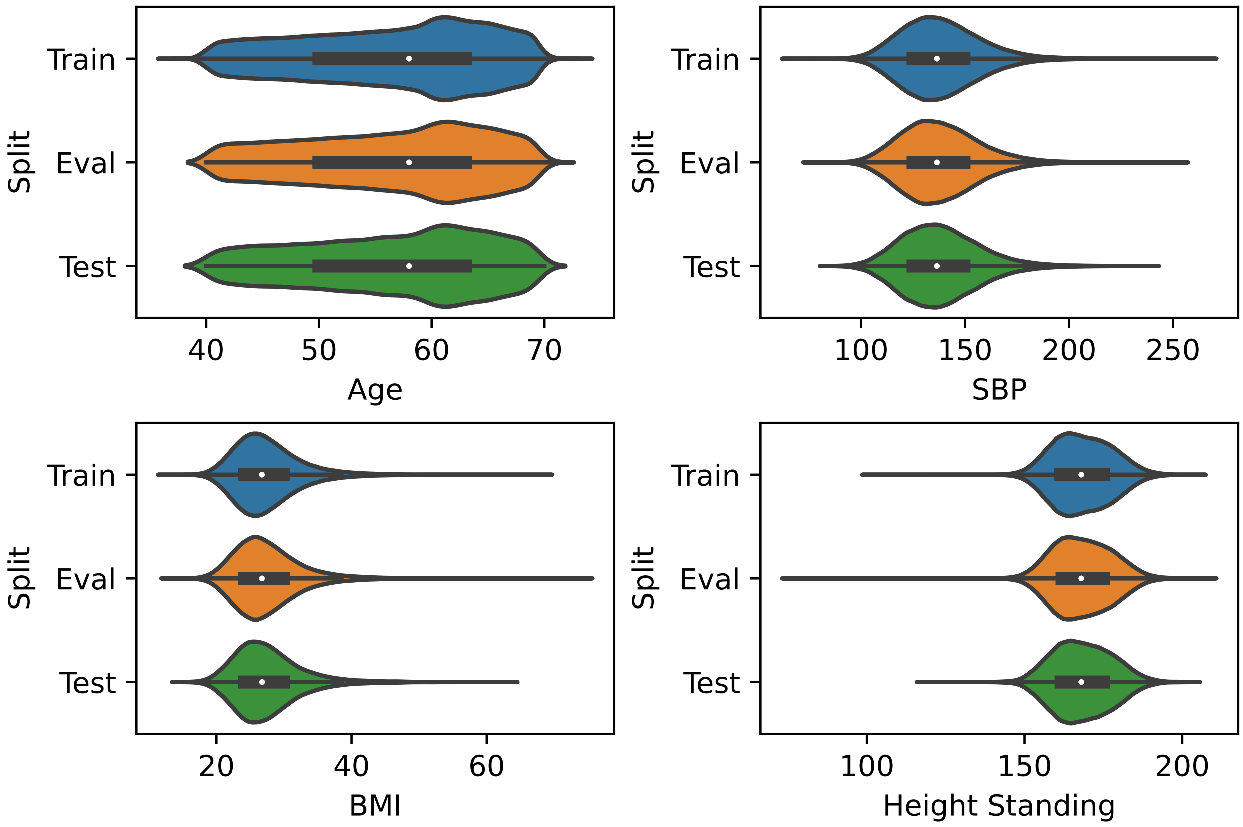

Figure S21: **UK Biobank phenotype distribution for different data splits** We plot the age, SBP (systolic blood pressure), BMI (body mass index), and Height standing for train, eval, and test datasets in UKB. We observed that in most cases all datasets tend to have similar phenotypic distribution.

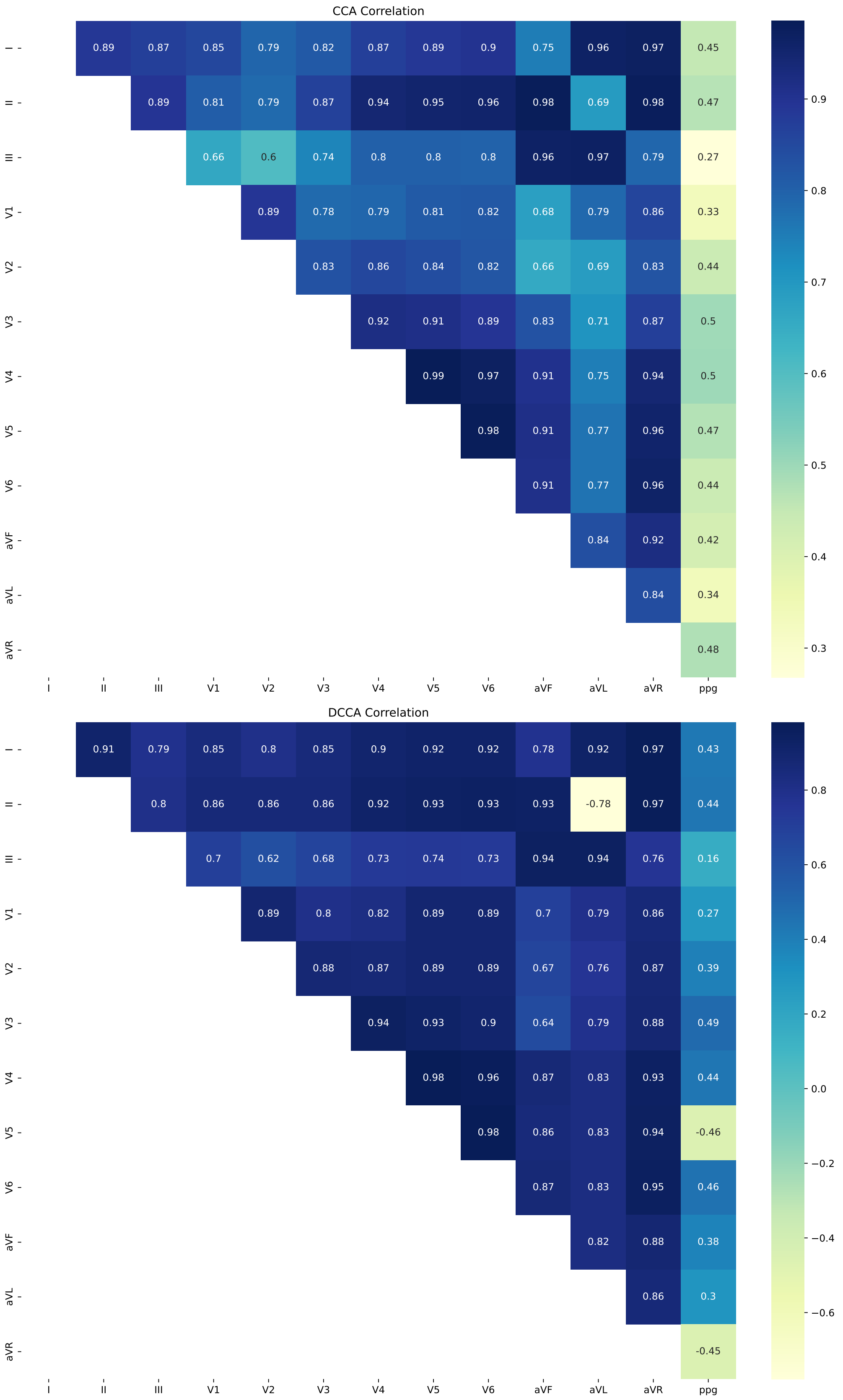

Figure S22: **CCA and DCCA between 12 ECG leads and PPG in UK Biobank.** We plotted the mean correlation obtained from CCA and DCCA. The standard error of correlation and numerical values see Table S8.

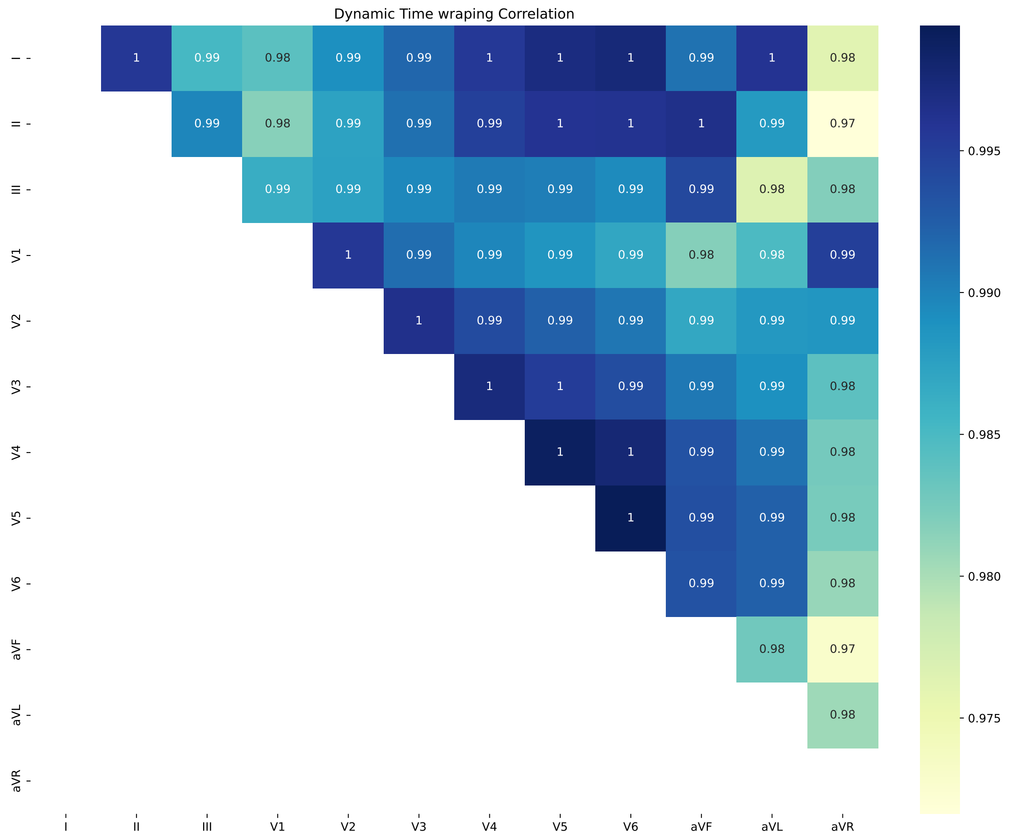

Figure S23: **Dynamic time warping between 12 ECG leads in UK Biobank.** We plotted the mean correlation obtained from dynamic time warping. We use the following formula: $r = 1 - D/2n$ where D is the computed distance, and n is the number of time points which we set 600 in the case of ECG 12 leads.

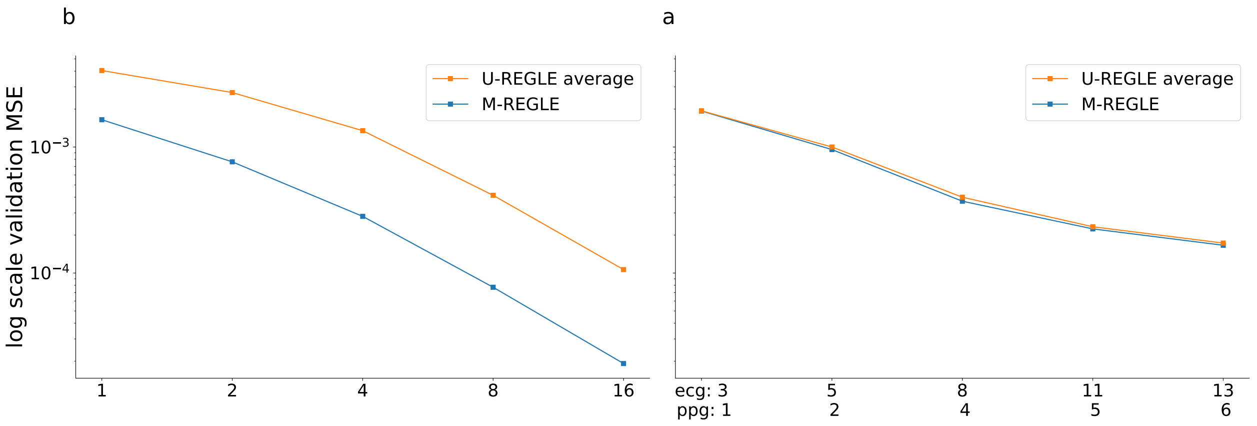

Figure S24: **Reconstruction errors of unimodal modal PCA and multimodal PCA across different PC numbers.** a) Plot of log scale validation reconstruction loss of unimodal modal PCA and multimodal PCA across different PC numbers on Lead I ECG + PPG data setting.b) Plot of log scale validation reconstruction loss of unimodal modal PCA and multimodal PCA across different PC numbers on 12-lead ECG data setting

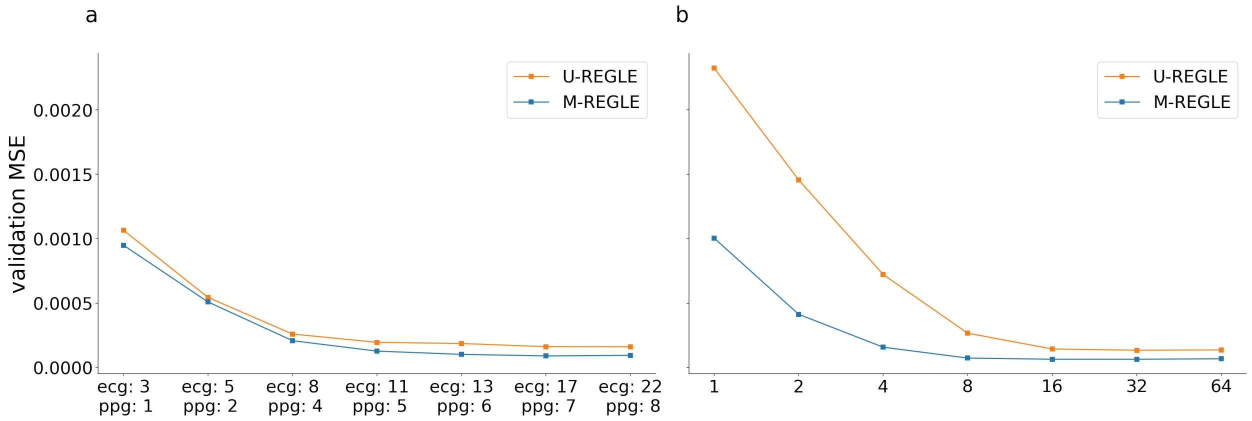

Figure S25: **Validation reconstruction losses of M-REGLE and U-REGLE across numbers of latent dimensions on Lead I ECG + PPG and 12-lead ECG datasets.** The differences are all significant. See Tables S9 and S13 for the values of reconstruction losses and standard errors.

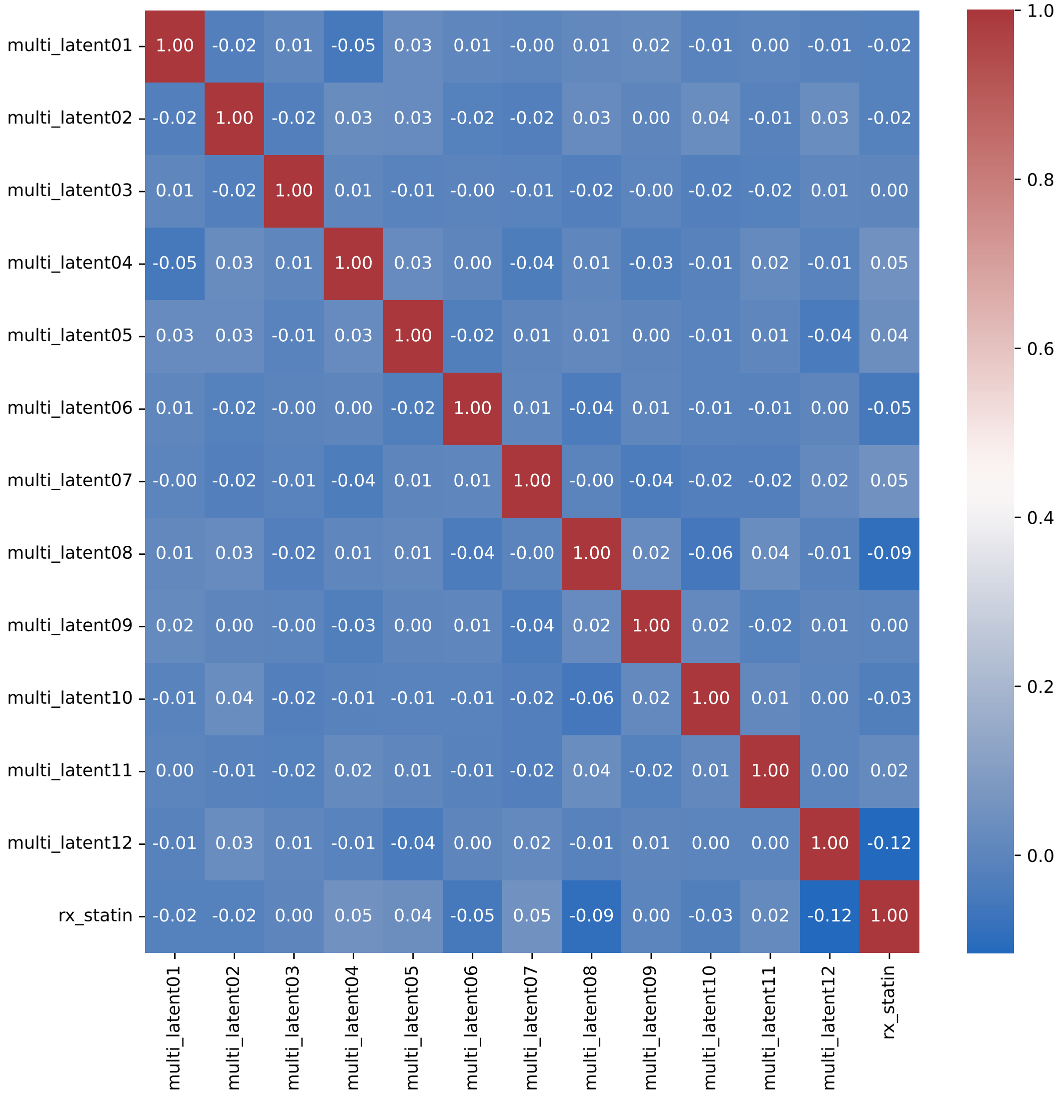

Figure S26: **The pairwise correlation between lead I ECG + PPG M-REGLE embeddings and Statin taken status in UK Biobank.** We consider any individual taken any of ’fluvastatin’, ’lovastatin’, ’rosuvastatin’, ’pitavastatin’, ’simvastatin’, ’atorvastatin’, ’pravastatin’ as taken of Statin.

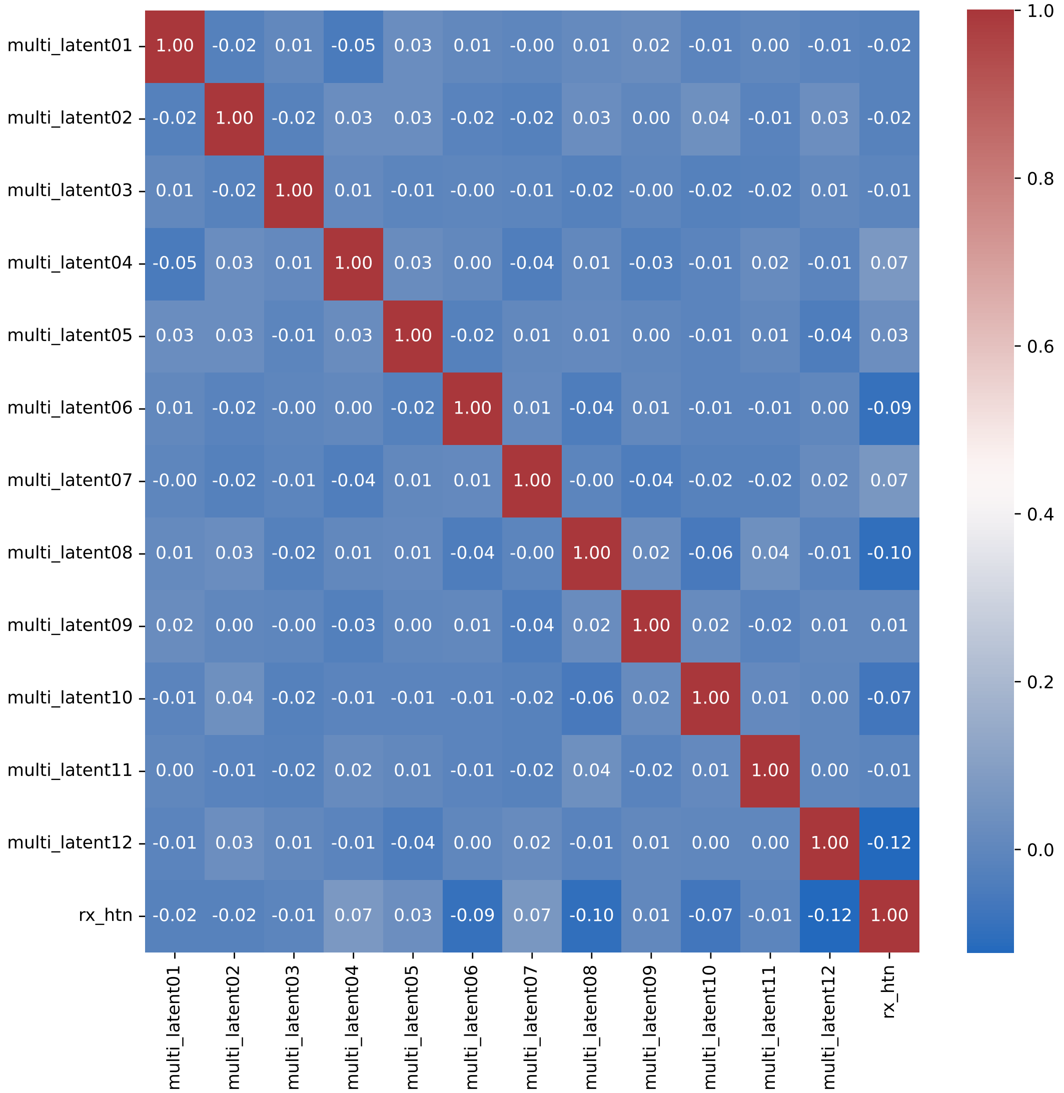

Figure S27: **The pairwise correlation between lead I ECG + PPG M-REGLE embeddings and hypertension (HTN) taken status in UK Biobank.**

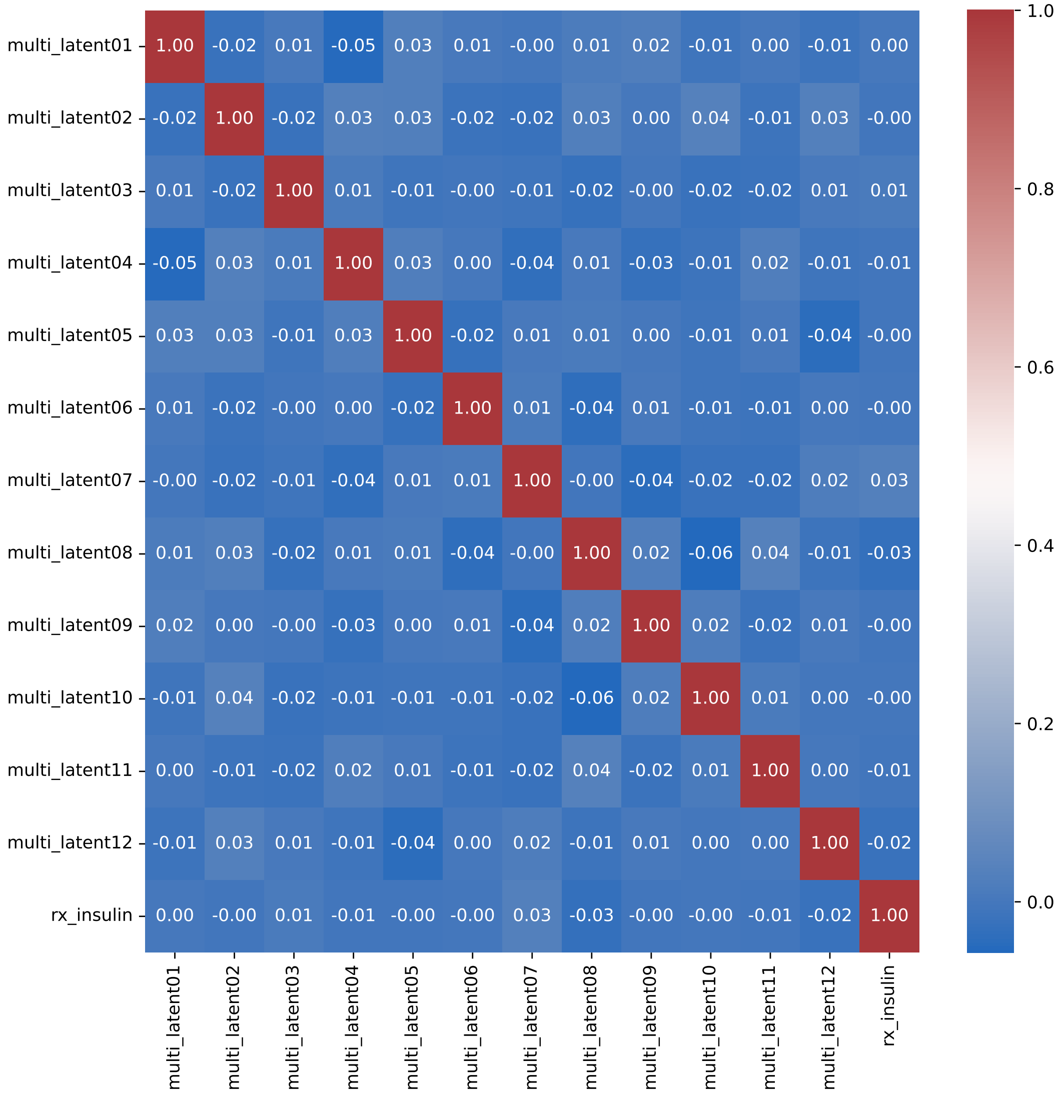

Figure S28: **The pairwise correlation between lead I ECG + PPG M-REGLE embeddings and Insulin taken status in UK Biobank.**

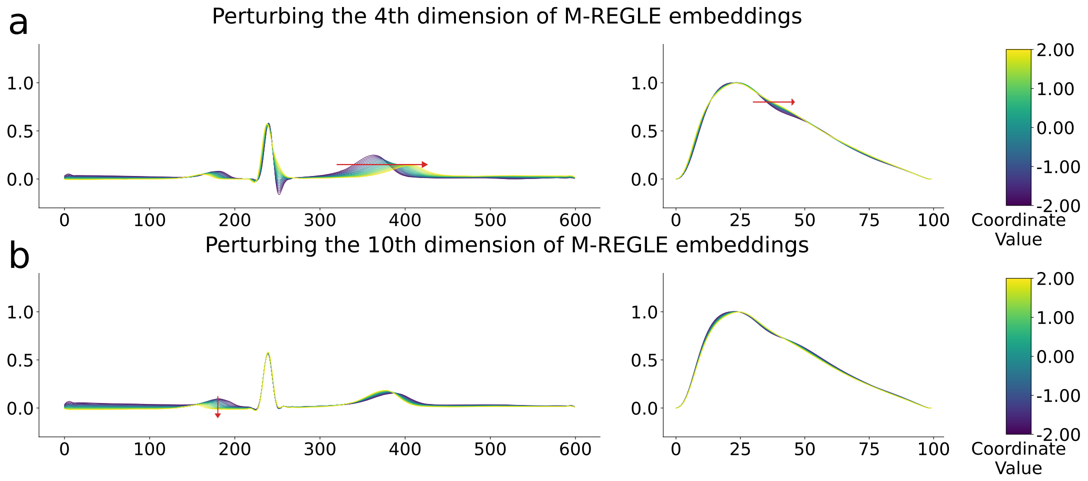

Figure S29: **The effect of perturbing one coordinate (4th or 10th) of M-REGLE embeddings from [-2, 2], while the values of the rest coordinates are 0s.** a) The impact of the 4th dimension. As the embedding transitions from -2 to 2, the decoded ECG (left) exhibits a prolonged QT interval, while the PPG (right) loses its notch. b) The impact of the 10th dimension. A shift from -2 to 2 along this dimension results in a progressive decrease in ECG amplitude.

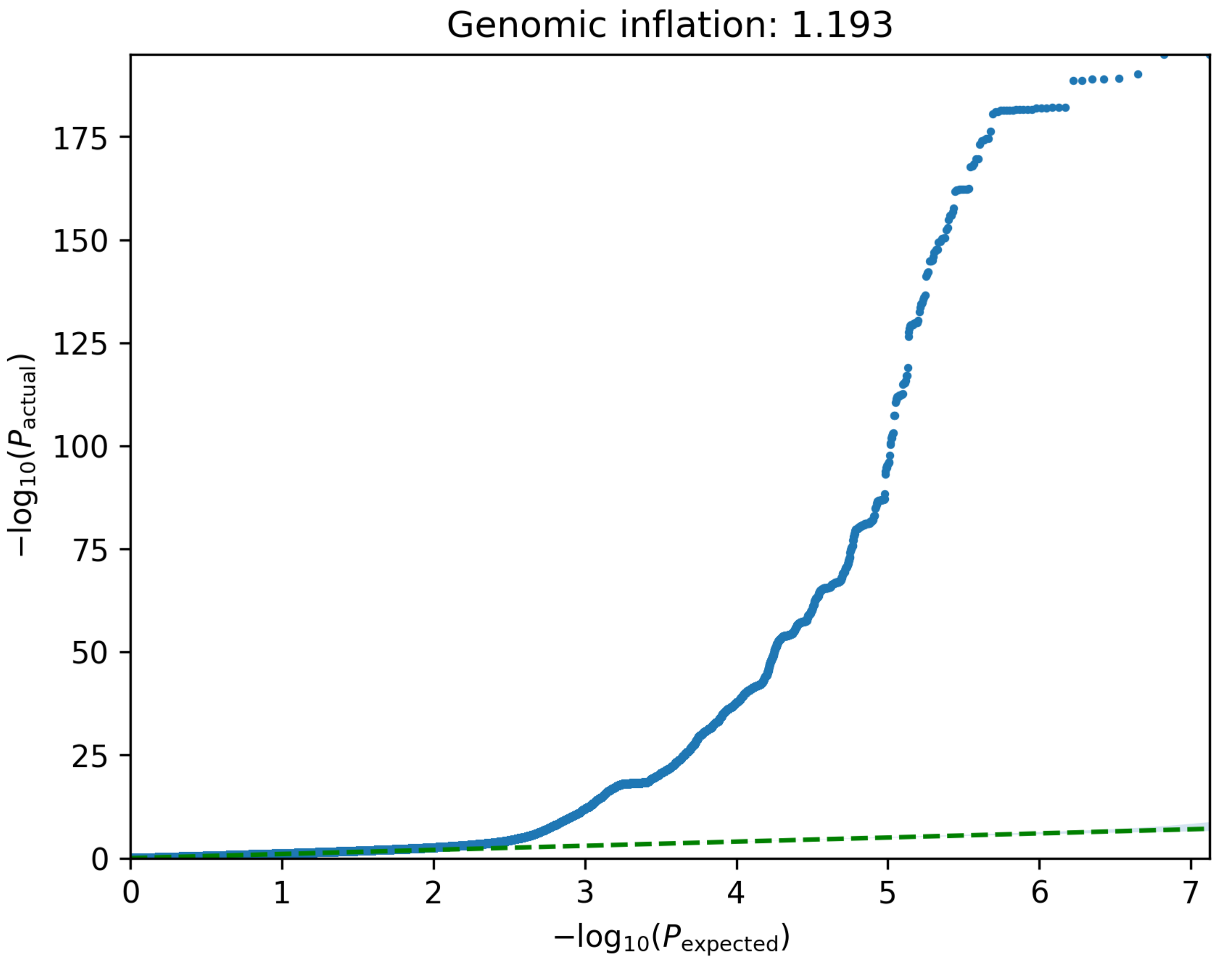

Figure S30: **QQ-plot M-REGLE combined on all 96 embeddings obtained from 12 lead ECG.**

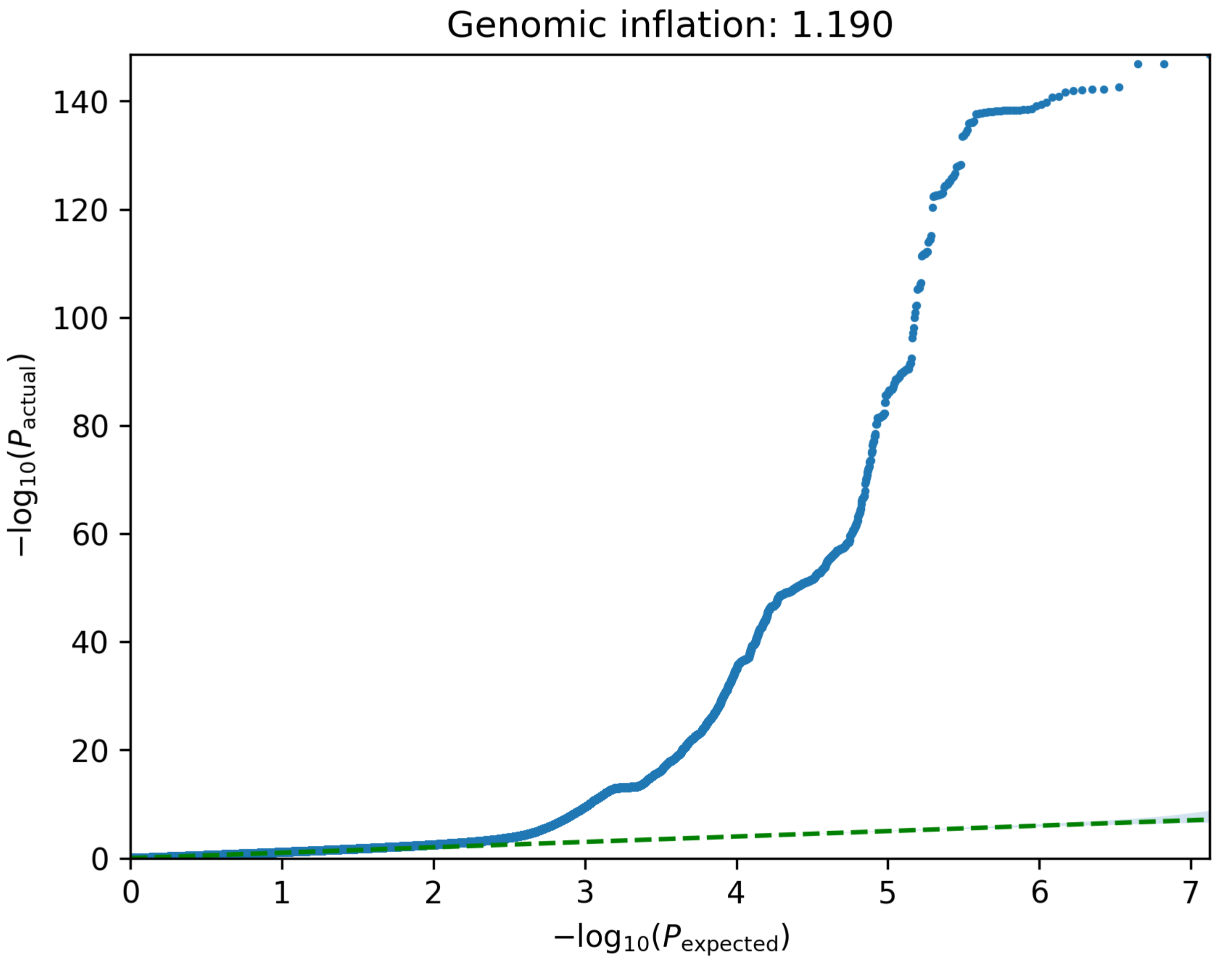

Figure S31: **QQ-plot U-REGLE combined on all 96 embeddings obtained from 12 lead ECG.**

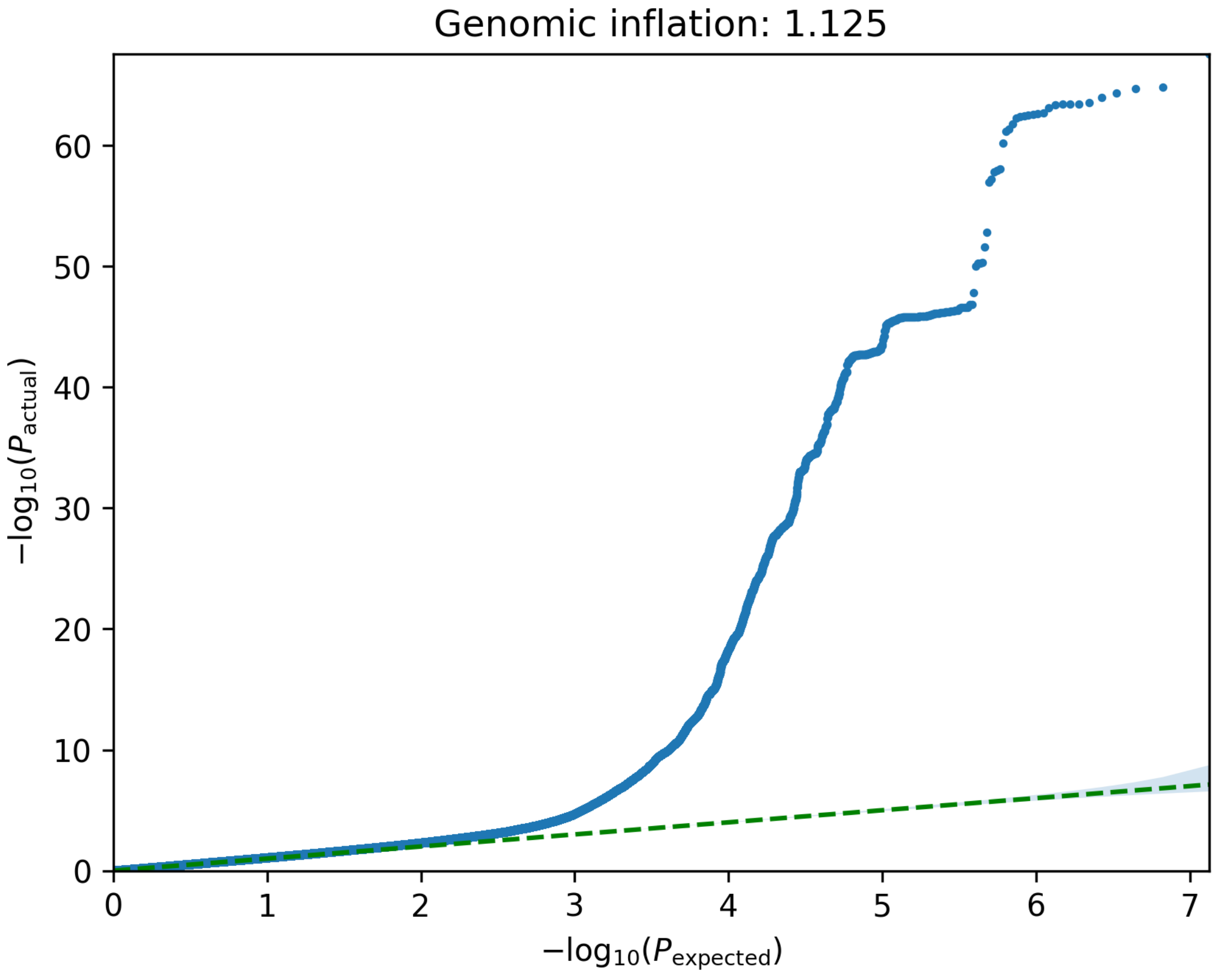

Figure S32: **QQ-plot M-REGLE combined on all 12 embedding obtained from lead I ECG and PPG.**

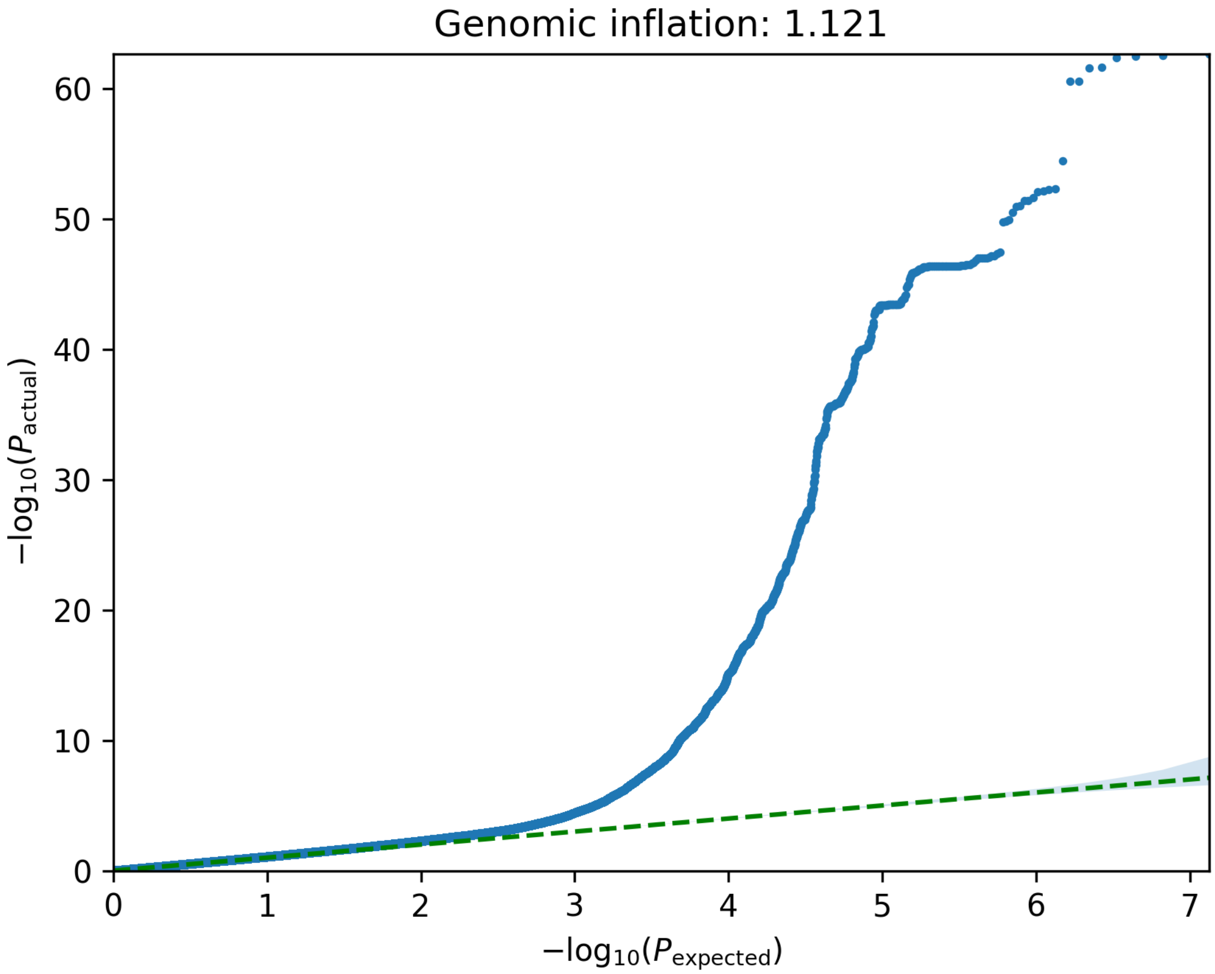

Figure S33: **QQ-plot U-REGLE combined on all 12 embedding obtained from lead I ECG and PPG.**

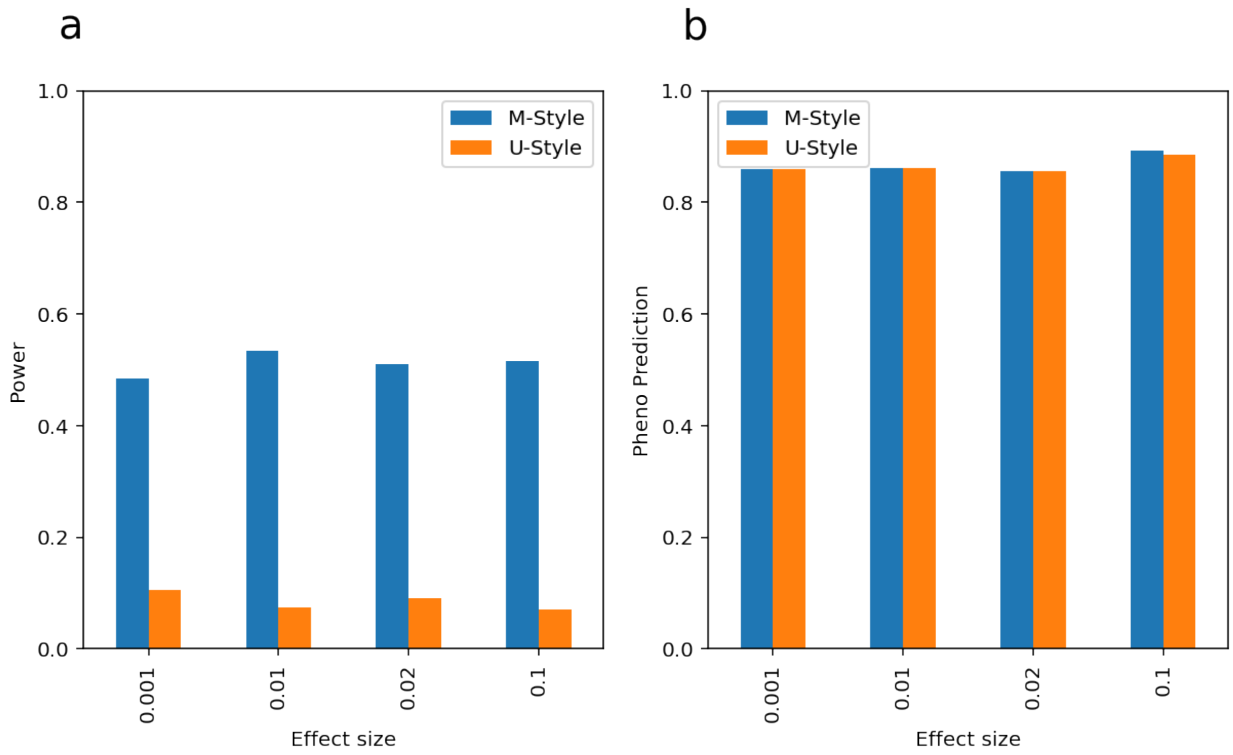

Figure S34: **Models with similar phenotype prediction can have different genetic analysis** We simulated two data modalities where in both modalities both genetic and non-genetic factor contribute to the data. However, the effect of non-genetic on data is larger than genetic effect. Panel (a) compute the power to detect the genetic factor and Panel (b) indicates the phenotype prediction from the learned embeddings. Effect size is the strength of genetic factor on the simulated phenotype of interest.

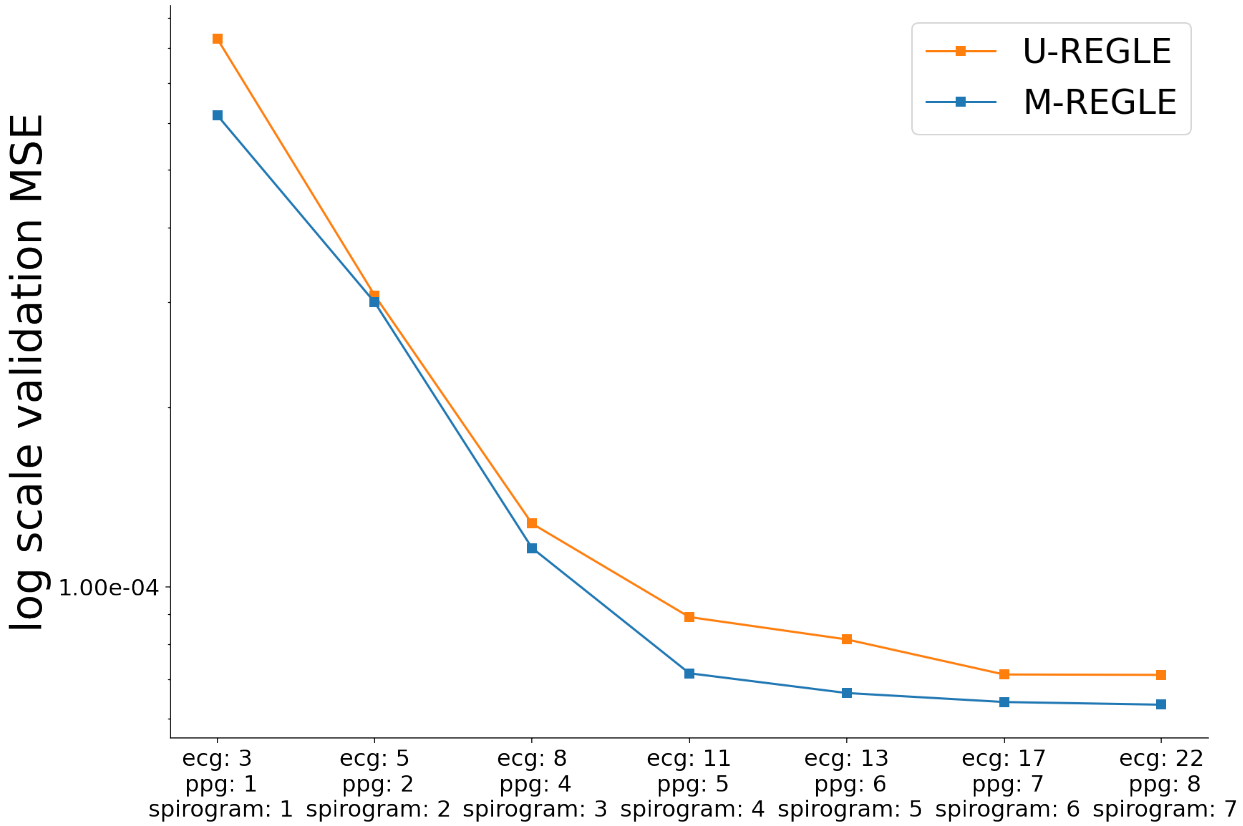

Figure S35: **Validation reconstruction losses in log-scale of M-REGLE and U-REGLE across numbers of latent dimensions on Lead I ECG, PPG and Spirogram dataset.** The differences are all significant. See Table S50 for the values of reconstruction losses and standard errors.

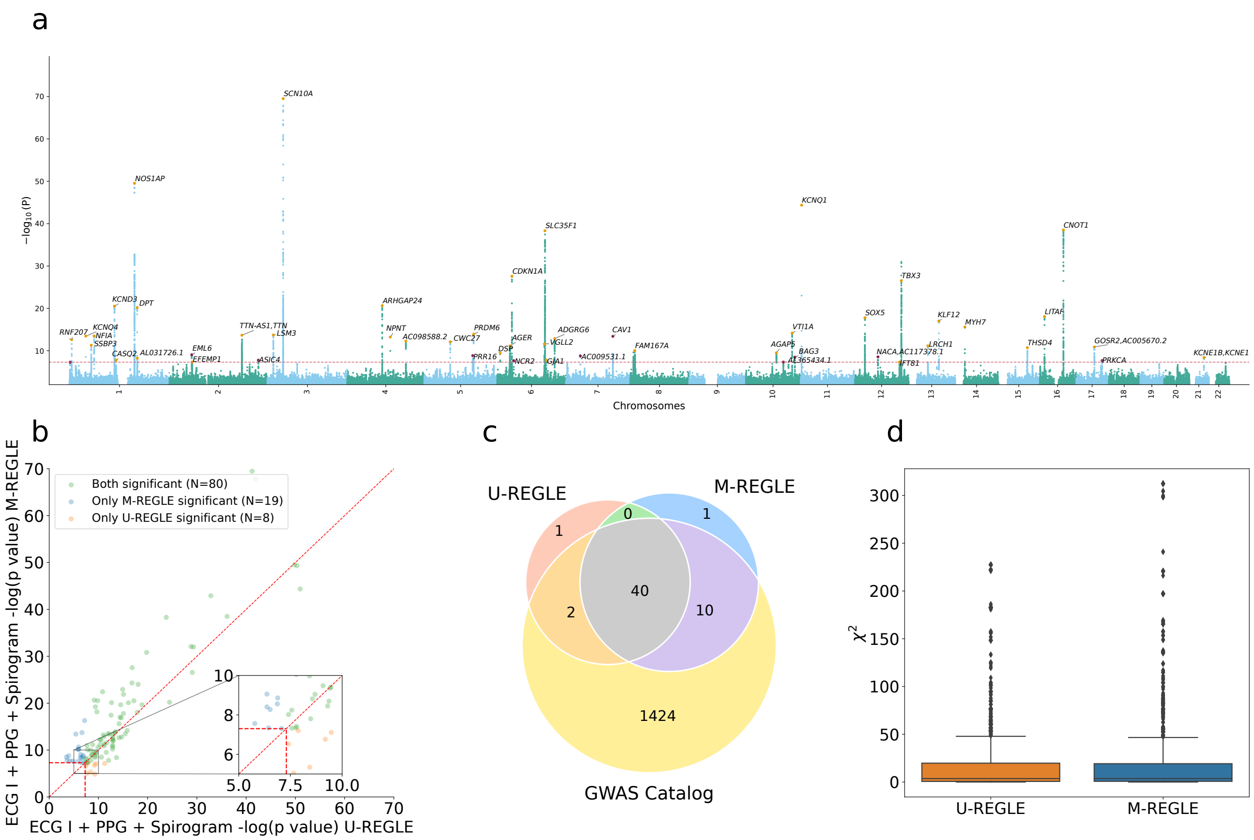

Figure S36: **M-REGLE on ECG lead I, PPG, and Spirograms increases genomic discovery**. a) Manhattan plot depicting M-REGLE GWAS p-values. Black gene names indicate the closest gene for each locus with $-\log_{10} p > 20$. Purple dots denote the GWS loci detected uniquely by M-REGLE. Orange dots indicate loci also identified in U-REGLE. b) Comparison of M-REGLE GWS variants-in-hits with U-REGLE. The X-axis is the $-\log$ p-value of Baseline. The Y-axis is the $-\log$ p-value of the M-REGLE. All p-values (a) and (b) are computed by summing the chi-square statistics for all 12 embeddings to perform a single joint chi-square test. The vertical and horizontal red lines indicate the GWS level. The diagonal red line indicates $y = x$. The orange dots indicate variants-in-hits that are significant for U-REGLE but not significant for our M-REGLE and green dots indicate variants-in-hits that are significant for our M-REGLE but not significant for Baseline. c) A 3 way Venn diagram of the GWAS catalog loci, loci discovered by M-REGLE and loci discovered by U-REGLE. d) Comparison of the chi-square statistics for all known significant variants in GWAS catalog for both U-REGLE and M-REGLE. The difference is statistically significant.

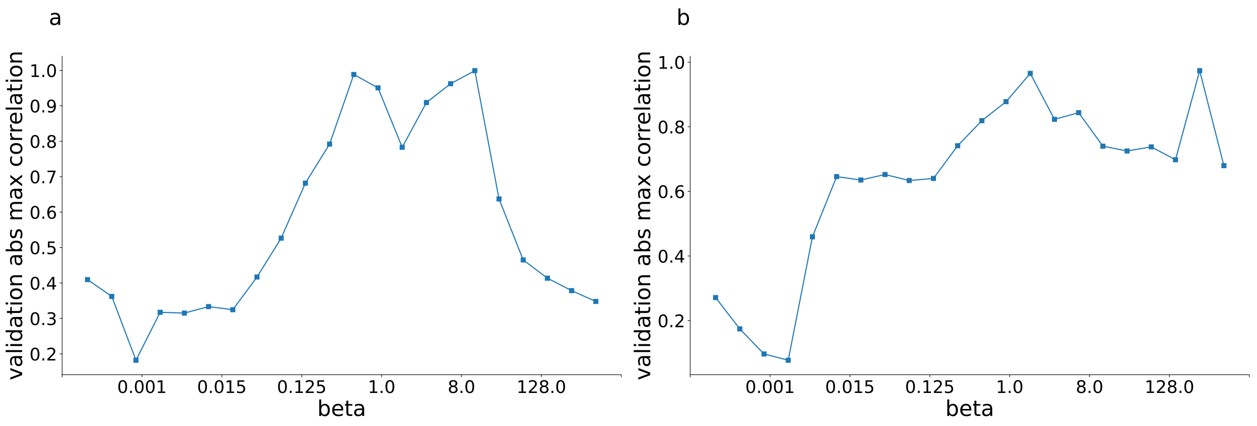

Figure S37: **Plot of maximum absolute non-diagonal correlation coefficients of M-REGLE lower embeddings when training with different beta values.** a) Maximum absolute non-diagonal correlation coefficients VS beta plot of 12-lead ECG, b) Maximum absolute non-diagonal correlation coefficients VS beta plot of ECG+PPG.

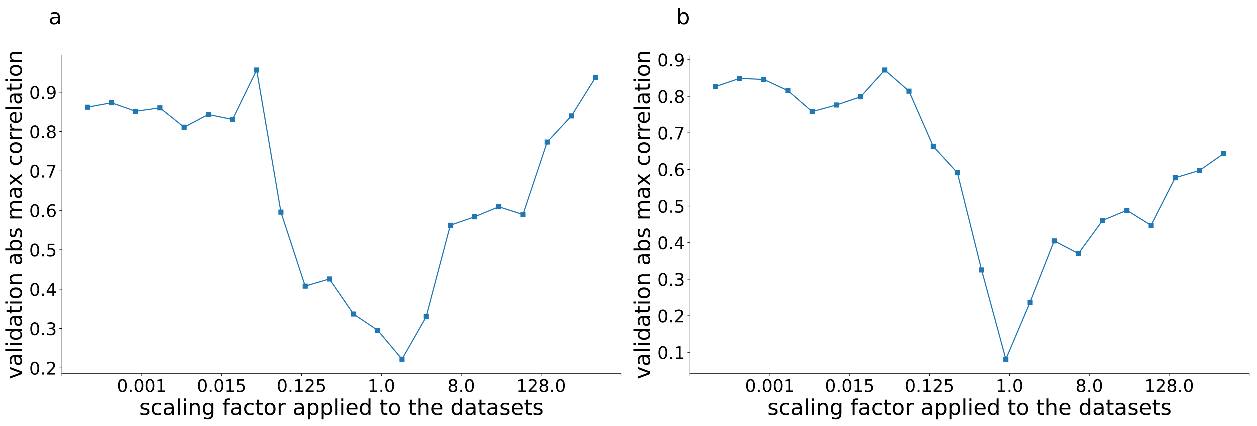

Figure S38: **Plot of maximum absolute non-diagonal correlation coefficients of M-REGLE lower embeddings when different scaling factors applied to the datasets.** a) Maximum absolute non-diagonal correlation coefficients VS scaling factor plot of 12-lead ECG, b) Maximum absolute non-diagonal correlation coefficients VS scaling factor plot of ECG+PPG.

Supplemental Tables

ρ	N	Naive	PCA	MANOVA
	5000	1.05 (0.14)	1.06 (0.14)	1.06 (0.14)
	10000	1.02 (0.20)	1.02 (0.20)	1.02 (0.20)
0	50000	1.07 (0.15)	1.07 (0.15)	1.07 (0.15)
	100000	1.07 (0.15)	1.07 (0.14)	1.07 (0.14)
	200000	1.08 (0.14)	1.08 (0.14)	1.08 (0.14)
	5000	1.09 (0.17)	1.12 (0.16)	1.12 (0.16)
	10000	1.15 (0.22)	1.02 (0.15)	1.02 (0.15)
0.2	50000	1.09 (0.17)	0.98 (0.13)	0.98 (0.13)
	100000	1.11 (0.16)	0.97 (0.12)	0.97 (0.12)
	200000	0.83 (0.15)	0.81 (0.11)	0.81 (0.11)
	5000	1.24 (0.18)	0.90 (0.12)	0.90 (0.12)
	10000	1.51 (0.39)	0.94 (0.15)	0.94 (0.15)
0.3	50000	1.11 (0.19)	0.96 (0.11)	0.96 (0.11)
	100000	1.43 (0.25)	0.84 (0.10)	0.84 (0.10)
	200000	1.26 (0.32)	0.90 (0.12)	0.90 (0.12)

Table S1: **Type I error comparison.** We compared three models: (1) a naive model where we tested each factor independently and then summed the chi-square statistics; (2) a PCA model where we performed PCA on the factors and then summed the chi-square statistics; and (3) multivariate analysis of variance (MANOVA).

ρ	N	Naive	PCA	MANOVA
0	5000	1.15 (0.13)	1.14 (0.13)	1.14 (0.13)
	10000	1.43 (0.16)	1.43 (0.16)	1.43 (0.16)
	50000	2.96 (0.28)	2.95 (0.28)	2.95 (0.28)
	100000	4.76 (0.43)	4.76 (0.43)	4.76 (0.43)
	200000	12.73 (0.70)	12.73 (0.70)	12.73 (0.70)
0.2	5000	1.20 (0.19)	1.14 (0.16)	1.14 (0.16)
	10000	1.92 (0.23)	1.73 (0.18)	1.73 (0.18)
	50000	2.41 (0.25)	2.74 (0.26)	2.74 (0.26)
	100000	5.17 (0.49)	6.16 (0.52)	6.16 (0.52)
	200000	10.54 (0.62)	13.02 (0.70)	13.02 (0.70)
0.3	5000	1.32 (0.231)	1.24 (0.15)	1.24 (0.151)
	10000	1.57 (0.231)	1.59 (0.17)	1.59 (0.171)
	50000	3.21 (0.401)	4.29 (0.42)	4.29 (0.421)
	100000	5.10 (0.441)	6.66 (0.45)	6.66 (0.451)
	200000	11.58 (0.731)	16.34 (0.91)	16.35 (0.911)

Table S2: **Power comparison.** We compared three models: (1) a naive model where we tested each factor independently and then summed the chi-square statistics; (2) a PCA model where we performed PCA on the factors and then summed the chi-square statistics; and (3) multivariate analysis of variance (MANOVA).

See the attached Excel table.

Table S3: **Overview of hyperparameter sweep ranges for M-REGLE and U-REGLE VAE models.**

See the attached Excel table.

Table S4: **Overview of final hyperparameters used in M-REGLE and U-REGLE VAE models.**

Latent dimensions	Combine modalities into 1 channel	Combine modalities into 2 channels
4	9.49e-04 ± 1.98e-06	1.174-03 ± 1.2e-05
7	5.09e-04 ± 8.86e-07	6.07e-04 ± 7e-06
12	2.08e-04 ± 3.82e-07	2.56e-04 ± 3e-06
16	1.27e-04 ± 2.60e-07	1.55e-04 ± 2e-06
19	1.02e-04 ± 1.90e-07	1.14e-04 ± 2e-07

Table S5: **Comparison of the MSE of different VAE model architectures across different latent dimension numbers on lead I ECG + PPG dataset.** For learning M-REGLE representations on Lead I ECG + PPG modalities, besides the model architecture we used in the paper, where we combined the ECG waveform and PPG waveform by concatenating them into one channel, we also tried the model architecture of using ECG and PPG as 2 channels. We first upsampled PPGs, to match the length of ECGs, and they can be fed into the input of 2 channels. We trained the VAE model to reconstruct ECG and upsampled PPG. As the table shows, this 2-channel model does not perform as well as the model we used in the paper. This result is expected. ECG and PPG of the same individual are not collected synchronously, but putting them into 2 input channels may indicate same time stamps at each pair of aligned data points, which is not true. Therefore, this can hurt the model's performance.

12 lead ECG	lead I ECG + PPG	lead I ECG + PPG + Spirograms
Electrocardio	Electrocardio	Electrocardio
ECG	ECG	ECG
EKG	ECK	ECK
PR interval	PR interval	PR interval
Ventricular rate	Ventricular rate	Ventricular rate
PP interval	PP interval	PP interval
PQ interval	PQ interval	PQ interval
QRS duration	QRS duration	QRS duration
QT interval	QT interval	QT interval
RR interval	RR interval	RR interval
P axis	P axis	P axis
R axis	R axis	R axis
T axis	T axis	T axis
	Arterial stiffness	Arterial stiffness
	Pulse wave	Pulse wave
	Pulse waveform	Pulse waveform
	Pulse-wave	Pulse-wave
	Notch position	Notch position
		COPD
		Asthma
		FEV1
		FVC
		Lung function
		PEF
		FEV/FVC ratio

Table S6: **Regular expression key words used for search in GWAS catalog.**

	Train	Eval	Test
Myocardial Infarction	0.0593	0.0599	0.0585
Afib	0.0722	0.0729	0.0709
T2D	0.0836	0.0825	0.0828
Cardiovascular Disease	0.4149	0.4116	0.4150
Hypertension	0.3941	0.3909	0.3942
Ever smoked	0.6031	0.6013	0.6008

Table S7: **UK Biobank binary phenotypes prevalence for different data splits.**

See the attached Excel table.

Table S8: **CCA and DCCA between 12 ECG leads and PPG in UK Biobank.**

Latent Dim. per channel	Multimodal	Unimodal
1	1.00e-03 ± 6.77e-07	2.32e-03 ± 1.43e-06
2	4.13e-04 ± 2.55e-07	1.46e-03 ± 8.46e-07
4	1.58e-04 ± 1.68e-07	7.22e-04 ± 3.26e-07
8	7.32e-05 ± 8.40e-08	2.67e-04 ± 1.62e-07
16	6.41e-05 ± 7.18e-08	1.46e-04 ± 9.01e-08
32	6.37e-05 ± 1.21e-06	1.36e-04 ± 1.00e-06
64	6.84e-05 ± 1.20e-06	1.40e-04 ± 1.06e-06

Table S9: **Comparison of multimodal VAE MSE and an average of all unimodal modal VAE MSEs across different latent dimension numbers on 12 lead ECG dataset.** Latent Dim stands for latent dimension.

Latent Dim. per channel	Multimodal	Unimodal
1	1.60e-03 ± 1.26e-06	3.88e-03 ± 2.60e-06
2	7.63e-04 ± 6.12e-07	2.62e-03 ± 1.80e-06
4	2.88e-04 ± 2.74e-07	1.33e-03 ± 8.02e-07
8	8.50e-05 ± 1.15e-07	4.38e-04 ± 3.44e-07
16	2.46e-05 ± 4.42e-08	1.17e-04 ± 1.49e-07

Table S10: **Comparison of multimodal PCA MSE and an average of all unimodal modal PCA MSEs across different PC numbers on 12 lead ECG dataset.**

Latent Dim. per channel	Multimodal	Unimodal
1	1.03e-03	2.69e-03
2	4.32e-04	1.71e-03
4	1.64e-04	8.08e-04
8	7.71e-05	2.45e-04
16	4.70e-05	7.97e-05

Table S11: **Comparison of multimodal CAE MSE and an average of all unimodal modal CAE MSEs across different latent dimension numbers on 12 lead ECG dataset.** Latent Dim stands for latent dimension.

# ECG Latent Dim.	ECG variance explained	# PPG Latent Dim.	PPG variance explained
3	0.6807	1	0.6728
5	0.8353	2	0.8370
8	0.9311	4	0.9496
11	0.9616	5	0.9660
13	0.9716	6	0.9763
17	0.9834	7	0.9848
22	0.9902	8	0.9912

Table S12: **Latent dimension number and variance explained through PCA for Lead I ECG and PPG.**

Variance explained	Multimodal	Unimodal
70%	9.49e-04 ± 1.98e-06	1.07e-03 ± 2.26e-06
83%	5.09e-04 ± 8.86e-07	5.44e-04 ± 9.42e-07
93%-94%	2.08e-04 ± 3.82e-07	2.60e-04 ± 4.46e-07
96%	1.27e-04 ± 2.60e-07	1.95e-04 ± 3.36e-07
97%	1.02e-04 ± 1.90e-07	1.86e-04 ± 3.01e-07
98%	9.05e-05 ± 1.15e-06	1.63e-04 ± 1.51e-06
99%	9.39e-05 ± 1.23e-06	1.64e-04 ± 1.50e-06

Table S13: **Comparison of multimodal VAE MSE and an average of all unimodal modal VAE MSEs across different latent dimension numbers on lead I ECG + PPG dataset.**

Variance explained	Multimodal	Unimodal
70%	1.93e-03 ± 4.64e-06	1.94e-03 ± 4.65e-06
83%	9.54e-04 ± 2.78e-06	1.00e-03 ± 2.37e-06
93%-94%	3.71e-04 ± 1.13e-06	3.99e-04 ± 1.29e-06
96%	2.24e-04 ± 8.68e-07	2.33e-04 ± 8.66e-07
97%	1.66e-04 ± 7.55e-07	1.73e-04 ± 7.84e-07

Table S14: **Comparison of multimodal PCA MSE and an average of all unimodal modal PCA MSEs across different latent dimension numbers on lead I ECG + PPG dataset.**

Variance explained	Multimodal	Unimodal
70%	9.72e-04	9.43e-04
83%	5.48e-04	4.99e-04
93%-94%	1.90e-04	1.93e-04
96%	1.16e-04	1.14e-04
97%	8.61e-05	8.47e-05

Table S15: **Comparison of multimodal CAE MSE and an average of all unimodal modal CAE MSEs across different latent dimension numbers on lead I ECG + PPG dataset.**

See the attached Excel table.

Table S16: **M-REGLE phenotypic correlation with UKB phenotypes.**

Pheno	PRS Sample		ECG+PPG		12 lead ECG	
	Control	Cases	Control	Cases	Control	Cases
Afib	430076	33513	33536	1415	38993	1651
All cause mortality	444096	42840	35505	592	41536	669
Angina	452379	34772	34381	1716	40246	1959
Cardiovascular disease	285273	201790	23511	12585	27811	14393
Coronary artery disease	439209	47942	33974	2123	39749	2456
Diabetes type 2	424934	38655	33247	1704	38705	1939
Hypertension	281821	182847	23590	11389	27712	12985
Myocardial_infarction	458149	28914	34774	1322	40672	1532
Stroke	424003	15903	30977	679	36118	781

Table S17: **Sample size of UK Biobank phenotypes.**

See the attached Excel table.

Table S18: **Phenotype prediction metrics by 12-lead ECG M-REGLE & U-REGLE embeddings.**

See the attached Excel table.

Table S19: **Paired test of phenotype prediction metrics by 12-lead ECG M-REGLE & U-REGLE embeddings.**

See the attached Excel table.

Table S20: **Phenotype prediction metrics by ECG lead I + PPG M-REGLE & U-REGLE embeddings.**

See the attached Excel table.

Table S21: **Paired test of phenotype prediction metrics by ECG lead I + PPG M-REGLE & U-REGLE embeddings.**

Feature	Num. Features	UKB Field Codes
Age	1	21003
Sex	1	31
BMI	1	21001
Smoking	1	20160
HDL	1	30760
LDL	1	30780
Obesity	1	130792 and ICD-10 E66.*
SBP	1	4080
DBP	1	4079
Metabolic panel (lipid)	251	Category 220
		`https://biobank.ndph.ox.ac.uk/showcase/label.cgi?id=220`
Activity	4	22040, 22033, 904, 22038
Socioeconomic factors	9	738, 26416, 26414, 26412, 26413, 26415, 26411, 26410, 26417
Socioeconomic factors	16	680, 728, 709, 816, 4674, 6142 (3 employment)
Self-reported		6143 (4 transportation), 6138 (4 education levels)

Table S22: **Predictor of known cardiovascular features in UKB.** We consider 3 employment: employed, volunteer, student. We considered 4 education level: college, freshman, high school, and professional qualifications e.g.,: nursing, teaching. We considered 4 mean of transportation: car, bike, walking, and public transport.

Pheno	AUROC	AUPRC	Top 1% prevalence	Top 10% prevalence
Hypertension	0.81 (0.80, 0.82)	0.65 (0.63, 0.67)	0.74 (0.71, 0.77)	0.87 (0.76, 0.96)
Diabetes type 2	0.83 (0.81, 0.85)	0.38 (0.33, 0.43)	0.25 (0.22, 0.28)	0.77 (0.67, 0.86)
Cardiovascular disease	0.80 (0.79, 0.81)	0.66 (0.64, 0.68)	0.77 (0.75, 0.80)	0.87 (0.80, 0.93)
Myocardial infarction	0.74 (0.71, 0.76)	0.13 (0.10, 0.16)	0.15 (0.12, 0.17)	0.22 (0.13, 0.31)
Afib	0.72 (0.70, 0.74)	0.11 (0.09, 0.13)	0.12 (0.10, 0.14)	0.16 (0.10, 0.24)
Coronary artery disease	0.78 (0.76, 0.79)	0.22 (0.18, 0.25)	0.23 (0.21, 0.26)	0.40 (0.28, 0.50)
Stroke	0.73 (0.70, 0.77)	0.06 (0.04, 0.09)	0.06 (0.04, 0.08)	0.10 (0.04, 0.18)
All cause mortality	0.75 (0.72, 0.79)	0.04 (0.03, 0.05)	0.05 (0.03, 0.06)	0.06 (0.02, 0.11)

Table S23: **Prediction of known cardiovascular features (Supplementary Table S22) in UKB.**

Pheno	AUROC	AUPRC	Top 1% prevalence	Top 10% prevalence
Hypertension	0.82 (0.81, 0.83)	0.66 (0.64, 0.68)	0.75 (0.73, 0.78)	0.89 (0.82, 0.96)
Diabetes type 2	0.84 (0.82, 0.86)	0.39 (0.35, 0.44)	0.25 (0.22, 0.28)	0.79 (0.70, 0.88)
Cardiovascular disease	0.81 (0.80, 0.81)	0.67 (0.66, 0.69)	0.78 (0.76, 0.80)	0.91 (0.85, 0.96)
Myocardial infarction	0.75 (0.71, 0.77)	0.15 (0.12, 0.19)	0.15 (0.13, 0.17)	0.30 (0.20, 0.40)
Afib	0.75 (0.73, 0.78)	0.24 (0.19, 0.29)	0.18 (0.15, 0.21)	0.53 (0.43, 0.63)
Coronary artery disease	0.79 (0.77, 0.81)	0.26 (0.22, 0.29)	0.25 (0.22, 0.29)	0.48 (0.37, 0.57)
Stroke	0.74 (0.71, 0.77)	0.07 (0.04, 0.09)	0.06 (0.04, 0.08)	0.09 (0.04, 0.15)
All cause mortality	0.76 (0.73, 0.80)	0.05 (0.03, 0.07)	0.04 (0.03, 0.05)	0.09 (0.04, 0.15)

Table S24: **Prediction of M-REGLE embeddings from 12-lead ECG and known cardiovascular features (Supplementary Table S22) in UKB.**

Pheno	AUROC	AUPRC	Top 1% prevalence	Top 10% prevalence
Hypertension	0.82 (0.81, 0.83)	0.66 (0.64, 0.68)	0.75 (0.73, 0.78)	0.91 (0.84, 0.97)
Diabetes type 2	0.84 (0.82, 0.86)	0.39 (0.35, 0.44)	0.26 (0.22, 0.29)	0.80 (0.70, 0.88)
Cardiovascular disease	0.81 (0.80, 0.81)	0.67 (0.66, 0.69)	0.78 (0.76, 0.81)	0.91 (0.85, 0.96)
Myocardial infarction	0.74 (0.71, 0.78)	0.15 (0.12, 0.19)	0.15 (0.13, 0.17)	0.32 (0.22, 0.38)
Afib	0.75 (0.72, 0.78)	0.24 (0.19, 0.29)	0.19 (0.16, 0.21)	0.56 (0.43, 0.67)
Coronary artery disease	0.79 (0.77, 0.81)	0.25 (0.21, 0.28)	0.24 (0.21, 0.27)	0.50 (0.38, 0.60)
Stroke	0.75 (0.71, 0.78)	0.06 (0.04, 0.09)	0.06 (0.04, 0.09)	0.08 (0.02, 0.17)
All cause mortality	0.76 (0.73, 0.79)	0.05 (0.03, 0.07)	0.05 (0.03, 0.06)	0.08 (0.03, 0.14)

Table S25: **Prediction of U-REGLE embeddings from 12-lead ECG and known cardiovascular features (Supplementary Table S22) in UKB.**

Pheno	AUROC	AUPRC	Top 1% prevalence	Top 10% prevalence
Hypertension	0.82 (0.81, 0.83)	0.67 (0.65, 0.68)	0.76 (0.72, 0.78)	0.90 (0.84, 0.96)
Diabetes type 2	0.84 (0.82, 0.86)	0.41 (0.36, 0.45)	0.26 (0.23, 0.29)	0.82 (0.73, 0.89)
Cardiovascular disease	0.81 (0.79, 0.82)	0.67 (0.65, 0.69)	0.77 (0.74, 0.80)	0.91 (0.84, 0.96)
Myocardial infarction	0.75 (0.72, 0.78)	0.14 (0.11, 0.17)	0.15 (0.12, 0.17)	0.27 (0.18, 0.36)
Afib	0.74 (0.71, 0.76)	0.13 (0.10, 0.16)	0.14 (0.12, 0.16)	0.21 (0.13, 0.32)
Coronary artery disease	0.78 (0.76, 0.81)	0.24 (0.21, 0.27)	0.24 (0.21, 0.26)	0.46 (0.34, 0.58)
Stroke	0.73 (0.69, 0.77)	0.06 (0.04, 0.08)	0.06 (0.04, 0.08)	0.07 (0.03, 0.12)
All cause mortality	0.76 (0.72, 0.79)	0.04 (0.03, 0.06)	0.05 (0.03, 0.06)	0.06 (0.01, 0.11)

Table S26: **Prediction of M-REGLE embeddings from ECG lead I+PPG and known cardiovascular features (Supplementary Table S22) in UKB.**

Pheno	AUROC	AUPRC	Top 1% prevalence	Top 10% prevalence
Hypertension	0.82 (0.81, 0.83)	0.66 (0.64, 0.68)	0.76 (0.72, 0.78)	0.89 (0.82, 0.94)
Diabetes type 2	0.84 (0.82, 0.86)	0.41 (0.36, 0.45)	0.26 (0.23, 0.29)	0.82 (0.73, 0.89)
Cardiovascular disease	0.80 (0.79, 0.82)	0.67 (0.65, 0.69)	0.77 (0.74, 0.80)	0.90 (0.83, 0.96)
Myocardial infarction	0.75 (0.71, 0.77)	0.14 (0.11, 0.17)	0.14 (0.12, 0.17)	0.26 (0.18, 0.35)
Afib	0.74 (0.71, 0.76)	0.13 (0.10, 0.16)	0.14 (0.12, 0.16)	0.22 (0.14, 0.31)
Coronary artery disease	0.78 (0.76, 0.81)	0.24 (0.20, 0.27)	0.24 (0.21, 0.27)	0.44 (0.32, 0.55)
Stroke	0.73 (0.70, 0.78)	0.06 (0.04, 0.08)	0.06 (0.05, 0.08)	0.08 (0.03, 0.15)
All cause mortality	0.76 (0.72, 0.79)	0.04 (0.03, 0.06)	0.05 (0.03, 0.06)	0.06 (0.01, 0.11)

Table S27: **Prediction of U-REGLE embeddings from ECG lead I+PPG and known cardiovascular features (Supplementary Table S22) in UKB.**

Embedding	Rx	Corr R	Odd Ratio
12 ECG	Statin	0.12	3.32
	HTN	0.13	4.51
	Insulin	0.02	20.00
ECG + PPG	Statin	0.12	1.14
	HTN	0.12	1.20
	Insulin	0.03	1.41

Table S28: **The maximum correlation and odd ratio between M-REGLE embeddings and medication status.**

See the attached Excel table.

Table S29: **LDSC heritability and intercept obtained for 96 embeddings obtained from 12-lead ECG.**

See the attached Excel table.

Table S30: **M-REGLE 12 lead ECG GWS loci.** CHR, chromosome; POS, base-pair variant position; EA, effect allele; NEA, non-effect allele; SRC, imputed or genotyped variant; INFO, imputation INFO score (set to 1 for genotyped variants); GWAS p-value. GENE_CONTEXT, genomic context of the variant. Notation for gene context:

- Overlapping gene(s):
  - `[A]`: variant overlaps gene `A`
  - `[A,B]`: variant overlaps genes `A` and `B`
- Downstream genes:
  - `[]A`: variant position is $0 < p \leq 10^3$ bp upstream of closest downstream gene `A`
  - `[]-A`: variant position is $10^3 < p \leq 10^4$ bp upstream of closest downstream gene `A`
  - `[]-A`: variant position is $10^4 < p \leq 10^5$ bp upstream of closest downstream gene `A`
  - `[]--A`: variant position is $10^5 < p \leq 10^6$ bp upstream of closest downstream gene `A`
  - `[]`: closest downstream gene is further than 10^6 bp
- Upstream genes: mirrors downstream gene notation, e.g., `B-[]` means variant position is $10^3 < p \leq 10^4$ bp downstream of closest gene `B`.

Method	Num Discovered Hits	Num Discovered Loci
PCA-unimodal	167 (148, 88.62%)	104 (94, 90.38%)
PCA-multimodal	215 (191, 88.84%)	122 (108, 88.52%)
CAE-unimodal	188 (166, 88.30%)	114 (103, 90.35%)
CAE-multimodal	227 (203, 89.43%)	131 (116, 88.55%)
VAE-unimodal (U-REGLE)	200 (182, 91.00%)	119 (107, 89.92%)
VAE-multimodal (M-REGLE)	262 (231, 88.17%)	142 (122, 85.92%)

Table S31: **Comparison of rediscovered hits and loci by different models and learning methods on 12-lead ECG.** The numbers in each parenthesis are the number and percentage of rediscovered hits/loci in GWAS catalog.

Method	Modality	$E[\chi^2]$	se($E[\chi^2]$)	CI
PCA	unimodal	71.54	1.38	(68.85, 74.3)
PCA	multimodal	90.98	1.68	(87.74, 94.3)
CAE	unimodal	88.22	1.77	(84.84, 91.77)
CAE	multimodal	99.24	1.93	(95.53, 103.05)
VAE	unimodal	92.22	1.82	(88.75, 95.84)
VAE	multimodal	112.48	2.06	(108.67, 116.76)

Table S32: **Comparison of expected Chi-Square statistics of GWAS Catalog variants by different models and learning methods on 12 lead ECG dataset.**

See the attached Excel table.

Table S33: **M-REGLE additional loci associated with cardiovascular features in Open Targets.** We detected 11 loci that were not discovered before for ECG feature GWAS. CHR, chromosome; POS, base-pair variant position; EA, effect allele; NEA, non-effect allele; Open Target, Open Target link to know association; Trait, the name of cardiovascular traits with the highest significance level; P-value; p-value of the highest significance level; Beta, effect size beta; Odd Ratio, odd ratio; and PMID; the previously reported study.

Modality	Merge	Model	Num loci	Num cardio enrichment
ECG	Single	PCA	104	47
ECG	Single	CAE	114	48
ECG	Single	VAE	119	68
ECG	Multi	PCA	122	48
ECG	Multi	CAE	131	63
ECG	Multi	VAE	142	61
ECG+PPG	Single	PCA	44	35
ECG+PPG	Single	CAE	54	57
ECG+PPG	Single	VAE	54	61
ECG+PPG	Multi	PCA	45	32
ECG+PPG	Multi	CAE	59	50
ECG+PPG	Multi	VAE	61	69

Table S34: **Comparison of GREAT enriched cardiovascular term counts by different models and learning methods.**

$Model_1$	$Model_2$	Stronger model	Nominal P-value
ECG Single PCA	ECG Single CAE	-	0.062
ECG Single PCA	ECG Single VAE	Single VAE	3.0e-41
ECG Single CAE	ECG Single VAE	Single VAE	2.3e-43
ECG Single PCA	ECG Multi PCA	Multi PCA	3.9e-13
ECG Single CAE	ECG Multi CAE	Multi CAE	1.1e-29
ECG Single VAE	ECG Multi VAE	Multi VAE	0.022
ECG Multi PCA	ECG Multi CAE	Multi CAE	1.5e-11
ECG Multi PCA	ECG Multi VAE	Multi VAE	2.0e-19
ECG Multi CAE	ECG Multi VAE	Multi VAE	8.2e-6
ECG+PPG Single PCA	ECG+PPG Single CAE	Single CAE	1.3e-13
ECG+PPG Single PCA	ECG+PPG Single VAE	Single VAE	1.4e-5
ECG+PPG Single CAE	ECG+PPG Single VAE	Single CAE	8.9e-5
ECG+PPG Single PCA	ECG+PPG Multi PCA	Single PCA	1.1e-11
ECG+PPG Single CAE	ECG+PPG Multi CAE	Multi CAE	1.4e-5
ECG+PPG Single VAE	ECG+PPG Multi VAE	Multi VAE	1.9e-14
ECG+PPG Multi PCA	ECG+PPG Multi CAE	Multi CAE	4.2e-20
ECG+PPG Multi PCA	ECG+PPG Multi VAE	Multi VAE	3.7e-32
ECG+PPG Multi CAE	ECG+PPG Multi VAE	Multi VAE	5.0e-14

Table S35: **Comparison of significance of significantly enriched cardiovascular term p-values by different models and learning methods.** P-values were computed by two-sided paired T-test on -$\log_{10}$-transformed ontology term nominal p-values.

See the attached Excel table.

Table S36: **LDSC heritability and intercept obtained for 12 embeddings obtained from ECG lead I and PPG.**

See the attached Excel table.

Table S37: **M-REGLE lead I ECG and PPG loci.** CHR, chromosome; POS, base-pair variant position; EA, effect allele; NEA, non-effect allele; SRC, imputed or genotyped variant; INFO, imputation INFO score (set to 1 for genotyped variants); GWAS p-value. GENE_CONTEXT, genomic context of the variant. Notation for gene context:

- Overlapping gene(s):
  - `[A]`: variant overlaps gene `A`
  - `[A,B]`: variant overlaps genes `A` and `B`
- Downstream genes:
  - `[]A`: variant position is $0 < p \leq 10^3$ bp upstream of closest downstream gene `A`
  - `[]-A`: variant position is $10^3 < p \leq 10^4$ bp upstream of closest downstream gene `A`
  - `[]-A`: variant position is $10^4 < p \leq 10^5$ bp upstream of closest downstream gene `A`
  - `[]--A`: variant position is $10^5 < p \leq 10^6$ bp upstream of closest downstream gene `A`
  - `[]`: closest downstream gene is further than 10^6 bp
- Upstream genes: mirrors downstream gene notation, e.g., `B-[]` means variant position is $10^3 < p \leq 10^4$ bp downstream of closest gene `B`.

Method	Num Discovered Hits	Num Discovered Loci
PCA-unimodal	69 (67, 97.10%)	44 (43, 97.73%)
PCA-multimodal	69 (68, 98.55%)	45 (45, 100.00%)
CAE-unimodal	77 (74, 96.10%)	54 (52, 96.30%)
CAE-multimodal	93 (89, 95.70%)	59 (57, 96.61%)
VAE-unimodal (U-REGLE)	89 (85, 95.51%)	54 (52, 96.30%)
VAE-multimodal (M-REGLE)	103 (99, 96.12%)	61 (59, 96.72%)

Table S38: **Comparison of rediscovered hits and loci by different models and learning methods on Lead I ECG + PPG dataset.** The numbers in each parenthesis are the number and percentage of rediscovered hits/loci in GWAS catalog.

Method	Modality	$E[\chi^2]$	se($E[\chi^2]$)	CI
PCA	unimodal	33.12	0.68	(31.82, 34.48)
PCA	multimodal	33.23	0.69	(31.95, 34.66)
CAE	unimodal	37.93	0.73	(36.51, 39.41)
CAE	multimodal	46.05	0.89	(44.34, 47.85)
VAE	unimodal	44.37	0.88	(42.71, 46.16)
VAE	multimodal	51.66	0.97	(49.81, 53.62)

Table S39: **Comparison of expected Chi-Square statistics of GWAS Catalog variants by different models and learning methods on lead I ECG + PPG dataset.** CI: confidence interval.

See the attached Excel table.

Table S40: **M-REGLE loci improves phenotypic prediction compared to unimodal loci.**

Pheno	U-REGLE		M-REGLE	
	Standard	Refined	Standard	Refined
Afib	0.57 (0.56, 0.58)	0.57 (0.56, 0.58)	0.59 (0.58, 0.60)	0.59 (0.58, 0.60)
MI	0.52 (0.51, 0.53)	0.53 (0.52, 0.54)	0.53 (0.52, 0.54)	0.53 (0.53, 0.55)
HTN	0.52 (0.51, 0.52)	0.52 (0.51, 0.52)	0.52 (0.52, 0.53)	0.52 (0.52, 0.53)
Stroke	0.50 (0.49, 0.52)	0.51 (0.50, 0.52)	0.50 (0.49, 0.52)	0.52 (0.51, 0.53)

Table S41: **AUROC comparison of M-REGLE and U-REGLE for standard and refined disease for 12-lead ECG.** We defined a refined version of the reference group for all diseases (e.g., Afib, myocardial infarction, hypertension, stroke), where the cases are again individuals who have the disease, but the controls are individuals who do not have any cardiovascular disease.

Pheno	U-REGLE		M-REGLE	
	Standard	Refined	Standard	Refined
Aifb	0.09 (0.08, 0.09)	0.14 (0.13, 0.14)	0.10 (0.09, 0.10)	0.15 (0.14, 0.16)
MI	0.06 (0.06, 0.07)	0.10 (0.09, 0.10)	0.06 (0.06, 0.07)	0.10 (0.10, 0.11)
HTN	0.41 (0.40, 0.42)	0.43 (0.42, 0.44)	0.41 (0.41, 0.42)	0.43 (0.42, 0.44)
Stroke	0.04 (0.03, 0.04)	0.06 (0.06, 0.07)	0.04 (0.03, 0.04)	0.07 (0.06, 0.07)

Table S42: **AUPRC comparison of M-REGLE and U-REGLE for standard and refined disease 12-lead ECG.** We defined a refined version of the reference group for all diseases (e.g., Afib, myocardial infarction, hypertension, stroke), where the cases are again individuals who have the disease, but the controls are individuals who do not have any cardiovascular disease.

Pheno	U-REGLE		M-REGLE	
	Standard	Refined	Standard	Refined
Aifb	0.56 (0.55, 0.57)	0.56 (0.55, 0.57)	0.56 (0.55, 0.57)	0.56 (0.55, 0.57)
MI	0.51 (0.50, 0.52)	0.51 (0.50, 0.52)	0.51 (0.49, 0.52)	0.51 (0.50, 0.53)
HTN	0.51 (0.51, 0.52)	0.52 (0.51, 0.52)	0.51 (0.51, 0.52)	0.51 (0.51, 0.52)
Stroke	0.51 (0.50, 0.52)	0.51 (0.49, 0.52)	0.51 (0.49, 0.52)	0.51 (0.49, 0.52)

Table S43: **AUROC comparison of M-REGLE and U-REGLE for standard and refined disease for ECG lead I + PPG.** We defined a refined version of the reference group for all diseases (e.g., Afib, myocardial infarction, hypertension, stroke), where the cases are again individuals who have the disease, but the controls are individuals who do not have any cardiovascular disease.

Pheno	M-REGLE		U-REGLE	
	Standard	Refined	Standard	Refined
Afib	0.08 (0.08, 0.09)	0.13 (0.13, 0.14)	0.08 (0.08, 0.09)	0.13 (0.13, 0.14)
MI	0.06 (0.06, 0.06)	0.09 (0.09, 0.10)	0.06 (0.06, 0.06)	0.10 (0.09, 0.10)
HTN	0.41 (0.40, 0.41)	0.42 (0.42, 0.43)	0.40 (0.40, 0.41)	0.42 (0.42, 0.43)
Stroke	0.04 (0.03, 0.04)	0.06 (0.06, 0.07)	0.04 (0.03, 0.04)	0.06 (0.06, 0.07)

Table S44: **AUPRC comparison of M-REGLE and U-REGLE for standard and refined disease ECG lead I + PPG.** We defined a refined version of the reference group for all diseases (e.g., Afib, myocardial infarction, hypertension, stroke), where the cases are again individuals who have the disease, but the controls are individuals who do not have any cardiovascular disease.

Method	AUROC	AUPRC	Top 1% prevalence	Top 5% prevalence
M-REGLE	0.55 (0.53-0.57) *	0.22 (0.21-0.24)	0.21 (0.10-0.34)	0.24 (0.18-0.30)
U-REGLE	0.53 (0.51-0.55)	0.21 (0.20-0.23)	0.27 (0.17-0.40)	0.25 (0.19-0.31)

Table S45: **Comparison of M-REGLE vs U-REGLE Afib prediction in Indiana Biobank dataset.** Star (*) indicates M-REGLE is significantly better than U-REGLE based on paired bootstrap test.

Method	AUROC	AUPRC	Top 1% prevalence	Top 5% prevalence
M-REGLE	0.58 (0.56-0.59)*	0.21 (0.20-0.22)*	0.25 (0.19-0.32)	0.24 (0.22-0.27)*
U-REGLE	0.55 (0.54-0.56)	0.19 (0.18-0.20)	0.22 (0.16-0.29)	0.20 (0.18-0.23)

Table S46: **Comparison of M-REGLE vs U-REGLE Afib prediction in EPIC-Norfolk dataset.** Star (*) indicates M-REGLE is significantly better than U-REGLE based on paired bootstrap test.

Phenotype	Method	Pearson R
PPG pulse rate	M-REGLE	0.10 (0.08-0.11)
PPG pulse rate	U-REGLE	0.09 (0.08-0.10)
SBP	M-REGLE	0.03 (0.01-0.04)
SBP	U-REGLE	0.02 (0.00-0.03)

Table S47: **Comparison of M-REGLE vs U-REGLE PPG pulse rate, ECG QT interval, and SBP prediction in EPIC-Norfolk dataset.**

Method	AUROC	AUPRC	Top 1% prevalence	Top 5% prevalence
M-REGLE	0.55 (0.52-0.58)	0.11 (0.09-0.13)*	0.16 (0.08-0.28)	0.16 (0.12-0.21)*
U-REGLE	0.53 (0.50-0.56)	0.10 (0.08-0.11)	0.09 (0.03-0.22)	0.09 (0.05-0.13)

Table S48: **Comparison of M-REGLE vs U-REGLE Afib prediction in BWHHS dataset.** Star (*) indicates M-REGLE is significantly better than U-REGLE based on paired bootstrap test.

Phenotype	Method	Pearson R
QT interval	M-REGLE	0.003 (-0.030-0.036)
QT interval	U-REGLE	-0.002 (-0.036-0.036)
PPG pulse rate	M-REGLE	0.08 (0.046-0.115)
PPG pulse rate	U-REGLE	-0.020 (-0.054-0.014)
SBP	M-REGLE	0.028 (-0.006-0.061)
SBP	U-REGLE	0.034 (0.0003-0.066)

Table S49: **Comparison of M-REGLE vs U-REGLE PPG pulse rate, ECG QT interval, and SBP prediction in BWHHS dataset.**

Variance explained	Multimodal	Unimodal
70%	6.17e-04 ± 6.07e-06	8.26e-04 ± 1.54e-05
83%	3.00e-04 ± 2.91e-06	2.71e-04 ± 2.43e-06
93%-94%	1.17e-04 ± 1.32e-07	1.27e-04 ± 1.12e-06
96%	7.2e-05 ± 1.04e-06	8.8e-05 ± 9.39e-07
97%	6.7e-05 ± 1.12e-06	8.1e-05 ± 8.1e-07
98%	6.4e-05 ± 8.94e-07	7.1e-05 ± 7.59e-07
99%	6.4e-05 ± 9.07e-07	7.2e-05 ± 7.11e-07

Table S50: **Comparison of multimodal reconstruction losses and an average of all unimodal reconstruction losses across different latent dimension numbers on lead I ECG + PPG + Spirogram dataset.**

Pheno	AUROC	AUPRC	Top 1% prevalence	Top 10% prevalence
Hypertension	0.64 (0.62, 0.66)	0.44 (0.42, 0.47)	0.53 (0.49, 0.57)	0.61 (0.47, 0.73)
Diabetes type 2	0.69 (0.65, 0.73)	0.11 (0.09, 0.13)	0.13 (0.10, 0.15)	0.22 (0.13, 0.31)
Cardiovascular disease	0.63 (0.61, 0.64)	0.45 (0.43, 0.48)	0.54 (0.50, 0.58)	0.56 (0.45, 0.69)
Myocardial infarction	0.63 (0.59, 0.67)	0.06 (0.05, 0.07)	0.06 (0.05, 0.09)	0.08 (0.02, 0.15)
Afib	0.65 (0.62, 0.69)	0.07 (0.05, 0.08)	0.08 (0.06, 0.09)	0.08 (0.02, 0.17)
Coronary artery disease	0.62 (0.59, 0.65)	0.11 (0.08, 0.13)	0.12 (0.09, 0.15)	0.24 (0.14, 0.36)
Stroke	0.57 (0.51, 0.62)	0.03 (0.02, 0.05)	0.04 (0.02, 0.05)	0.02 (0.00, 0.08)
Asthma	0.66 (0.63, 0.67)	0.27 (0.23, 0.29)	0.34 (0.30, 0.37)	0.43 (0.25, 0.53)
Angina	0.58 (0.55, 0.61)	0.07 (0.06, 0.09)	0.08 (0.06, 0.10)	0.13 (0.07, 0.23)
All cause mortality	0.58 (0.53, 0.64)	0.03 (0.02, 0.04)	0.03 (0.01, 0.04)	0.06 (0.02, 0.11)

Table S51: **Prediction of U-REGLE embeddings from ECG lead I + PPG + Spirograms.**

Pheno	AUROC	AUPRC	Top 1% prevalence	Top 10% prevalence
hypertension	0.66 (0.65, 0.68)	0.47 (0.45, 0.49)	0.56 (0.52, 0.60)	0.67 (0.55, 0.78)
diabetes type 2	0.70 (0.67, 0.74)	0.12 (0.09, 0.15)	0.13 (0.10, 0.15)	0.23 (0.14, 0.35)
cardiovascular disease	0.65 (0.64, 0.67)	0.48 (0.46, 0.51)	0.57 (0.53, 0.61)	0.64 (0.51, 0.76)
myocardial infarction	0.65 (0.61, 0.68)	0.07 (0.05, 0.10)	0.06 (0.05, 0.09)	0.14 (0.07, 0.24)
afib	0.66 (0.62, 0.70)	0.07 (0.06, 0.09)	0.09 (0.07, 0.10)	0.08 (0.02, 0.15)
coronary artery disease	0.64 (0.61, 0.67)	0.10 (0.08, 0.13)	0.11 (0.09, 0.14)	0.23 (0.11, 0.34)
stroke	0.60 (0.54, 0.65)	0.03 (0.02, 0.05)	0.03 (0.02, 0.04)	0.03 (0.00, 0.08)
asthma	0.67 (0.64, 0.68)	0.28 (0.25, 0.30)	0.36 (0.32, 0.39)	0.46 (0.35, 0.58)
angina	0.62 (0.58, 0.65)	0.08 (0.07, 0.10)	0.10 (0.07, 0.12)	0.16 (0.08, 0.26)
all cause mortality	0.59 (0.53, 0.64)	0.03 (0.02, 0.04)	0.03 (0.02, 0.04)	0.04 (0.00, 0.10)

Table S52: **Prediction of M-REGLE embeddings from ECG lead I + PPG + Spirograms.**
